# Atorvastatin-pretreated mesenchymal stem cell-derived extracellular vesicles promote cardiac repair after myocardial infarction via shifting macrophage polarization by targeting microRNA-139-3p/Stat1 pathway

**DOI:** 10.1186/s12916-023-02778-x

**Published:** 2023-03-16

**Authors:** Yu Ning, Peisen Huang, Guihao Chen, Yuyan Xiong, Zhaoting Gong, Chunxiao Wu, Junyan Xu, Wenyang Jiang, Xiaosong Li, Ruijie Tang, Lili Zhang, Mengjin Hu, Jing Xu, Jun Xu, Haiyan Qian, Chen Jin, Yuejin Yang

**Affiliations:** 1 Department of Cardiology, The First Affiliated Hospital of Sun Yat-sen University, Guangzhou, China; 2 State Key Laboratory of Cardiovascular Disease, Department of Cardiology, Fuwai Hospital, National Center for Cardiovascular Diseases, Chinese Academy of Medical Science and Peking Union Medical College, No.167 North Lishi Road, Xicheng District, Beijing, 100037 China; 3 National Health Commission Key Laboratory of Assisted Circulation, Sun Yat-sen University, Guangzhou, China

**Keywords:** Atorvastatin, Mesenchymal stem cell, Extracellular vesicle, Macrophage polarization, Myocardial infarction

## Abstract

**Background:**

Extracellular vesicles (EVs) derived from bone marrow mesenchymal stem cells (MSCs) pretreated with atorvastatin (ATV) (MSC^ATV^-EV) have a superior cardiac repair effect on acute myocardial infarction (AMI). The mechanisms, however, have not been fully elucidated. This study aims to explore whether inflammation alleviation of infarct region via macrophage polarization plays a key role in the efficacy of MSC^ATV^-EV.

**Methods:**

MSC^ATV^-EV or MSC-EV were intramyocardially injected 30 min after coronary ligation in AMI rats. Macrophage infiltration and polarization (day 3), cardiac function (days 0, 3, 7, 28), and infarct size (day 28) were measured. EV small RNA sequencing and bioinformatics analysis were conducted for differentially expressed miRNAs between MSC^ATV^-EV and MSC-EV. Macrophages were isolated from rat bone marrow for molecular mechanism analysis. miRNA mimics or inhibitors were transfected into EVs or macrophages to analyze its effects on macrophage polarization and cardiac repair in vitro and in vivo.

**Results:**

MSC^ATV^-EV significantly reduced the amount of CD68^+^ total macrophages and increased CD206^+^ M2 macrophages of infarct zone on day 3 after AMI compared with MSC-EV group (*P* < 0.01–0.0001). On day 28, MSC^ATV^-EV much more significantly improved the cardiac function than MSC-EV with the infarct size markedly reduced (*P* < 0.05–0.0001). In vitro, MSC^ATV^-EV also significantly reduced the protein and mRNA expressions of M1 markers but increased those of M2 markers in lipopolysaccharide-treated macrophages (*P* < 0.05–0.0001). EV miR-139-3p was identified as a potential cardiac repair factor mediating macrophage polarization. Knockdown of miR-139-3p in MSC^ATV^-EV significantly attenuated while overexpression of it in MSC-EV enhanced the effect on promoting M2 polarization by suppressing downstream signal transducer and activator of transcription 1 (Stat1). Furthermore, MSC^ATV^-EV loaded with miR-139-3p inhibitors decreased while MSC-EV loaded with miR-139-3p mimics increased the expressions of M2 markers and cardioprotective efficacy.

**Conclusions:**

We uncovered a novel mechanism that MSC^ATV^-EV remarkably facilitate cardiac repair in AMI by promoting macrophage polarization via miR-139-3p/Stat1 pathway, which has the great potential for clinical translation.

**Supplementary Information:**

The online version contains supplementary material available at 10.1186/s12916-023-02778-x.

## Background

Acute myocardial infarction (AMI) is a life-threatening disease because of loss of cardiac muscles and its exceedingly limited capacity of regeneration. Stem cells, especially bone marrow stem cell transplantation, have been embraced as a potential alternative to repair the broken heart. Although animal studies showed an effective improvement in cardiac function of AMI models with the stem cell therapy [1, 2], the weak efficacy of therapeutic benefit have been observed in clinical trials [3–5], which suggests that the effects of stem cell therapy remain to be enhanced. In recent years, the paracrine effect has been regarded as an attributor of cardioprotection in stem cell therapy, which is mainly mediated by extracellular vesicles (EVs) derived by stem cells [6–9]. According to the MISEV2018 guideline, EVs are the generic term for particles naturally released from the cells, which are delimited by a lipid bilayer and cannot replicate [10]. Referring to their size, EV subtypes are classified as small EVs (sEVs) (< 100 nm or < 200 nm) and medium/large EVs (> 200 nm) [10]. EVs carry abundant cargo of mRNAs, non-coding RNAs such as microRNAs (miRNAs), proteins, lipids, etc., from their parent cells [10–12]. These bioactive molecules in EV cargo and their roles in cell-cell communication have stoked up interest in stem cell-derived EVs as potential therapeutic tools for ischemic heart injury and ischemic-reperfusion injury [13–15]. Cell-free therapy with EVs not only has shown similar effects as stem cells in cardiac repair for AMI [16–18] but also has several advantages over cells, including product stability, immune tolerability, effectiveness through systemic delivery, and capability of crossing the blood-brain barrier [19]. However, the weak effectiveness of cell-free therapy still remains to be the main limitation as same as stem cell therapy itself is [20].

Our previous studies have found that mesenchymal stem cell (MSC) transplantation based on the pleiotropic effects of atorvastatin (ATV) can improve heart function after AMI [21–25]. ATV-pretreated MSCs (MSC^ATV^) also enhanced the effect of cardiac repair [26]. The combination of ATV treatment with MSC^ATV^ further augmented the therapeutic efficacy [23, 27]. Moreover, EVs derived from MSC^ATV^ (MSC^ATV^-EV) alone [28] or in combination with MSC^ATV^ [29] remarkably enhanced heart function and minimized infarct size due to angiogenesis via the upregulated lncRNA H19 in EVs. Besides H19, miRNAs may also be induced by ATV and exert an important role in the cardiac repair process of MSC^ATV^-EV. Therefore, we extend our work by further exploring the probable key roles of miRNAs contained in MSC^ATV^-EV in cardiac repair for AMI. Likewise, macrophages polarization have drawn much attention recently on their plasticity in ischemic heart disease [30, 31]. In the early stage of AMI, pro-inflammatory macrophages (M1) are the predominant subtype and responsible for cell debris clearance, while in the later stage, M2 macrophages start to take responsibility for the healing process of infarcted myocardium [32–35]. It is a promising strategy to modulate and balance macrophage polarization for healing the infarcted myocardium. Furthermore, we hypothesized that miRNAs in MSC^ATV^-EV might mediate the beneficial macrophage polarization in the setting of AMI.

This study aims to detect and screen the differentially expressed miRNAs in MSC^ATV^-EV through EV small RNA sequencing, and to explore the important roles of the key miRNAs in shifting macrophage polarization for myocardial repair after AMI. We found that EV transfer of miR-139-3p by MSC^ATV^-EV into macrophages targeted to inhibit signal transducer and activator of transcription 1 (Stat1) expression and activation to promote M2 macrophage polarization, remarkably enhancing the efficacy of cardiac repair for AMI.

## Methods

Detailed descriptions of experimental procedures are available in the Additional file 1: Supplementary data [23, 28, 29, 36].

### Primary cell isolation and culture

#### Rat bone marrow-derived MSCs

Bone marrow-derived MSCs were isolated as described previously [28]. Briefly, femurs and tibias from male Sprague-Dawley rats (60–80 g) were flushed with Iscove’s Modified Dulbecco’s Medium (IMDM, Invitrogen, USA) to collect total bone marrow cells, which were then cultured with IMDM containing 10% fetal bovine serum (FBS, Gibco, USA) and 1% penicillin/streptomycin (Gibco, USA). The medium was changed every 3 days along with non-adherent cells removed. MSCs were passaged when reached 80% to 90% confluence. MSCs at passages 3–4 were used for the study. For ATV pretreatment, MSCs were treated with 1 μmol/L ATV (Sigma, USA) in IMDM containing 10% FBS for 24 h. The concentration of ATV is based on our previous study [28]. The control groups were treated with equal volumes of dimethyl sulfoxide (DMSO). Then, cells were washed with phosphate buffer saline (PBS) three times and cultured with fresh FBS-free IMDM for another 48 h. The conditioned supernatant was collected for EV isolation.

#### Rat bone marrow-derived macrophages (BMDMs)

Total bone marrow cells were harvested by the same method and cultured with complete Dulbecco’s modified eagle medium containing 20 ng/mL recombinant macrophage colony stimulating factor (CSF) (PeproTech) [37]. Six days later, the differentiated macrophages (BMDMs) were generated and then incubated with 100 ng/mL lipopolysaccharide (LPS) to induce M1 populations or with 10 ng/mL interleukin (IL)-4 plus 10 ng/ml IL-13 to induce M2 populations for at least 24 h [37, 38]. For EV incubation experiments, we used three different concentrations (2 μg/mL, 20 μg/mL, 40 μg/mL) of MSC-derived EVs (MSC-EV) or MSC^ATV^-EV to treat BMDMs for 24 h and then examined the expression of M2 macrophage marker to select an optimal concentration. To better mimic the inflammatory microenvironment of infarcted hearts, we further stimulated BMDMs with 100 ng/mL LPS for 6 h and then incubated the cells with the optimal concentration of MSC-EV or MSC^ATV^-EV for another 48 h.

### EV isolation

We used differential centrifugation to isolate EVs as described before [28, 39]. In brief, after MSCs were treated with ATV for 24 h and/or transfected with miR-139-3p mimics or inhibitors for 10 h, the conditioned medium was harvested for further centrifugation. Every step of centrifugation was under 4 °C condition. The conditioned medium was first centrifuged at 300 g for 10 min and then 2000 g for 20 min to remove cells and debris, which was followed by centrifugation at 16500 g for 30 min to remove macrovesicles. Then, the supernatant was ultracentrifuged twice at 120,000 g for 70 min. The final EV pellets were resuspended with 50–100 μL PBS and stored at – 80 °C. MicroBCA protein assay kit (Thermo Scientific, San Jose, CA) was used to test the protein concentrations of EVs.

### Rat AMI model

In vivo, animal experiments were performed on 6- to 8-week-old littermate male Sprague-Dawley rats. To induce AMI model, rats were anesthetized, ventilated via tracheal intubations connected to a rodent ventilator, and then performed with a thoracotomy to expose the heart [17, 28]. The left anterior descending coronary artery was ligated with a 6-0 silk suture. The induction of AMI was verified by color change to pale in the region below the ligation site. Thirty minutes later, various EVs (105 μg, in 100 μL PBS) or an equivalent volume of PBS were injected at three different points around the border zone of infarction [28]. The concentration of EVs used in our animal experiments refers to the report by de Couto et al. [17]. All animal studies were approved by the Fuwai Hospital Experimental Animals Ethics Committee, which complies with the NIH Guide for the Care and Use of Experimental Animals. The ARRIVE guidelines were followed (Additional file 2).

### Cardiac function measurements

Transthoracic echocardiography imaging system (Vevo 2000, Visual Sonics, Toronto, Canada) was used with two-dimensional M-mode analysis to evaluate rat cardiac function at baseline (before AMI, day 0), and days 3, 7, and 28 after AMI. Three consecutive cycles for each animal and time point were captured to measure left ventricular end-systolic dimension (LVESd) and left ventricular end-diastolic dimension (LVEDd). Left ventricular ejection fraction (LVEF), left ventricular fractional shortening (LVFS), left ventricular end-systolic volume (LVESV), and left ventricular end diastolic volume (LVEDV) were calculated and then averaged as previously described [26].

### Histological analysis

On days 3, 7, and 28 after AMI, animals were anesthetized with isoflurane to harvest hearts for histological analysis. The animal hearts were arrested in diastole by perfusing with 5% KCl. Heart tissues in the upper part of ligation were excised. The hearts were fixed in 4% paraformaldehyde for 24 h, embedded in paraffin, and cut into 4 μm-thick serial sections. The heart tissue sections were stained with Masson’s trichrome kit following the manufacturer’s instructions (Sigma) to evaluate the infarct size and myocardial fibrosis. The percentage of infarct area was calculated by the ratio of fibrotic area (blue) to entire LV cross-sectional area measured by the Image J software (Version 1.52a).

### EV small RNA sequencing

MSC-EV and MSC^ATV^-EV were isolated. The isolation of total RNA in the EVs, small RNA library construction, and EV small RNA sequencing were conducted by RiboBio Company (Guangzhou, China). Briefly, total RNAs of EVs were extracted by HiPure Liquid RNA/miRNA Kit (Magen, Guangzhou, China). The RNAs were ligated with 3′ and 5′ adapter at first, then reverse transcribed to cDNA and further amplified by quantitative polymerase chain reaction (qPCR). The PCR products were purified by electrophoresis following instructions of NEBNext® Multiplex Small RNA Library Prep Set for Illumina® (NEB, USA). The purified small RNA library products were evaluated by Qubit®2.0 (Life Technologies, USA) or Agilent 2200 TapeStation (Agilent Technologies, USA) and sequenced using Illumina HiSeq™ 2500 platform.

Raw reads of the small RNA sequencing were filtered by removal of the adapters, poly ‘N’, reads with less than 17 nucleotide or low quality, etc., to obtain the clean reads. The clean reads were aligned to rat reference genome to map the whole genome distribution and to several non-coding RNA (ncRNA) databases (miRBase, Rfam, and piRNABank) to annotate various ncRNA (miRNA, rRNA, tRNA, snRNA, snoRNA, Y_RNA and piRNA) [40–42]. miRNA expressions were calculated by reads per million (RPM) values: RPM = (number of reads mapping to miRNA/number of reads in clean data) × 10^6^. Differential miRNA expression between two sets of samples was calculated by edgeR algorithm according to the criteria of |log2^(Fold Change)^| ≥ 1 and *P* value < 0.05. TargetScan, miRDB, miRTarBase, and miRWalk were used to predict targets gene of selected miRNA and to do further gene ontology (GO) and Kyoto Encyclopedia of Genes and Genomes (KEGG) pathway enrichment analysis.

### miRNA transfections

Lipofectamine 3000 (Invitrogen, USA), miRNA mimics, or inhibitors (GenePharma, Shanghai) were diluted with opti-MEM respectively. Then, mix the diluted lipofectamine 3000 with miRNA mimics or inhibitors and incubate them in room temperature for 20 min. Add the mixture into medium of cultured MSCs or BMDMs for 10 h to obtain MSC-EV-miR mimic or MSC^ATV^-EV-miR inhibitor as described above for mechanistic experiments.

### Statistical analysis

Data were presented with mean ± standard deviation (SD). Differences of continuous variables among groups were tested by one-way ANOVA followed by Tukey’s multiple comparisons test. Statistical analyses were carried out using the Graphpad Prism 7.0 software. A *P* value < 0.05 was considered significant.

## Results

### Characterization and optimal concentration of MSC^ATV^-EV

**Fig. 1:**
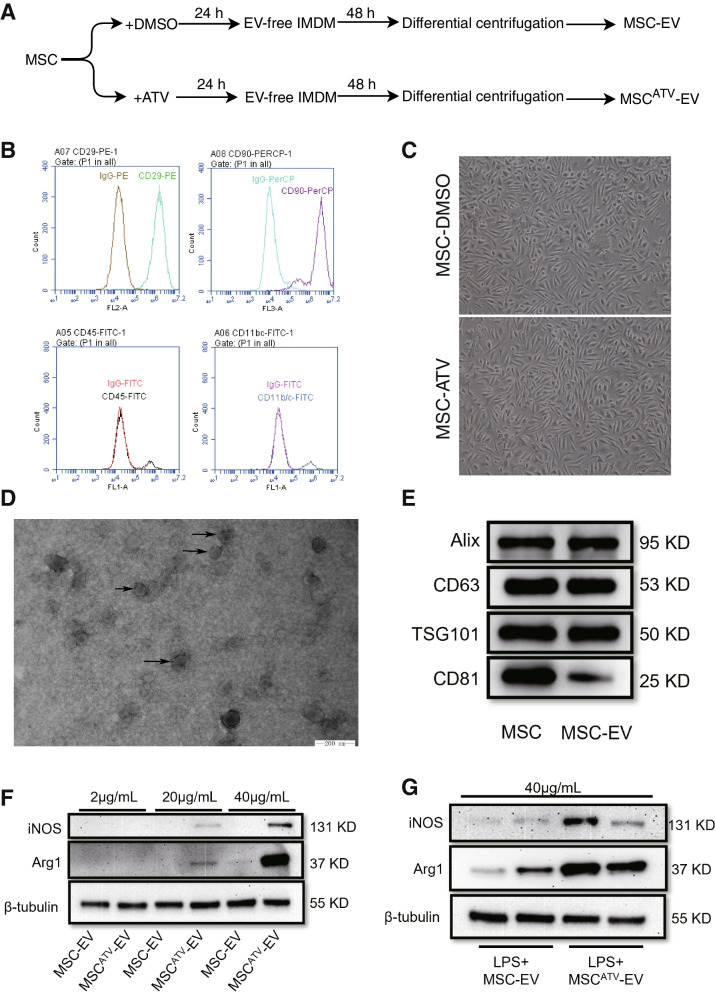
The characterization of atorvastatin-pretreated bone marrow MSC-derived EVs (MSC^ATV^-EV) in rats. **A** The schematic protocol of EVs isolated from rat bone marrow MSCs with or without ATV pretreatment. **B** Flow cytometry analysis of surface markers on rat bone marrow MSCs. **C** Morphology of MSCs treated with DMSO or ATV under the microscope. **D** Transmission electron microscopy scanning for the morphology of EVs. **E** Western blot analysis for the protein markers of EVs. **F** Western blot analysis for the protein expressions of Arg1 (a marker of M2 macrophages) and iNOS (a marker of M1 macrophages) in rat BMDMs treated with different concentrations (2 μg/mL, 20 μg/mL, 40 μg/mL) of MSC-EV or MSC^ATV^-EV for 24 h. **G** Western blot analysis for the protein expressions of Arg1 and iNOS in rat BMDMs stimulated with LPS for 6 h and then incubated with 40 μg/mL MSC-EV or MSC^ATV^-EV for another 48 h. Arg1, arginase 1; ATV, atorvastatin; BMDMs, bone marrow-derived macrophages; DMSO, dimethyl sulfoxide; EV, extracellular vesicle; IMDM, Iscove’s Modified Dulbecco’s Medium; iNOS, inducible nitric oxide synthase; LPS, lipopolysaccharide; MSCs, mesenchymal stem cells

MSCs were obtained from rat bone marrow and then pretreated with ATV for 24 h to isolate MSC^ATV^-EV (Fig. 1A). Flow cytometry showed the isolated passage 3-4 rat MSCs highly expressed surface markers CD29 and CD90 with no difference in morphology after treatment with ATV or DMSO (Fig. 1B, C). The cell viability of MSC or MSC^ATV^ was in a similarly good condition after 72 h treatment (Additional file 3: Fig. S1). MSC-derived EVs displayed a cup-shaped membrane structure under transmission electron microscopy with a diameter of 100–200 nm (Fig. 1D), which are classified as sEVs, and expressed the marker proteins Alix, CD63, TSG101, and CD81 (Fig. 1E) [43].

To determine the effective concentration of EVs in vivo, we used different concentrations (2 μg/mL, 20 μg/mL, 40 μg/mL) of MSC-EV or MSC^ATV^-EV to treat rat BMDMs for 24 h. The results of western blot showed that 40 μg/mL MSC^ATV^-EV significantly increased the protein expression of arginase 1 (Arg1, a marker of M2 macrophages [38]) and decreased that of inducible nitric oxide synthase (iNOS, a marker of M1 macrophages [38]) (Fig. 1F). To simulate the inflammatory microenvironment during myocardial infarction, we further stimulated BMDMs with 100 ng/mL LPS for 6 h and then treated the cells with 40 μg/mL MSC-EV or MSC^ATV^-EV for another 48 h. The results showed that MSC^ATV^-EV more significantly increased the protein expression of Arg1 and decreased that of iNOS than MSC-EV (Fig. 1G). Therefore, we chose the latter protocol with an EV concentration of 40 μg/mL in the following macrophage polarization experiments.

### The effects of MSC^ATV^-EV on enhancing cardiac function and promoting M2 macrophage polarization in AMI hearts

**Fig. 2:**
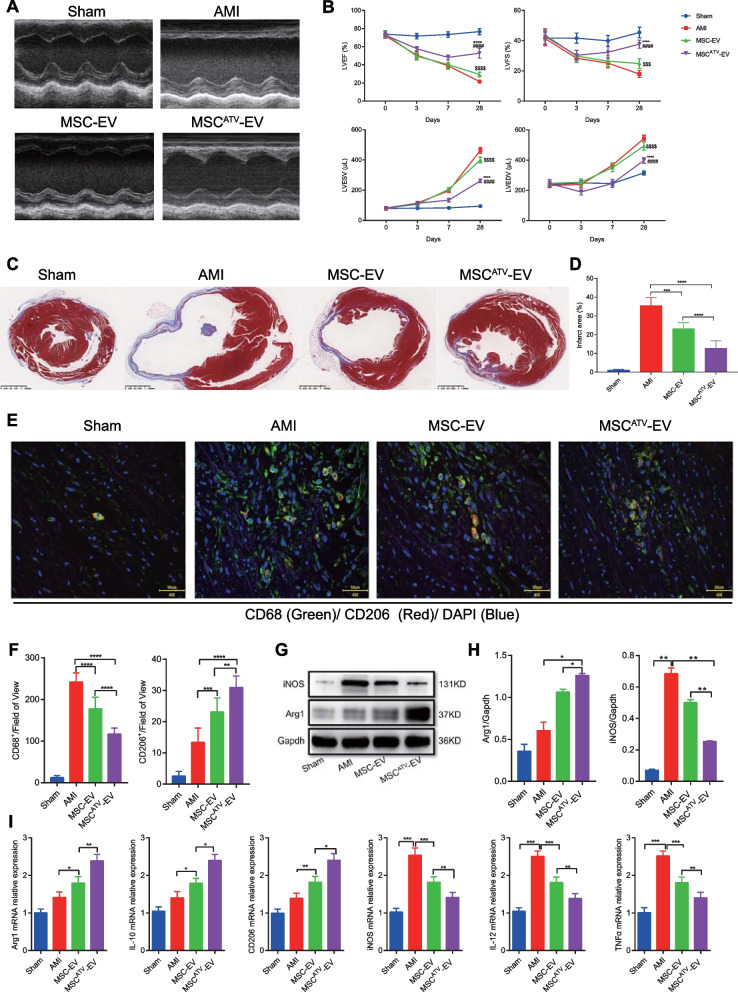
The superior effects of MSC^ATV^-EV on ventricular function, MI size, macrophage infiltration and M2 polarization in AMI rats. **A** Representative M-mode echocardiogram of each group on day 28 after AMI. **B** The dynamic changes in LVEF, LVFS, LVESV, and LVEDV of each group on days 0, 3, 7, 28 after AMI, *n* = 6–7 per group. **C** Representatives of heart cross sections with Masson trichrome staining on day 28. **D** Quantification of infarct size of each group in C, *n* = 6–7 per group. **E** Representative confocal images of CD68^+^ or CD206^+^ macrophages within the infarct border zone on day 3 post-AMI. **F** Quantification of CD68^+^ or CD206^+^ macrophages in E, *n* = 6-7 per group. **G** Western blot analysis of iNOS and Arg1 expressions in infarcted myocardium of each group. **H** Quantification of iNOS and Arg1 expressions in G, *n* = 3 per group. **I** The mRNA expressions of M1 (iNOS, IL-12, TNF-α) and M2 (Arg1, IL-10, CD206) macrophage markers in infarcted zone measured by qPCR, *n* = 3–4 per group. *P < 0.05, **P < 0.01, ***P < 0.001, ****P < 0.0001. Arg1, arginase 1; AMI, acute myocardial infarction; ATV, atorvastatin; EV, extracellular vesicle; IL, interleukin; iNOS, inducible nitric oxide synthase; LVEDV, left ventricular end diastolic volume; LVEF, left ventricular ejection fraction; LVESV, left ventricular end-systolic volume; LVFS, left ventricular fractional shortening; MSCs, mesenchymal stem cells; qPCR, quantitative polymerase chain reaction; TNF-α, tumor necrosis factor alpha

AS shown in Fig. 2A, B and Additional file 1: Table S1, MSC^ATV^-EV significantly enhanced both LVEF and LVFS values compared with both groups of MSC-EV and AMI (52.89 ± 5.40% vs. 29.31 ± 2.64% and 21.66% ± 1.54%, 37.70 ± 2.73% vs. 24.86 ± 2.10% and 17.95% ± 2.33%, respectively; all *P* < 0.0001) on day 28 post-AMI with both of LVESV and LVEDV significantly reduced (all *P* < 0.0001). MSC^ATV^-EV also significantly diminished infarct size in comparison with both groups of MSC-EV and AMI (10.22 ± 2.82% vs. 18.12 ± 4.64% and 30.06% ± 6.84%, *P* < 0.05 and *P* <0.0001, respectively) on day 28 (Fig. 2C, D). These results indicated that MSC^ATV^-EV were able to improve cardiac function, attenuate left ventricular remodeling, and reduce the infarct size much more effectively than MSC-EV.

To explore whether MSC^ATV^-EV treatment affects the accumulation and polarization of macrophages, we labeled total macrophages with CD68 and M2 macrophages with CD206 in the infarcted region on day 3 post-AMI by immunofluorescence. The results showed that MSC^ATV^-EV significantly decreased the CD68^+^ macrophage infiltration within the infarct border zone compared with both MSC-EV and AMI groups (both *P* < 0.0001) with CD206^+^ (M2) macrophages infiltration significantly increased (*P* < 0.01–0.0001) (Fig. 2E, F; Additional file 1: Table S1). Next, we detected the protein and mRNA expressions of M1 and M2 macrophage markers in the infarcted myocardium. Compared with both groups of MSC-EV and AMI, MSC^ATV^- EV significantly increased the protein expression of Arg1 but decreased that of iNOS (*P* < 0.05–0.01) (Fig. 2G, H; Additional file 1: Table S1). Furthermore, the mRNA expressions of Arg1, IL-10, and CD206 were also more significantly elevated and those of iNOS, IL-12, and tumor necrosis factor alpha (TNF-α) more significantly attenuated in MSC^ATV^-EV group than both groups of MSC-EV and AMI (*P* < 0.05–0.001) (Fig. 2I; Additional file 1: Table S1). These results suggested that MSC^ATV^-EV not only significantly reduced macrophage infiltration but also increased the proportion of M2 macrophages in the acute phase of the AMI region.

### MSC^ATV^-EV potently transformed M1 macrophages to M2 phenotype in vitro

**Fig. 3:**
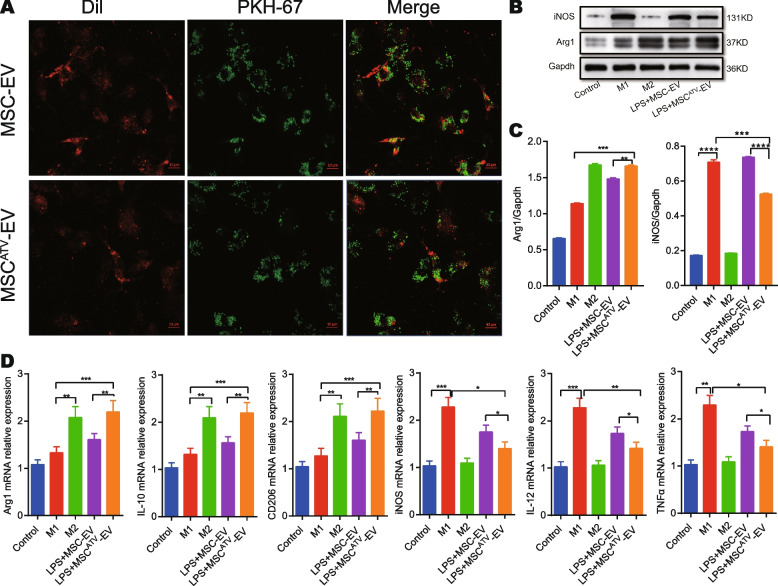
The effects of MSC^ATV^-EV and MSC-EV on macrophage polarization shifts in vitro. **A** PKH-67 labeled MSC^ATV^-EV or MSC-EV (green) intake by rat BMDMs (red). **B** Western blot analysis for protein expressions of iNOS and Arg1 in BMDMs. **C** Quantification of iNOS and Arg1 expressions in B, *n* = 3 per group. **D** qPCR analysis for mRNA expressions of macrophage markers (M1: iNOS, IL-12, TNF-α; M2: Arg1, IL-10, CD206) in BMDMs described in B, *n* = 4 per group. *P < 0.05, **P < 0.01, ***P < 0.001, ****P < 0.0001. Arg1, arginase 1; AMI, acute myocardial infarction; ATV, atorvastatin; BMDMs, bone marrow-derived macrophages; EV, extracellular vesicle; IL, interleukin; iNOS, inducible nitric oxide synthase; LPS, lipopolysaccharide; MSCs, mesenchymal stem cells; qPCR, quantitative polymerase chain reaction; TNF-α, tumor necrosis factor alpha

In vitro, we first examined the internalization of MSC-EV or MSC^ATV^-EV by rat BMDMs. Immunocytochemistry images showed that BMDMs efficiently internalized PKH67-labeled MSC-EV or MSC^ATV^-EV at 4 h after incubation (Fig. 3A). High-content screening system dynamically observed the intake of EVs by BMDM (Additional file 4: Supplementary Video). Then, we induced M1 and M2 macrophage phenotypes to compare the effects of MSC^ATV^-EV and MSC-EV on macrophage polarization. Compared with control group, the significantly elevated protein and/or mRNA expressions of iNOS, IL-12, or TNF-α in M1 group (all *P* < 0.0001) represented the successful formation of M1 macrophage model in vitro, and those of Arg1, IL-10, or CD206 in M2 group (all *P* < 0.0001) suggested the formation of M2 macrophage model (Fig. 3B–D; Additional file 1: Table S2). Furthermore, compared with M1 BMDMs group, the protein expression of Arg1 was significantly increased in both LPS+MSC- EV and LPS+MSC^ATV^-EV BMDMs groups (*P* < 0.01 and *P* < 0.001, respectively) with the latter increased more than the former group (*P* < 0.01), while that of iNOS decreased only in the LPS+MSC^ATV^-EV group (*P* < 0.001) indicating more potent effect of MSC^ATV^-EV than MSC-EV on M1 to M2 macrophage polarization (Fig. 3B, C; Additional file 1: Table S2). Similarly, LPS+MSC^ATV^-EV also more significantly elevated the mRNA expressions of M2 markers (Arg1, IL-10, and CD206; *P* < 0.01-0.001) but more significantly reduced those of M1 markers (iNOS, IL-12, and TNF-α; all *P* < 0.05) than LPS+MSC-EV, (Fig. 3D; Additional file 1: Table S2). These data suggested that MSC^ATV^-EV more efficiently shifted macrophage polarization from M1 to M2 than MSC-EV in vitro.

### MSC^ATV^-EV have a distinct miRNA signature

**Fig. 4:**
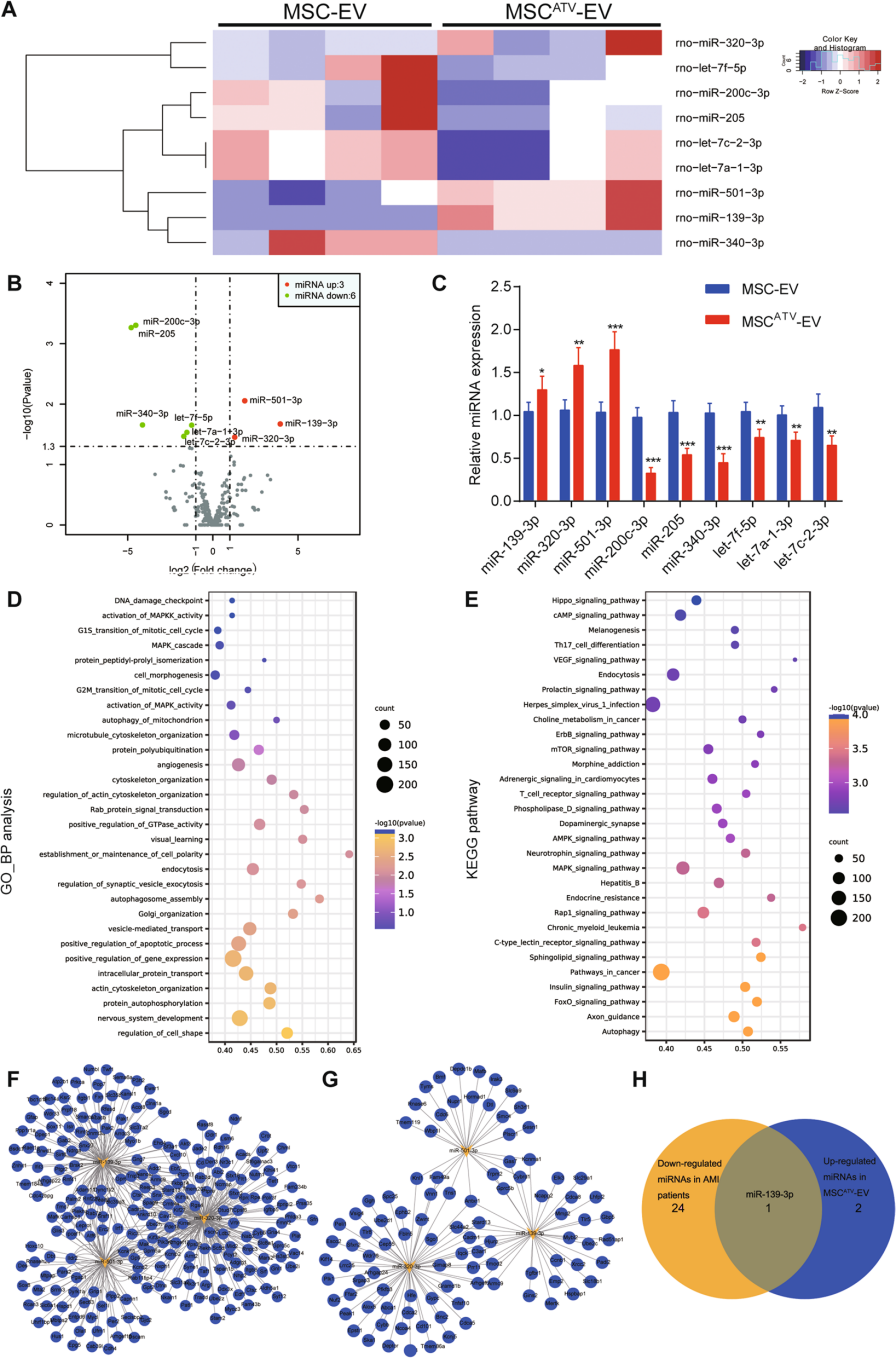
Small RNA sequencing analysis identifies miR-139-3p as an extracellular vesicle miRNA candidate. **A**, **B** Hierarchical clustering heat map (**A**) and volcano plot (**B**) showing 9 differentially expressed miRNAs between MSC-EV and MSC^ATV^–EV. **C** Verification for the mRNA expressions in MSC-EV and MSC^ATV^–EV by qPCR analysis, *n* = 3–4 per group. *P < 0.05, **P < 0.01, ***P < 0.001. **D**, **E** GO (**D**) and KEGG (**E**) enrichment analysis of target genes of three upregulated miRNAs (miR-139-3p, miR-320-3p, miR-501-3p) between MSC-EV and MSC^ATV^–EV. **F** Interaction network between target genes of three upregulated miRNAs (miR-139-3p, miR-320-3p, miR-501-3p) and upregulated genes in patients suffered from first acute myocardial infarction (GEO: GSE24591). **G** Interaction network between target genes of three upregulated miRNAs (miR-139-3p, miR-320-3p, miR-501-3p) and downregulated genes in human monocyte-derived M2 macrophages (GEO: GSE32164). **H** The intersection between the three upregulated miRNAs of miR-139-3p, miR-320-3p, miR-501-3p, and 25 downregulated miRNAs in patients suffered from first acute myocardial infarction (GEO: GSE24591) is miR-139-3p. ATV, atorvastatin; EV, extracellular vesicle; GEO, gene expression omnibus; GO, gene ontology; KEGG, Kyoto Encyclopedia of Genes and Genomes; MSCs, mesenchymal stem cells; miRNA, micro-RNA; qPCR, quantitative polymerase chain reaction

The RNA cargo of EVs varies with cell origin and environmental stimulus and alters recipient cell function [13]. To reveal the miRNA profiles in MSC^ATV^- EV and understand how they modulate macrophage polarization, we performed small RNA sequencing on MSC^ATV^-EV and MSC-EV. The molecule size ranges of RNA samples from MSC^ATV^-EV and MSC-EV were established by Agilent 2200 TapeStation Instrument (Additional file 5: Fig. S2). ncRNA annotation showed that the read accounts mapping to diverse types of ncRNA were similar between MSC^ATV^- EV and MSC- EV; most reads mapped to tRNA, piRNA, and rRNA, while fewer reads mapped to miRNA, snRNA, and snoRNA, etc. (Additional file 6: Fig. S3; Additional file 1: Table S3). We dug into the reads that aligned to miRNA annotations. Differential expression analysis found three upregulated (miR-139-3p, miR-320-3p, miR-501-3p) and six downregulated known mature miRNAs (miR-200c-3p, miR-205, miR-340-3p, let-7f-5p, let-7a-1-3p, let-7c-2-3p) in MSC^ATV^-EV compared with MSC-EV (Fig. 4A, B; Additional file 1: Table S4). qPCR analysis validated the significantly differential expressions of these nine miRNAs between MSC^ATV^-EV and MSC-EV (Fig. 4C; Additional file 1: Table S4).

To screen a miRNA that is both cardioprotective and polarization-related, we predicted target genes of the three upregulated miRNAs (miR-139-3p, miR-320-3p, miR-501-3p) using TargetScan, miRDB, miRTarBase, and miRWalk. GO and KEGG functional analysis on the target genes enriched in multiple pathways such as establishment or maintenance of cell polarity, angiogenesis, apoptosis, autophagy, and phagocytosis, etc. (Fig. 4D, E). Next, we retrieved two human datasets in the gene expression omnibus (GEO) database: GSE24591, a gene and miRNA expression profiling of patients affected by first AMI; GSE32164, a gene expression profiling of human monocyte-derived macrophage polarization. Comparing the target genes of the three miRNAs with the upregulated genes of AMI patients (GSE24591), we noticed that many of the target genes were upregulated in AMI patients relative to normal people (Fig. 4F; Additional file 7: Fig. S4). These results suggest that miR-139-3p, miR-320-3p, and miR-501-3p might be potential cardioprotective factors. Then, we compared the target genes with downregulated genes in human M2 macrophages (GSE32164). The results showed that multiple target genes of the three miRNAs were downregulated in human M2 macrophages, which implies miR-139-3p, miR-320-3p, and miR-501-3p possibly correlate with M2 polarization (Fig. 4G, Additional file 8: Fig. S5). To determine the most significant miRNA to study, we compared three upregulated miRNAs in MSC^ATV^-EV with 25 downregulated miRNAs in AMI patients (GSE24591) (Additional file 1: Table S5). Intriguingly, miR-139-3p stood out as the only one in their intersection (Fig. 4H). We searched another MI dataset GSE123342 to validate and also found that the expression of miR-139 decreased in AMI patients (Additional file 1: Table S6). Additional file 9: Fig. S6 showed the consensus sequence of miR-139 in miRNA-seq of MSC-EV and MSC^ATV^-EV mapped to rno-miR-139. These data indicate that miR-139-3p, upregulated in MSC^ATV^-EV, is likely to be a cardioprotective factor in AMI and promote M2 polarization.

### miR-139-3p is a mediator of MSC^ATV^-EV -modulated M2 macrophage polarization by targeting Stat1 in vitro

**Fig. 5:**
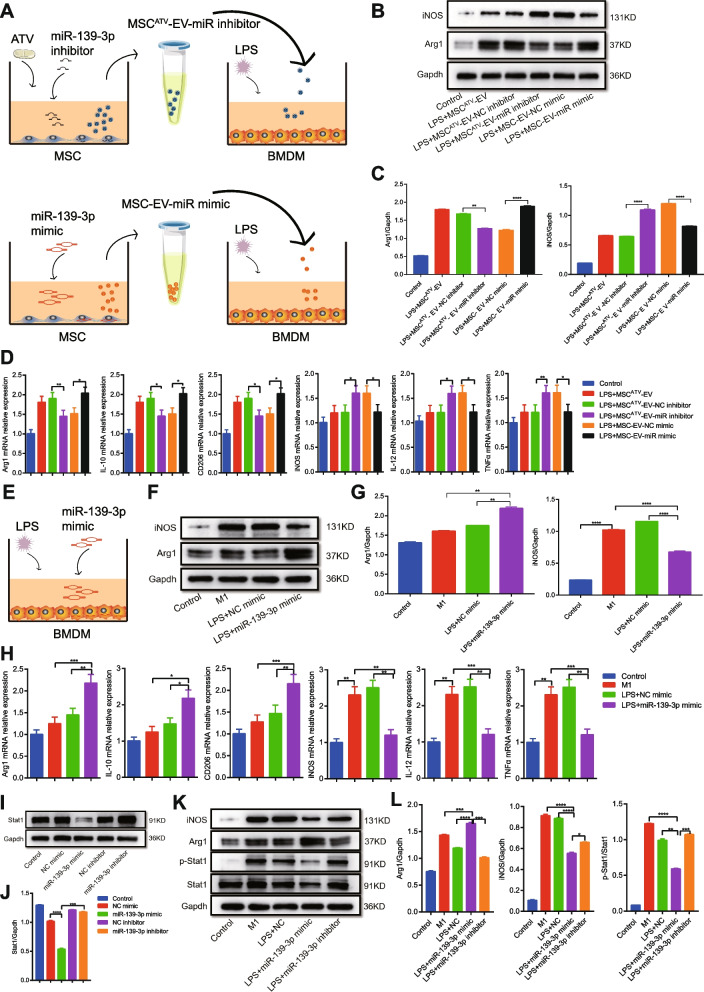
miR-139-3p is an effector of MSC^ATV^–EV-mediated macrophage polarization by targeting Stat1 in vitro. **A** Schematic protocol of isolating MSC^ATV^-EV-miR inhibitor and MSC-EV-miR mimic and incubating LPS-pretreated rat BMDMs. **B** The protein expressions of macrophage M1 marker iNOS and M2 marker Arg1 in the BMDMs as described in **A**. **C** Quantification of iNOS and Arg1 expressions in **B**, *n* = 3 per group. **D** qPCR analysis for the mRNA expressions of macrophage markers (M1: iNOS, IL-12, TNF-α; M2: Arg1, IL-10, CD206) in the BMDMs described in **A**, *n* = 3–4 per group. **E** Schematic protocol of miR-139-3p mimics being directly transfected into LPS-pretreated BMDMs. **F** The protein expressions of macrophage M1 marker iNOS and M2 marker Arg1 in the BMDMs described in **E**. **G** Quantification of iNOS and Arg1 expressions in **F**, *n* = 3 per group. **H** qPCR analysis for the mRNA expressions of macrophage markers (M1: iNOS, IL-12, TNF-α; M2: Arg1, IL-10, CD206) in the BMDMs described in **E**, *n* = 3–4 per group. *P < 0.05, **P < 0.01, ***P < 0.001, ****P < 0.0001. **I**, **J** The protein expression of Stat1 in rat BMDMs transfected with miR-139-3p mimics or inhibitors, *n* = 3 per group. **K**, **L** The protein expressions of iNOS, Arg1, and phosphorylated Stat1 in BMDMs stimulated with LPS and transfected with miR-139-3p mimics or inhibitors, *n* = 3 per group. *P < 0.05, **P < 0.01, ***P < 0.001, ****P < 0.0001. Arg1, arginase 1; ATV, atorvastatin; BMDMs, bone marrow-derived macrophages; EV, extracellular vesicle; IL, interleukin; iNOS, inducible nitric oxide synthase; LPS, lipopolysaccharide; MSCs, mesenchymal stem cells; miR, micro-RNA; qPCR, quantitative polymerase chain reaction; Stat1, signal transducer and activator of transcription 1; TNF-α, tumor necrosis factor alpha

To investigate the effect of EV miR-139-3p on macrophage polarization, we knocked down and overexpressed miR-139-3p in MSCs with or without ATV pretreatment, isolated corresponding EVs of MSC^ATV^-EV-miR inhibitor and MSC-EV-miR mimic, and incubated LPS-stimulated BMDMs with these EVs (Fig. 5A). Compared with LPS+MSC^ATV^-EV or LPS+MSC^ATV^-EV-NC inhibitor group, the protein expression of Arg1 in BMDMs was reduced but that of iNOS increased in LPS+MSC^ATV^-EV-miR inhibitor group; in contrast, LPS+MSC-EV-miR mimic treatment significantly enhanced the protein expression of Arg1 and attenuated that of iNOS in BMDMs relative to LPS+MSC-EV-NC mimic group (Fig. 5B, C; Additional file 1: Table S7). Similarly, knockdown of miR-139-3p in MSC^ATV^-EV decreased the mRNA expression of Arg1, IL-10, and CD206 but increased that of iNOS, IL-12, and TNF-α in LPS-stimulated BMDMs, while overexpression of miR-139-3p in MSC-EV elevated the mRNA expression of Arg1, IL-10, and CD206 but reduced that of iNOS, IL-12, and TNF-α (Fig. 5D; Additional file 1: Table S7). Furthermore, we directly transfected LPS-induced BMDMs with miR-139-3p mimic (Fig. 5E). Compared with M1 group, miR-139-3p mimic significantly increased the protein or mRNA expression of Arg1, IL-10, and CD206 but decreased that of iNOS, IL-12, and TNF-α in BMDMs (Fig. 5F–H; Additional file 1: Table S8). These results proved that upregulated miR-139-3p mediates MSC^ATV^-EV function on promoting M2 polarization.

To identify the target genes of miR-139-3p in MSC^ATV^-EV-treated macrophages, we predicted them in miRWalk, TargetScan, miRDB, and miRTarBase. Notably, TargetScan predicted that Stat1 is a possible target gene of miR-139-3p (Additional file 10: Fig. S7A; Additional file 1: Table S9). As is well known, Stat1 activation drives M1 macrophage polarization [44]. We further performed the luciferase reporter assay to verify the pairing of miR-139-3p and 3′UTR target region of Stat1 transcript. The results showed that miR-139-3p mimic decreased luciferase activity in Stat1 wild type 3′UTR group but had no effect in Stat1 mutated 3′UTR group (Additional file 10: Fig. S7B). We also transfected BMDMs with miR-139-3p mimic or inhibitor. miR-139-3p mimic decreased the protein expression of total Stat1 compared with control or miR-139-3p inhibitor group (Fig. 5I, J; Additional file 1: Table S10). Besides, miR-139-3p mimic significantly attenuated LPS-induced elevation of Stat1 phosphorylation with expression of iNOS decreased while expression of Arg1 increased in BMDMs (Fig. 5K, L; Additional file 1: Table S11). These data suggested that miR-139-3p promotes M2 macrophage polarization by suppressing the expression and activation of Stat1 in macrophages.

### miR-139-3p in MSC^ATV^-EV confers cardioprotection in AMI rats

**Fig. 6:**
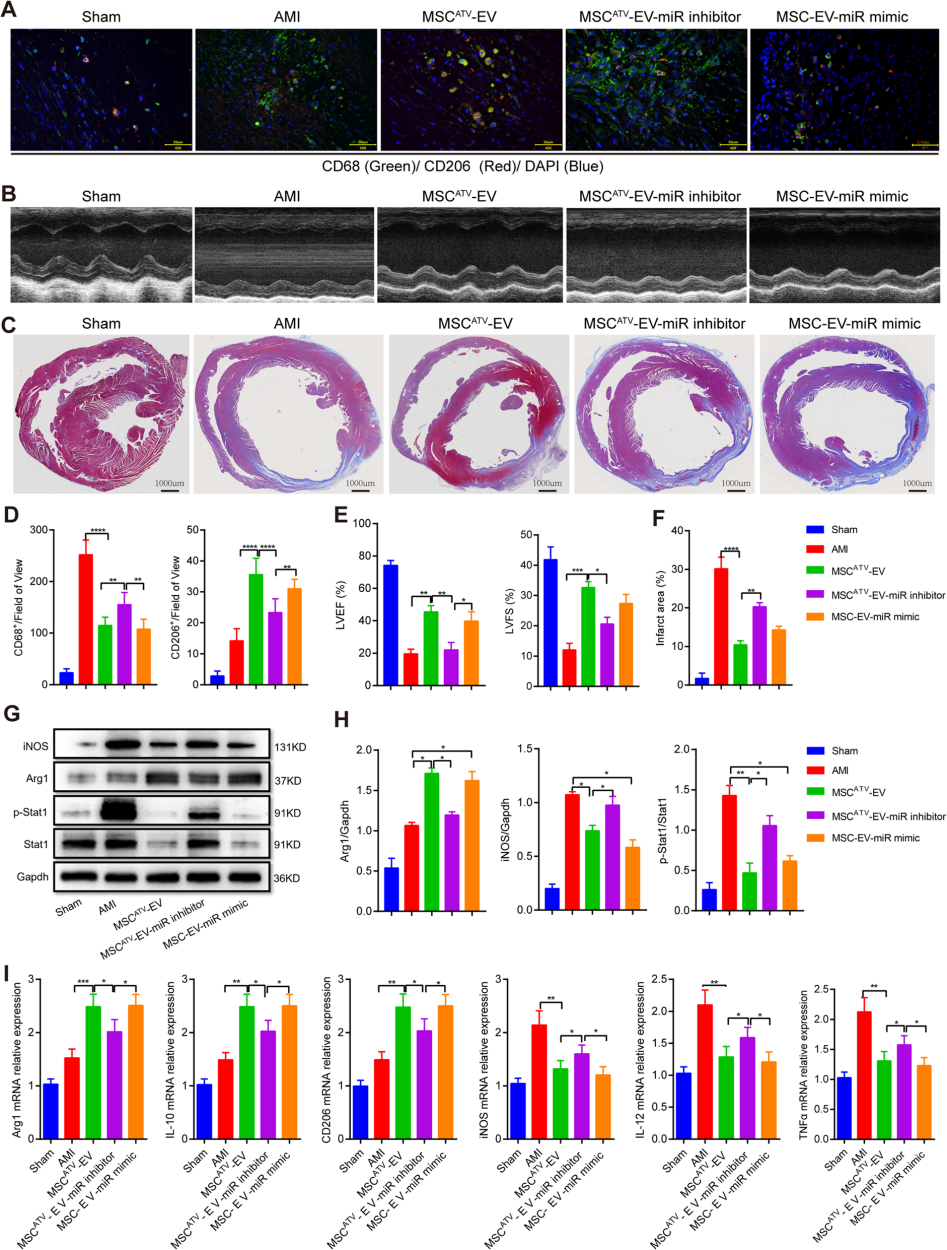
MSC^ATV^-EV transfer of miR-139-3p enhances M2 macrophage polarization in infarcted region of AMI rats. **A** Representative confocal images of CD68^+^ (M1) and CD206^+^ (M2) macrophages within the infarct zone on day 3 after AMI. **B** Quantification of CD68^+^ or CD206^+^ macrophages in A, *n* = 6–7 per group. **C** Representative M-mode echocardiogram of each group on day 28 after AMI. **D** Comparisons of LVEF and LVFS in each group based on the echocardiographic images, *n* = 6–7 per group. *P < 0.05, **P < 0.01, ***P < 0.001. **E** Representatives cross sections of AMI heart with Masson trichrome staining on day 28. **F** Quantification of infarct size of each group in **E**, *n* = 6–7 per group. **G** Western blot analysis of iNOS, Arg1, and p-Stat1 expressions in infarct myocardium of each group. **H** Quantification of iNOS, Arg1, and p-Stat1 expressions in **G**, *n* = 3 per group. **I** The mRNA expressions of macrophage M1 (iNOS, IL-12, TNF-α) and M2 (Arg1, IL-10, CD206) markers in infarcted region measured by qPCR, *n* = 3-4 per group. *P < 0.05, **P < 0.01, ***P < 0.001, ****P < 0.0001. MSC^ATV^-EV-miR inhibitor, MSC^ATV^-EV with miR-139-3p inhibitor; MSC-EV-miR mimic, MSC-EV with miR-139-3p mimic. Arg1, arginase 1; AMI, acute myocardial infarction; ATV, atorvastatin; DAPI, 4',6-diamidino-2-phenylindole; EV, extracellular vesicle; IL, interleukin; iNOS, inducible nitric oxide synthase; LVEF, left ventricular ejection fraction; LVFS, left ventricular fractional shortening; MSCs, mesenchymal stem cells; p-Stat1, phosphorylated signal transducer and activator of transcription 1; qPCR, quantitative polymerase chain reaction; Stat1, signal transducer and activator of transcription 1; TNF-α, tumor necrosis factor alpha

To validate whether EVmiR-139-3p mediates the cardioprotective effects of MSC^ATV^-EV in vivo, we transfected MSC^ATV^-EV with miR-139-3p inhibitor (MSC^ATV^-EV-miR inhibitor) and MSC-EV with miR-139-3p mimic (MSC-EV-miR mimic), then injected them into infarct border zone of myocardium 30 min after AMI in rats. As shown in Fig. 6 and Additional file 1: Table S12, on day 3 post-AMI, MSC^ATV^-EV-miR inhibitor significantly increased the total macrophage marker of CD68^+^ and decreased the M2 marker of CD206^+^ in the border zone compared with both groups of MSC^ATV^-EV and MSC-EV-miR mimic (*P* < 0.01–0.0001) (Fig. 6A, D). On day 28 after AMI, MSC^ATV^-EV-miR inhibitor significantly reduced the cardiac function of LVEF compared with both groups of MSC^ATV^-EV and MSC-EV-miR mimic (*P* < 0.01 and 0.05) (Fig. 6B, E), while significantly increased the infarct size compared with MSC^ATV^-EV group (20.27 ± 2.78% vs. 10.41 ± 2.76%, *P* < 0.01) but not significantly with MSC-EV-miR mimic group (20.27 ± 2.78% vs. 14.24 ± 2.55%, *P* = no significance) (Fig. 6C, F). Furthermore, MSC^ATV^-EV as well as MSC-EV-miR mimic both significantly upregulated the protein expression of Arg1 (both *P* < 0.05) but downregulated that of iNOS and phosphorylated Stat1 in comparison with AMI group (all *P* < 0.01–0.05). In contrast, knocking down miR-139-3p in MSC^ATV^-EV significantly decreased the protein expression of Arg1 (*P* < 0.05) but increased that of iNOS and phosphorylated Stat1 in the infarcted region (all *P* < 0.05) in comparison with MSC^ATV^-EV (Fig. 6G, H). Similarly, and consistently, miR-139-3p inhibitor significantly impaired the superior effects of MSC^ATV^-EV on promoting the mRNA expressions of the macrophage M2 markers of Arg1, IL-10, and CD206 and on suppressing those M1 markers of iNOS, IL-12, or TNF-α in infarcted region (all *P* < 0.05), while miR-139-3p mimic significantly enhanced the efficacy of MSC-EV reaching a similar level of MSC^ATV^-EV (Fig. 6I).

**Fig. 7:**
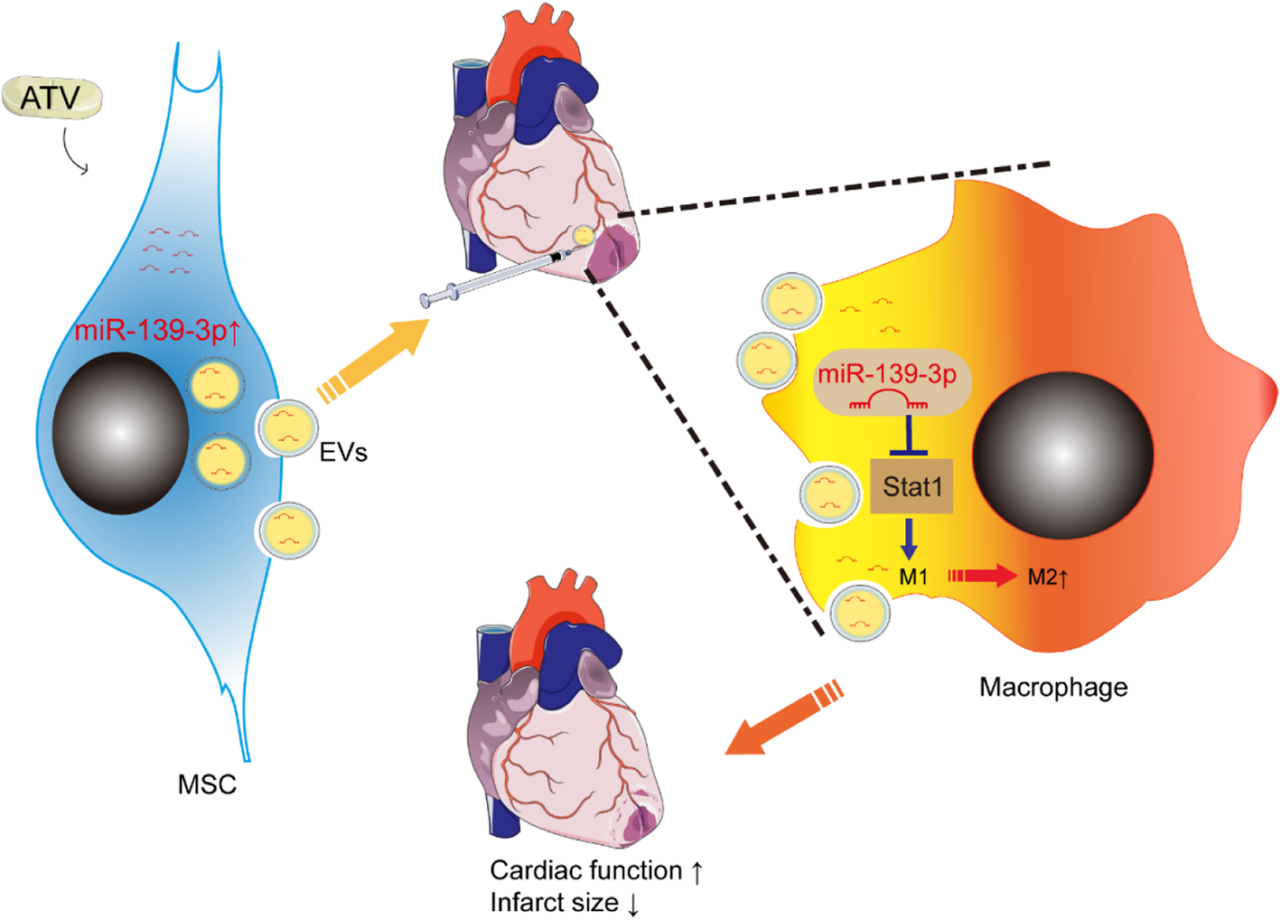
Schematic summary of our main findings. Intramyocardial injection of rat bone marrow MSC^ATV^-EV in border zone delivers the upregulated miR-139-3p into infiltrated macrophages and promotes M2 macrophage polarization by suppressing Stat1 expression and activation, facilitating cardiac repair after infarction. ATV, atorvastatin; EVs, extracellular vesicles; MSCs, mesenchymal stem cells; Stat1, signal transducer and activator of transcription 1

Taken together, all these results above suggested that MSC^ATV^-EV delivered the upregulated miR-139-3p into infarcted myocardium to prevent from macrophage infiltration and inhibit Stat1 activation in infiltrated macrophages, shifting macrophage polarization from M1 to M2, which alleviates inflammation and enhances cardiac repair after AMI (Fig. 7).

## Discussion

The main findings in the present study are as follows: (1) intramyocardial injection of MSC^ATV^-EV was found to remarkably decrease CD68^+^ total macrophage infiltration in the infarcted region with the number of CD206^+^ (M2) macrophages increased and to markedly improve heart function with infarct size reduced; (2) MSC^ATV^-EV was shown to shift macrophage polarization from M1 to M2 in vitro; (3) upregulated miR-139-3p in MSC^ATV^-EV was revealed to be delivered into macrophages to promote M2 polarization via inhibiting Stat1 expression and activation; (4) knockdown of miR-139-3p in MSC^ATV^-EV was verified to eliminate its macrophage M2 polarization shifting and cardioprotective effects in AMI rats. This study was the first to reveal the miRNA profiles of MSC^ATV^-EV and find miR-139-3p is a cardioprotective mediator of promoting macrophage M2 polarization from M1 by targeting Stat1, providing new insights into the mechanisms of MSC^ATV^-EV in superior effects on cardiac repair for AMI.

Shifting macrophage polarization from M1 to M2 is a promising way to promote myocardial repair after AMI [45–48]. In the first 3 days after AMI, proinflammatory M1 macrophages dominate in the infarct region to remove cell debris and degrade extracellular matrix by secreting cytokines, chemokines, growth factors, and matrix metalloproteinases [30], whereas the persistent M1 macrophages can expand inflammation to damage nonischemic myocardium and hinder myocardial repair [49]. On days 5 to 7 post-AMI, anti-inflammatory M2 macrophages become dominant and produce restorative factors such as IL-10, vascular endothelial growth factor, and transforming growth factor β1 (TGF-β1) to promote cardiac repair [30, 31, 35]. Previous studies have reported that EVs secreted by cardiosphere-derived cells or MSCs (untreated) promoted M2 macrophage polarization in a rat or mouse myocardial ischemia-reperfusion model, with LVEF values of 10 to 12 percentage points increase [17, 50]. Another study showed that LPS-pretreated MSCs facilitate M2 macrophage polarization in a mouse AMI model, however without the data of cardiac function [51]. Our findings in the present study indicate that MSC^ATV^-EV can not only restrict total macrophage infiltration in the infarct region of acute stage AMI but also effectively shift its macrophages from M1 to M2 polarization, resulting in prominent improvements of cardiac function with as high as by 31.2% and even 23.6% increase in LVEF value compared with both AMI and MSC-EV groups (52.89% vs. 21.66% and 29.31%) with infarct size reduced by 19.8% and 7.9% (10.22% vs. 30.06% and 18.12%) respectively. Most importantly, pretreatment of MSCs with ATV is the safest and the most acceptable, feasible and straightforward strategy in clinical translation so far.

Mechanistically, stem cell-derived EVs deliver various molecules that affect macrophage polarization, such as EV miR-181b from cardiosphere-derived cells and miR-182 from MSC-derived EVs [17, 50]. We previously explored the long ncRNA profiles in MSC^ATV^-EV and proved that ATV pretreatment makes MSC-derived EVs rich of H19, which promotes angiogenesis and reduces cardiomyocyte apoptosis in the infarcted rat hearts [28]. However, the miRNA profiles of MSC^ATV^-EV had not been revealed before. In this study, we discovered that ATV pretreatment induced miR-139-3p secretion from MSCs and EVs produced from ATV-pretreated MSCs transported miR-139-3p into macrophages to enhance M2 polarization. Although we did not explore how ATV induces miR-139-3p secretion from MSCs, previous studies have reported that statins inhibit enhancer of zeste homolog 2 expression, which is known to downregulate miR-139 [52–56]. Additionally, Papangeli, I. et al. has also reported that ATV can increase miR-139-5p expression in human umbilical vein endothelial cells [57]. Hence, it is a logical mechanism by which ATV pretreatment enhances secretion of EV miR-139-3p from MSCs.

miR-139(-3p/-5p) is a well-known tumor suppressor and a promising but not well-studied cardiac protector [58–60]. It was downregulated in several cardiovascular diseases. For example, the expression of miR-139-3p was reported to be decreased in the peripheral blood of patients suffering from the first AMI (GSE24591); the expression of miR-139-5p also decreased in the ischemic left ventricular myocardium during heart-lung transplantation, in the myocardium of patients died within 7 days after AMI, and in the coronary artery tissues of patients with coronary atherosclerosis [56, 61, 62]; the expression of miR-139 decreased as well in hearts of patients with primary dilated cardiomyopathy [63]. However, all of these studies did not further explore how miR-139(-3p/-5p) influences those cardiovascular diseases. The present study explored the mechanisms of superior efficacy of MSC^ATV^-EVs for cardiac repair after AMI and revealed for the first time that upregulated miR-139-3p in MSC^ATV^-EVs was delivered into macrophages to shift macrophage polarization from M1 to M2 via suppressing the expression and activation of Stat1. We preliminarily validated that miR-139-3p was a potential cardiac reparative factor for AMI as many blood monocyte-derived macrophages were infiltrated in the infarct region during the early stage of AMI.

Stat1 activation is well known to promote M1 macrophage polarization and its suppression facilitates M2 polarization [44, 64]. One study recently reported that miR-139-5p inhibited apoptosis of human arterial smooth muscle cells in the process of atherosclerosis via downregulating Stat1 [56]. Another study reported miR-139/Stat3 axis was involved in osteoclastogenesis in lung adenocarcinoma microenvironment [65]. We for the first time found in this study that miR-139-3p specifically inhibited the expression and activation of Stat1 to promote M2 macrophage polarization for cardiac repair by MSC^ATV^-EV delivery after AMI. In addition, miR-139-3p/-5p may also affect macrophage functions of other aspects such as macrophage migration and secretion of inflammatory factors [66, 67]. We also found miR-139-3p mimics in MSC-EV as well as miR-139-3p in MSC^ATV^-EV both reduced the total macrophage infiltration in the infarct region on day 3 after AMI, while knocking down miR-139-3p in MSC^ATV^-EV reversed the beneficial effects, further verifying for the first time miR-139-3p to be able to restrict excessive inflammation in the acute stage of infarcted hearts. As far as we know, this is the first study to prove the key roles of miR-139-3p/Stat1 pathway in macrophage polarization shifting and excessive infiltration restricting in the infarct region post-AMI.

### Clinical implications and limitations

Given the above findings, MSC^ATV^-EV is expected to have great potential for clinical translation in the treatment of AMI patients, since MSC pretreatment with ATV, a commonly used clinical medication, is the safest and the most acceptable, feasible, and straightforward modification, particularly favoring the clinical translation for AMI treatment [51, 68].

Despite these promising results, there remain some limitations in the present study. First, given the pleiotropic effect of miRNA, it is possible that miR-139-3p targets multiple genes and affects various cell functions (Additional file 1: Table S13). Here, we investigated only the polarization effects of EV miR-139-3p on macrophages not including other cardiac cells, such as cardiomyocytes and fibroblasts, and we did not explore other possible target genes of miR-139-3p that might regulate macrophage polarization. Secondly, other differentially expressed miRNAs in MSC^ATV^-EV may also exert a role in cardioprotection. Finally, it is unclear whether the potent cardiac repair effects of MSC^ATV^-EV in experimental study could be observed in patients and whether intravenous administration of MSC^ATV^-EV works. These are worth to be further investigated in the future.

## Conclusions

The present study demonstrated that intramyocardial injection of MSC^ATV^-EV in the infarct region could not only restricted macrophage infiltration but also delivered EV miR-139-3p to macrophages, which further inhibits the expression and activation of Stat1 to promote M2 macrophage polarization, remarkably facilitating cardiac repair after AMI. This study provides new insights into the mechanisms for the superior effects of MSC^ATV^-EV to MSC-EV in cardiac repair of rat AMI, which has the great potential for clinical translation.

## Supplementary Information

**Additional file 1.** Supplementary data.

**Additional file 2.** The ARRIVE guidelines 2.0: author checklist.

**Additional file 3: Figure S1.** The cell viability of MSC or MSC^ATV^ after 72h treatment measured by MTT assay. ns: no significance.

**Additional file 4: Supplementary Video.** Dynamical observation of PKH26-labeled MSC^ATV^-EV uptake by BMDMs using Opera Phenix high-content screening system.

**Additional file 5: Figure S2.** Molecule size ranges of RNA samples from MSC-EV and MSC^ATV^-EV. RNA molecule size ranges of four MSC-EV (A) and four MSC^ATV^-EV (B) were established by Agilent 2200 TapeStation Instrument. HSRNA ladders include 25, 200, 500, 1,000, 2,000, 4,000 and 6,000 nt.

**Additional file 6: Figure S3.** Non-coding RNA annotation of MSC-EV and MSC^ATV^-EV. The mapped read counts were plotted on a log-scale. “Others” refer to the reads that cannot be mapped to the known non-coding RNA reads.

**Additional file 7: Figure S4.** The original image of Fig. 4F (Interaction network between target genes of three upregulated miRNAs (miR-139-3p, miR-320-3p, miR-501-3p) and upregulated genes in patients suffered from first acute myocardial infarction (GEO: GSE24591).)

**Additional file 8: Figure S5.** The original image of Fig. 4G (Interaction network between target genes of three upregulated miRNAs (miR-139-3p, miR-320-3p, miR-501-3p) and downregulated genes in human monocyte-derived M2 macrophages (GEO: GSE32164)).

**Additional file 9: Figure S6.** Consensus sequence of rno-miR-139. Stacked histogram displays the counts of mapped reads to rno-miR-139 in miRNA-seq of MSC-EV (red) and MSC^ATV^-EV (blue). Seqlogos of mapped reads were displayed above the histogram while reference sequence of rno-mir-139 marked with the locations of rno-miR-139-5p and rno-miR-139-3p were displayed below the histogram.

**Additional file 10: Figure S7.** Stat1 is the target gene of miR-139-3p. A, Consequential pairing of Stat1 3’UTR and miR-139-3p predicted by TargetScan. B, Luciferase activity was measured and normalized by Renilla luciferase activity. WT, wild type. Mut, mutated. NC, negative control. ^**^P<0.01 compared with WT 3’UTR+NC mimic group, *n* = 4 per group.

**Additional file 11.** Images of the original blots.

## Data Availability

The data that support the findings of this study are available from the corresponding author upon reasonable request.

## Abbreviations

AMI: Acute myocardial infarction
Arg1: Arginase 1
ATV: Atorvastatin
BMDMs: Bone marrow-derived macrophages
CSF: Colony stimulating factor
DMSO: Dimethyl sulfoxide
EVs: Extracellular vesicles
FBS: Fetal bovine serum
GEO: Gene expression omnibus
GO: Gene ontology
IL: Interleukin
IMDM: Iscove’s Modified Dulbecco’s Medium
iNOS: Inducible nitric oxide synthase
KEGG: Kyoto Encyclopedia of Genes and Genomes
LPS: Lipopolysaccharide
LVEDd: Left ventricular end-diastolic dimension
LVEDV: Left ventricular end-diastolic volume
LVEF: Left ventricular ejection fraction
LVESd: Left ventricular end-systolic dimension
LVESV: Left ventricular end-systolic volume
LVFS: Left ventricular fractional shortening
miRNAs: microRNAs
MSC^ATV^: Atorvastatin-pretreated mesenchymal stem cells
MSC^ATV^-EV: Extracellular vesicles derived from mesenchymal stem cells pretreated with atorvastatin
MSC-EV: Extracellular vesicles derived from mesenchymal stem cells
MSCs: Mesenchymal stem cells
NC: Negative control
ncRNA: Non-coding RNA
PBS: Phosphate buffer saline
qPCR: Quantitative polymerase chain reaction
RPM: Reads per million
SD: Standard deviation
sEVs: Small extracellular vesicles
Stat1: Signal transducer and activator of transcription 1
TNF-α: Tumor necrosis factor alpha
