# Supplementary material for: Atorvastatin-pretreated mesenchymal stem cell-derived extracellular vesicles promote cardiac repair after myocardial infarction via shifting macrophage polarization by targeting microRNA-139-3p/Stat1 pathway
Source: BMC Med. 2023 Mar 16;21:96. doi: 10.1186/s12916-023-02778-x (PMC10022054; doi:10.1186/s12916-023-02778-x)
Supplement: Supplementary file 1 — Additional file 1. Supplementary data. [file 12916_2023_2778_MOESM1_ESM.docx]

# Supplementary data

# Supplementary Methods

**Flow cytometry analysis for MSC phenotype**

MSC phenotype was characterized by FACS analysis. MSCs were incubated with fluorescent antibodies for CD45 (Invitrogen), CD11b/c (Miltenyi Biotec), CD29 (Invitrogen), and CD90 (Invitrogen). Isotype-matched antibodies were also used to recognize non-specific staining. The expression of these markers was analyzed by flow cytometry (Accuri C6, BD Biosciences).

**MTT assay for cell viability**

Cell viability was measured by MTT assay as described before.(36) Briefly, MSCs were plated in 96-well plates and treated with 1 μmol/L ATV or equal volumes of DMSO in IMDM containing 10% FBS for 24 hours. Then cells were washed with PBS three times and cultured with fresh FBS-free IMDM for another 48 hours. The optical density (OD) values of MTT were measured at 492 nm using a Microplate Reader (Thermo Scientific). The larger OD value represents the better cell viability.

**Transmission electron microscopy**

5 μL EVs were diluted in 45 μL cold PBS and added to an electron microscopy grid (Electron Microscopy Sciences, PA, USA). After incubation at room temperature for 5 min, the EVs were negatively stained with 0.75% uranyl acetate dihydrate for another 5 min. Then the samples were washed with PBS three times and excess liquid was removed. The grids were examined in transmission electron microscope to observe the morphology of EVs.

**EV Labeling and uptake**

For EV uptake experiments, rat BMDMs were labeled with PKH26 Red Fluorescent Cell Linker Kit (Sigma-Aldrich) or PKH67 Green Fluorescent Cell Linker Kit (Sigma-Aldrich) according to the manufacturer’s protocol. The labeled EVs were then washed at 120,000g ultracentrifugation for twice to remove excess dye. For dynamical observation, BMDMs were plated into clear-bottom, black-walled 96-well plates and cultured with medium containing PKH26-labeled EVs in the Opera Phenix high-content screening system (PerkinElmer). Images were dynamically captured using a 40× air/water objective and processed using Harmony High Content Imaging and Analysis Software (PerkinElmer) to visualize the EV uptake by macrophages.

For immunofluorescence cytochemistry, PKH67-labeled EVs were added into BMDMs in vitro. After 2- or 12-hour incubation, the macrophages were washed and fixed with 4% paraformaldehyde. Then the cells were stained with phalloidine and DAPI at room temperature. Immunofluorescence images were captured and analyzed.

**Rat model of myocardial infarction**

Six- to eight-week-old male Sprague-Dawley rats (200-220g weight) were anesthetized by intraperitoneal injection of 100 mg/kg ketamine combined with 10 mg/kg xylazine and ventilated via tracheal intubations connected to a rodent ventilator. We calculated the animal number following our published results (28), which measured recovery from MI in these model systems. At a 2-sided significance of 0.05 and a power of 0.90, we estimated that six to nine rats in each group will be required. Therefore, we allocated seven rats to each group in each experiment and the total number of rats used was 126. Animals were completely randomized into different groups using a random-number table. All surgeries and data analyses were performed in a blinded fashion. The corresponding author was aware of the group allocation at the different stages of the experiment.(23,28,29) As previously described, MI surgery was performed by ligating left anterior descending coronary artery with an 6-0 silk suture by an experienced and professional operator of Fuwai Hospital Experimental Animal Center. MI induction was verified by loss of color in the region below the ligation area. The Sham group underwent the same surgical procedures except for the permanent ligation step. Various EVs (105 μg, in 100 μL PBS) or an equivalent volume of PBS were injected at three different points around the border zone of infarction 30 minutes after left anterior descending ligation using a 31-gauge Hamilton syringe. There were no exclusions.

**Immunofluorescence Staining**

To detect macrophage distribution in myocardium, the paraffin-embedded heart tissue sections were dewaxed, rehydrated, and then permeabilized with 0.1% TritonX-100 in PBS for ten minutes. Tissue sections were blocked in 5% bovine serum albumin for one hour at room temperature. Primary antibodies were diluted at 1:1000 and incubated overnight at 4℃, including anti-CD68 (Abcam) and anti-CD206 (Abcam). Nuclei were stained with DAPI to allow automated cell segmentation and quantification. Then the diluted fluorophore-conjugated secondary antibodies (1:500) were incubated. Images of sections were analyzed and captured on a Zeiss LSM 780 confocal microscope. For quantification of positively stained cells, three sections per heart and five fields per section were counted per animal. The quality control of the cell segmentation was performed by a pathologist who was blinded to the study design.

**Quantitative polymerase chain reaction (****qPCR)**

Total RNA was extracted from rat hearts, macrophage cells, and EV pellets by TRIzol reagent (Invitrogen, Life Technology, USA) following the manufacturer’s instructions. Specially, during the process of EV RNA extraction, the synthetic miRNA caenorhabditis elegans miR-39-3p (cel-miR-39-3p; RiboBio, Guangzhou, China) was added to act as external control. cDNA from mRNA was synthesized using Hifair® Ⅱ 1st Strand cDNA Synthesis SuperMix for qPCR (YEASEN, Shanghai, China) and then mixed with Hieff ® qPCR SYBR Green Master Mix (YEASEN, Shanghai, China) and forward or reverse primers to run qPCR process. For miRNA quantitation, miDETECT A Track^TM^ miRNA qPCR Starter Kit (RiboBio, Guangzhou, China) was used for cDNA synthesis and qPCR process. All the qPCR tests were performed on an Applied Biosystems QuantStudio 3 (Foster City, CA, USA). The sequences of designed primers were: Arg1, forward GTGGCGTTGACCTTGTCTTG, reverse GCCTGGTTCTGTTCGGTTTG; IL-10, forward TTGAACCACCCGGCATCTAC, reverse CCAAGGAGTTGCTCCCGTTA; Mrc1, forward TCAACTCTTGGACTCACGGC, reverse ATGATCTGCGACTCCGACAC; iNOS, forward GGCTTGGGTCTTGTTAGCCT, reverse GAAGAGAAACTTCCAGGGGCA; IL-12, forward CATGGCTGGTGCACAGAAAC, reverse ATGCTCGTCCACATGTCACC; TNF-α, forward CATCCGTTCTCTACCCAGCC, reverse AATTCTGAGCCCGGAGTTGG; Gapdh, forward AACTCCCATTCTTCCACCTTTG, reverse CTCTTGCTCTCAGTATCCTTGC; the primer sequences of miR-139-3p、miR-320-3p、miR-501-3p、miR-200c-3p、miR-205、miR-340-3p、let-7f-5p、let-7a-1-3p、let-7c-2-3p、let-7f-5p、cel-miR-39-3p、U6 were designed and synthesized by Guangzhou Ribobio biotechnology company. The relative gene expressions were calculated using the 2^-ΔΔCt^ method with β-actin as the reference gene for mRNA, U6 snRNA for miRNA extracted from cells or tissues, and cel-miR-39-3p for miRNA extracted from EVs.

**Western Blot**

Proteins of heart tissues, total cells or EVs were extracted using RIPA lysis buffer (Thermo Fisher Scientific, MA, USA). The concentrations of tissue or cell proteins were quantified by BCA Protein Assay Kit (Beyotime, Shanghai, China). The concentrations of EV proteins were tested by Micro BCA Protein Assay Kit (Thermo Fisher Scientific, MA, USA). Proteins (10-30 μg) were separated by 8% ~ 10% SDS-polyacrylamide gel electrophoresis and then transferred into polyvinylidene difluoride (PVDF) membranes. The membranes were blocked with 5% non-fat dry milk in 1×TBST at room temperature for one hour and then incubated with anti-Arg1, anti-iNOS, anti-Alix, anti-TSG101, anti-CD81, anti-CD63, and anti-Gapdh at 4°C overnight, which was followed by the incubation of secondary horse radish peroxidase-conjugated antibody (CST) at room temperature for 1 hour. The proteins were finally visualized by SuperSignal West Femto Maximum Sensitivity Substrate (Thermo Scientific) and detected by ChemiDoc™ MP Imaging System (Bio-Rad, USA). The gray values of protein bands were tested by ImageJ software.

**Collection of human sequencing data**

We retrieved three published human gene expression data from the NIH/NCBI gene expression omnibus (GEO) online database (https://www.ncbi.nlm.nih.gov/geo/). To be specific, series GSE24591 is a gene and miRNA expression profiling of patients affected by first AMI; GSE32164 is a gene expression profiling of human monocyte-derived macrophage polarization; GSE123342 is a dataset of peripheral blood whole transcriptome in patients with acute myocardial infarction and at follow-up.

Differential gene or miRNA expression were analyzed using GEO 2R function between AMI group and normal group in series GSE24591, between M2 macrophages and resting fully differentiated macrophages in series GSE32164, or between stable coronary artery disease group and AMI D0 group in series GSE123342.

**Luciferase reporter assay**

1-2×10^4^ 293T cells were seeded in 96-well plate. When the cells reached 50-80% confluence, miR-139-3p or NC mimics and Stat1 wild type (WT) or mutated (Mut) 3’UTR reporter plasmids were transfected into 293T cells following the manufacturer’s protocol (RiboBio, China). After 24-48 hours incubation, firefly luciferase and Renilla luciferase activites were detected using Dual-Glo luciferase assay system (Promega). The relative luciferase activity is calculated by the ratio of Renilla luciferase activity to firefly luciferase activity.

# Supplementary Tables

## Table S1. Original data in Figure 2.

| Parameters | Time | Sham | AMI | MSC-EV | MSC^ATV^-EV |
| --- | --- | --- | --- | --- | --- |
| LVEF (%; n=6-7) | 0 d | 73.86±4.06 | 72.13±2.77 | 74.01±3.66 | 72.85±3.55 |
|  | 3 d | 71.75±3.12 | 50.64±4.11 | 49.56±4.24 | 57.82±2.31 |
|  | 7 d | 73.49±3.07 | 38.93±3.19 | 40.38±5.14 | 48.23±2.49 |
|  | 28 d | 76.67±3.48 | 21.66±1.54 | 29.31±2.64 | 52.89±5.40^****/####^ |
| LVFS (%; n=6-7) | 0 d | 41.71±3.89 | 41.40±4.76 | 43.79±3.27 | 42.77±5.19 |
|  | 3 d | 41.67±3.36 | 28.39±2.79 | 29.80±3.86 | 30.30±3.20 |
|  | 7 d | 39.93±3.58 | 25.16±3.16 | 26.47±3.64 | 32.31±3.84 |
|  | 28 d | 45.42±3.51 | 17.95±2.33 | 24.86±2.10 | 37.70±2.73^****/####^ |
| LVESV (μL; n=6-7) | 0 d | 79.07±4.57 | 76.66±4.57 | 81.66±6.75 | 82.86±6.06 |
|  | 3 d | 80.72±5.48 | 115.3±6.12 | 106.4±7.14 | 113.2±12.03 |
|  | 7 d | 82.59±4.67 | 196.5±10.68 | 206 ±11.11 | 134 ±12.19 |
|  | 28 d | 94.32±4.63 | 463.4±19.02 | 400 ±18.89 | 261.30±11.23^****/####^ |
| LVEDV (μL; n=6-7) | 0 d | 238.3±19.12 | 231.6±13.21 | 246.9±24.85 | 244.4±23.81 |
|  | 3 d | 248.5±17.41 | 238.7±18.65 | 253.5±22.09 | 189.1±19.57 |
|  | 7 d | 245.6±14.96 | 366.5±14.98 | 350.1±27.02 | 246.3±26.91 |
|  | 28 d | 315.0±13.45 | 542.9±22.04 | 493.6±26.79 | 397.5±17.95^****/####^ |
| Infarct area (%; n=6-7) | 28 d | 1.00±1.16 | 30.06±6.84 | 18.12±4.64 | 10.22±2.82^****/#^ |
| CD68^+^ macrophages/ field of view (n=6-7) | 3 d | 12.57±4.50 | 242±21.82 | 177.6±27.57 | 116.9±14.5^****/####^ |
| CD206^+^ macrophages/ field of view (n=6-7) | 3 d | 2.57±1.51 | 13.43±4.50 | 23.14±4.45 | 31±3.61^****/##^ |
| Arg1 protein expression (n=3) | 3 d | 0.35±0.08 | 0.60±0.09 | 1.06±0.04 | 1.26±0.02^*/#^ |
| iNOS protein expression (n=3) | 3 d | 0.07±0.005 | 0.69±0.04 | 0.50±0.02 | 0.25±0.004^**/##^ |
| Arg1 mRNA expression (n=3-4) | 3 d | 1.01±0.10 | 1.42±0.14 | 1.80±0.17^*^ | 2.39±0.17^##^ |
| IL-10 mRNA expression (n=3-4) | 3 d | 1.05±0.12 | 1.41±0.16 | 1.79±0.13^*^ | 2.40±0.16^#^ |
| CD206 mRNA expression (n=3-4) | 3 d | 0.99±0.11 | 1.39±0.14 | 1.82±0.15^**^ | 2.40±0.18^#^ |
| iNOS mRNA expression (n=3-4) | 3 d | 1.02±0.10 | 2.54±0.19 | 1.82±0.14^***^ | 1.42±0.13^##^ |
| IL-12 mRNA expression (n=3-4) | 3 d | 1.05±0.09 | 2.51±0.14 | 1.82±0.14^***^ | 1.38±0.13^##^ |
| TNF-α mRNA expression (n=3-4) | 3 d | 1.01±0.13 | 2.52±0.13 | 1.81±0.15^***^ | 1.40±0.15^##^ |

^*^P<0.05, ^**^P<0.01, ^***^P<0.001, ^****^P<0.0001 compared with AMI group. ^#^P<0.05, ^##^P<0.01, ^###^P<0.001, ^####^P<0.0001 compared with MSC-EV group.

## Table S2. Original data in Figure 3.

| Parameters | Control | M1 | M2 | LPS+MSC-EV | LPS+MSC^ATV^-EV |
| --- | --- | --- | --- | --- | --- |
| Arg1 protein expression (n=3) | 0.66±0.01 | 1.14±0.01 | 1.68±0.02^****^ | 1.48±0.02^##^ | 1.66±0.01^###/$$^ |
| iNOS protein expression (n=3) | 0.17±0.002 | 0.71±0.01^****^ | 0.19±0.001 | 0.74±0.004 | 0.53±0.01^###/$$$$^ |
| Arg1 mRNA expression (n=4) | 1.08±0.10 | 1.33±0.13 | 2.08±0.23^****^ | 1.61±0.13 | 2.19±0.24^####/$$^ |
| IL-10 mRNA expression (n=4) | 1.04±0.11 | 1.32±0.13 | 2.09±0.24^****^ | 1.56±0.13 | 2.19±0.22^####/$$$^ |
| CD206 mRNA expression (n=4) | 1.05±0.11 | 1.27±0.16 | 2.11±0.27^****^ | 1.61±0.16 | 2.22±0.27^####/$$^ |
| iNOS mRNA expression (n=4) | 1.03±0.10 | 2.28±0.20^****^ | 1.09±0.10 | 1.75±0.14^###^ | 1.40±0.14^####/$^ |
| IL-12 mRNA expression (n=4) | 1.03±0.11 | 2.28±0.20^****^ | 1.06±0.10 | 1.74±0.13^###^ | 1.42±0.13^####/$^ |
| TNF-α mRNA expression (n=4) | 1.03±0.09 | 2.29±0.20^****^ | 1.09±0.11 | 1.73±0.12^###^ | 1.41±0.14^####/$^ |

^*^P<0.05, ^**^P<0.01, ^***^P<0.001 compared with control group.

^#^P<0.05, ^##^P<0.01, ^####^P<0.0001 compared with M1 group.

^$^P<0.05, ^$$^P<0.01, ^$$$^P<0.001, ^$$$$^P<0.0001 compared with LPS+MSC-EV group.

## Table S3. Original data in Additional file 6: Fig. S3.

| Non-coding RNA | Read counts in  MSC-EV | Read counts in  MSC^ATV^-EV |
| --- | --- | --- |
| tRNA | 5639272.75±263049.23 | 4431003.00±469787.10 |
| piRNA | 4345011.50±162033.74 | 4587690.75±400084.67 |
| rRNA | 1044777.50±216549.00 | 1637957.00±440470.29 |
| Others | 111857.25±23463.07 | 149523.25±20233.33 |
| Y_RNA_etc | 57680.50±13171.21 | 43940.50±21710.58 |
| miRNA | 28894.25±6091.26 | 26995.25±7587.34 |
| snRNA | 9185.75±5062.49 | 9184.00±3988.15 |
| snoRNA | 773.75±114.27 | 896.25±71.11 |

## Table S4. Original data in Figure 4.

| miRNA | log2  (Foldchange) | P-value | Relative miRNA expression in MSC-EV | Relative miRNA expression in  MSC^ATV^-EV |
| --- | --- | --- | --- | --- |
| rno-miR-200c-3p | -4.56 | 0.0005 | 0.98±0.11 | 0.32±0.07^***^ |
| rno-miR-205 | -4.83 | 0.0005 | 1.04±0.14 | 0.54±0.07^***^ |
| rno-miR-501-3p | 1.84 | 0.009 | 1.04±0.12 | 1.77±0.21^***^ |
| rno-miR-139-3p | 3.93 | 0.02 | 1.04±0.11 | 1.29±0.16^*^ |
| rno-miR-340-3p | -4.17 | 0.02 | 1.03±0.11 | 0.45±0.10^***^ |
| rno-let-7f-5p | -1.28 | 0.02 | 1.04±0.11 | 0.74±0.09^**^ |
| rno-let-7a-1-3p | -1.56 | 0.03 | 1.01±0.11 | 0.71±0.09^**^ |
| rno-let-7c-2-3p | -1.56 | 0.03 | 1.09±0.16 | 0.65±0.11^**^ |
| rno-miR-320-3p | 1.25 | 0.04 | 1.06±0.12 | 1.58±0.21^**^ |

^*^P<0.05, ^**^P<0.01, ^***^P<0.001 compared with MSC-EV group.

## Table S5. Twenty-five down-regulated miRNAs in patients with first acute myocardial infarction compared with healthy people from GSE24591 dataset in GEO.

| miRNA-ID | Adjusted P-value | P-value | Log (Foldchange) | Organism |
| --- | --- | --- | --- | --- |
| hsa-miR-1 | 0.04 | 0.008 | -1.73 | Homo sapiens |
| hsa-miR-32 | 0.005 | 0.00003 | -1.72 | Homo sapiens |
| hsa-miR-362-3p | 0.01 | 0.0003 | -1.51 | Homo sapiens |
| hsa-miR-545 | 0.005 | 0.00004 | -1.50 | Homo sapiens |
| hsa-miR-29b | 0.01 | 0.0001 | -1.46 | Homo sapiens |
| hsa-miR-219-5p | 0.005 | 0.00005 | -1.34 | Homo sapiens |
| hsa-miR-768-5p | 0.03 | 0.01 | -1.32 | Homo sapiens |
| hsa-miR-33a | 0.01 | 0.0002 | -1.34 | Homo sapiens |
| hsa-let-7d* | 0.02 | 0.003 | -1.33 | Homo sapiens |
| hsa-miR-136 | 0.05 | 0.01 | -1.26 | Homo sapiens |
| hsa-miR-133b | 0.11 | 0.04 | -1.26 | Homo sapiens |
| hsa-miR-590-5p | 0.01 | 0.0003 | -1.20 | Homo sapiens |
| hsa-miR-101 | 0.01 | 0.0002 | -1.19 | Homo sapiens |
| hsa-miR-9* | 0.02 | 0.002 | -1.18 | Homo sapiens |
| hsa-miR-335* | 0.02 | 0.001 | -1.18 | Homo sapiens |
| hsa-miR-33b | 0.01 | 0.0002 | -1.17 | Homo sapiens |
| hsa-miR-598 | 0.01 | 0.0003 | -1.14 | Homo sapiens |
| hsa-miR-424 | 0.08905 | 0.0288476 | -1.12012 | Homo sapiens |
| hsa-miR-142-3p | 0.00648 | 0.0002573 | -1.07909 | Homo sapiens |
| hsa-miR-24-1* | 0.00648 | 0.0003195 | -1.06975 | Homo sapiens |
| hsa-miR-19a | 0.01037 | 0.0006572 | -1.06383 | Homo sapiens |
| hsa-miR-548a-5p | 0.00644 | 0.0001133 | -1.04541 | Homo sapiens |
| hsa-miR-17* | 0.00648 | 0.0002057 | -1.04049 | Homo sapiens |
| hsa-miR-150 | 0.01598 | 0.0015756 | -1.01451 | Homo sapiens |
| hsa-miR-139-3p | 0.01789 | 0.0023943 | -1.01128 | Homo sapiens |

*Low expression.

## Table S6. Part of differentially expressed genes in patients with acute myocardial infarction compared with those with stable coronary artery disease from GSE123342 dataset in GEO.

|  | Adjusted  P-value | P-value | Log (Foldchange) | Organism |
| --- | --- | --- | --- | --- |
| MAP3K2 | 3.31E-07 | 4.70E-12 | -1.01 | Homo sapiens |
| SLC16A3 | 4.58E-07 | 1.30E-11 | -4.94E-01 | Homo sapiens |
| ETF1 | 6.15E-07 | 3.99E-11 | -3.37E-01 | Homo sapiens |
| FRMD4B | 6.15E-07 | 4.48E-11 | -4.18E-01 | Homo sapiens |
| SERINC3 | 6.15E-07 | 5.23E-11 | -5.33E-01 | Homo sapiens |
| PCYT1A | 2.74E-06 | 2.72E-10 | -4.18E-01 | Homo sapiens |
| RIT1 | 3.25E-06 | 3.83E-10 | -6.92E-01 | Homo sapiens |
| USP3 | 3.25E-06 | 4.30E-10 | -5.47E-01 | Homo sapiens |
| RNF146 | 5.23E-06 | 8.15E-10 | -3.65E-01 | Homo sapiens |
| RNF144B | 5.24E-06 | 9.14E-10 | -1.29 | Homo sapiens |
| CACUL1 | 5.24E-06 | 9.66E-10 | -6.13E-01 | Homo sapiens |
| ATP6AP1 | 5.59E-06 | 1.11E-09 | -4.30E-01 | Homo sapiens |
| CHST11 | 5.66E-06 | 1.20E-09 | -5.32E-01 | Homo sapiens |
| STK17B | 6.48E-06 | 1.47E-09 | -7.05E-01 | Homo sapiens |
| AP1G1 | 6.55E-06 | 1.62E-09 | -3.85E-01 | Homo sapiens |
| ZFAS1 | 6.55E-06 | 1.67E-09 | -8.35E-01 | Homo sapiens |
| PSEN1 | 9.50E-06 | 2.68E-09 | -5.92E-01 | Homo sapiens |
| microRNA 139 | 3.29E-01 | 9.56E-02 | 1.29E-01 | Homo sapiens |
| GABBR1 | 1.32E-04 | 4.01E-07 | 3.35E-01 | Homo sapiens |

## Table S7. Original data in Figure 5B-D.

| Measurement | Control | LPS+  MSC^ATV^-EV | LPS+MSC^ATV^-EV-NC inhibitor | LPS+MSC^ATV^-EV-miR inhibitor | LPS+MSC-EV-NC mimic | LPS+MSC-EV-miR mimic |
| --- | --- | --- | --- | --- | --- | --- |
| Arg1 protein expression (n=3) | 0.52±0.005 | 1.80±0.01 | 1.68±0.01 | 1.28±0.01^**^ | 1.23±0.02 | 1.89±0.02^####^ |
| iNOS protein expression (n=3) | 0.19±0.001 | 0.66±0.008 | 0.64±0.005 | 1.09±0.01^****^ | 1.20±0.004 | 0.82±0.007^####^ |
| Arg1 mRNA expression (n=3-4) | 1.01±0.10 | 1.81±0.15 | 1.90±0.15 | 1.45±0.15^**^ | 1.51±0.15 | 2.04±0.15^#^ |
| IL-10 mRNA expression (n=3-4) | 1.00±0.10 | 1.80±0.15 | 1.90±0.15 | 1.45±0.15^*^ | 1.51±0.15 | 2.02±0.15^#^ |
| CD206 mRNA expression (n=3-4) | 1.00±0.10 | 1.80±0.15 | 1.90±0.15 | 1.45±0.15^*^ | 1.51±0.15 | 2.02±0.15^#^ |
| iNOS mRNA expression (n=3-4) | 1.01±0.11 | 1.20±0.15 | 1.21±0.15 | 1.61±0.15^*^ | 1.60±0.15 | 1.22±0.15^#^ |
| IL-12 mRNA expression (n=3-4) | 1.04±0.11 | 1.21±0.15 | 1.21±0.15 | 1.60±0.15^*^ | 1.61±0.15 | 1.22±0.15^#^ |
| TNF-α mRNA expression (n=3-4) | 1.00±0.11 | 1.21±0.15 | 1.21±0.15 | 1.61±0.15^**^ | 1.61±0.15 | 1.22±0.15^#^ |

^*^ P<0.05, ^**^P<0.01, ^***^P<0.001, ^****^P<0.0001 compared with LPS+ MSC^ATV^-EV-NC inhibitor group. ^#^P<0.05, ^####^P<0.0001 compared with LPS+MSC-EV-NC mimic group.

## Table S8. Original data in Figure 5F-H.

| Measurement | Control | M1 | LPS+NC mimic | LPS+miR-139-3p mimic |
| --- | --- | --- | --- | --- |
| Arg1 protein expression (n=3) | 1.31±0.02 | 1.61±0.01 | 1.75±0.004 | 2.19±0.04^**/##^ |
| iNOS protein expression (n=3) | 0.24±0.002 | 1.02±0.01 | 1.16±0.002 | 0.68±0.01^****/####^ |
| Arg1 mRNA expression (n=3-4) | 1.00±0.10 | 1.25±0.15 | 1.45±0.15 | 2.18±0.20^***/##^ |
| IL-10 mRNA expression (n=3-4) | 1.01±0.10 | 1.25±0.15 | 1.48±0.16 | 2.18±0.23^*/#^ |
| CD206 mRNA expression (n=3-4) | 1.00±0.10 | 1.28±0.16 | 1.47±0.19 | 2.15±0.21^***/##^ |
| iNOS mRNA expression (n=3-4) | 1.00±0.10 | 2.31±0.23 | 2.51±0.21 | 1.20±0.15^**/##^ |
| IL-12 mRNA expression (n=3-4) | 1.00±0.10 | 2.31±0.21 | 2.52±0.21 | 1.20±0.15^***/##^ |
| TNF-α mRNA expression (n=3-4) | 1.00±0.10 | 2.31±0.21 | 2.52±0.21 | 1.21±0.15^***/##^ |

^*^ P<0.05, ^**^P<0.01, ^***^P<0.001, ^****^P<0.0001 compared with M1 group. ^#^P<0.05, ^##^P<0.01, ^####^P<0.0001 compared with LPS+NC mimic group.

## Table S9. Original data in Additional file 10: Fig. S7.

| Measurement | WT 3’UTR+  NC mimic | WT 3’UTR+  miR-139-3p mimic | Mut 3’UTR+  NC mimic | Mut 3’UTR+  miR-139-3p mimic |
| --- | --- | --- | --- | --- |
| Relative luciferase activity | 1.04±0.13 | 0.62±0.10^**^ | 1.01±0.11 | 1.22±0.15 |

WT, wild type. Mut, mutated. NC, negative control. ^**^P<0.01 compared with WT 3’UTR+NC mimic group. n=4 per group.

## Table S10. Original data in Figure 5I-J.

| Measurement | Control | NC mimic | miR-139-3p mimic | NC inhibitor | miR-139-3p inhibitor |
| --- | --- | --- | --- | --- | --- |
| Stat1 protein expression | 1.30±0.01 | 1.02±0.01 | 0.54±0.02^****^ | 1.21±0.004 | 1.18±0.01^###^ |

^****^P<0.0001 compared with NC mimic group. ^###^P<0.001 compared with miR-139-3p mimic group. n=3 per group.

## Table S11. Original data in Figure 5K-L.

| Measurement | Control | M1 | LPS+NC | LPS+miR-139-3p mimic | LPS+miR-139-3p inhibitor |
| --- | --- | --- | --- | --- | --- |
| Arg1 protein expression | 0.75±0.01 | 1.43±0.01 | 1.19±0.01 | 1.65±0.02^****^ | 1.01±0.01^###^ |
| iNOS protein expression | 0.11±0.01 | 0.91±0.01 | 0.89±0.01 | 0.55±0.01^****^ | 0.66±0.01^#^ |
| p-Stat1/Stat1 protein expression | 0.08±0.001 | 1.22±0.005 | 0.99±0.02 | 0.59±0.01^**^ | 1.07±0.01^###^ |

^**^P<0.01, ^****^P<0.0001 compared with LPS+NC group. ^#^P<0.05, ^###^P<0.001 compared with LPS+miR-139-3p mimic group. n=3 per group.

## Table S12. Original data in Figure 6.

| Parameters | Sham | AMI | MSC^ATV^-EV | MSC^ATV^-EV- miR inhibitor | MSC-EV-miR mimic |
| --- | --- | --- | --- | --- | --- |
| CD68^+^ macrophages/ field of view (n=6-7) | 23.36±7.43 | 251.90±28.52 | 114.40±16.24^****^ | 155.00±23.84^##^ | 107.60±19.25^$$^ |
| CD206^+^ macrophages/ field of view (n=6-7) | 2.86±1.57 | 14.14±3.89 | 35.57±5.29^****^ | 23.29±4.46^####^ | 31.00±3.06^$$^ |
| LVEF (%; n=6-7) | 74.14±8.22 | 19.65±7.60 | 45.40±10.34^**^ | 22.01±11.98^##^ | 39.73±15.50^$^ |
| LVFS (%; n=6-7) | 41.82±11.14 | 12.04±5.68 | 32.62±5.01^***^ | 20.55±5.88^#^ | 27.33±7.94 |
| Infarct area (%; n=6-7) | 1.71±3.68 | 30.14±7.97 | 10.41±2.76^****^ | 20.27±2.78^##^ | 14.24±2.55 |
| Arg1 protein expression (n=3) | 0.54±0.12 | 1.06±0.04 | 1.71±0.07^*^ | 1.19±0.04^#^ | 1.62±0.11^*^ |
| iNOS protein expression (n=3) | 0.20±0.04 | 1.07±0.03 | 0.74±0.05^*^ | 0.98±0.08^#^ | 0.58±0.07^*^ |
| p-Stat1/Stat1 protein expression (n=3) | 0.26±0.09 | 1.43±0.12 | 0.47±0.12^**^ | 1.06±0.13^#^ | 0.62±0.07^*^ |
| Arg1 mRNA expression (n=3-4) | 1.03±0.10 | 1.52±0.16 | 2.49±0.24^***^ | 2.02±0.23^#^ | 2.51±0.21^$^ |
| IL-10 mRNA expression  (n=3-4) | 1.02±0.11 | 1.49±0.14 | 2.49±0.23^**^ | 2.02±0.21^#^ | 2.50±0.21^$^ |
| CD206 mRNA expression (n=3-4) | 1.00±0.11 | 1.49±0.15 | 2.48±0.24^**^ | 2.03±0.22^#^ | 2.50±0.21^$^ |
| iNOS mRNA expression (n=3-4) | 1.04±0.10 | 2.14±0.27 | 1.32±0.15^**^ | 1.60±0.17^#^ | 1.20±0.16^$^ |
| IL-12 mRNA expression (n=3-4) | 1.03±0.10 | 2.10±0.23 | 1.29±0.16^**^ | 1.59±0.16^#^ | 1.21±0.16^$^ |
| TNF-α mRNA expression (n=3-4) | 1.03±0.09 | 2.12±0.24 | 1.31±0.16^**^ | 1.58±0.15^#^ | 1.23±0.13^$^ |

^**^P<0.01, ^***^P<0.001, ^****^P<0.0001 compared with AMI group. ^#^P<0.05, ^##^P<0.01, ^####^P<0.0001 compared with MSC^ATV^-EV group. ^$^P<0.05, ^$$^P<0.01, ^$$$^P<0.001, ^$$$$^P<0.0001 compared with MSC^ATV^-EV-miR inhibitor group.

## Table S13. Other candidate targets of miR-139-3p.

| Gene | miRDB | miRTarBase | miRWalk | TargetScan |
| --- | --- | --- | --- | --- |
| Aak1 | 1 | 0 | 1 | 1 |
| Adam11 | 1 | 0 | 1 | 1 |
| Adprh | 1 | 0 | 1 | 1 |
| Adrb3 | 1 | 0 | 1 | 1 |
| Arih1 | 1 | 0 | 1 | 1 |
| Cdh10 | 1 | 0 | 1 | 1 |
| Cgref1 | 1 | 0 | 1 | 1 |
| Crebl2 | 1 | 0 | 1 | 1 |
| Dcaf10 | 1 | 0 | 1 | 1 |
| Dpy19l3 | 1 | 0 | 1 | 1 |
| Elk1 | 1 | 0 | 1 | 1 |
| Fam163a | 1 | 0 | 1 | 1 |
| Fkbp5 | 1 | 0 | 1 | 1 |
| Foxj3 | 1 | 0 | 1 | 1 |
| Gas7 | 1 | 0 | 1 | 1 |
| Golt1b | 1 | 0 | 1 | 1 |
| Gpr108 | 1 | 0 | 1 | 1 |
| Gpr183 | 1 | 0 | 1 | 1 |
| Hipk1 | 1 | 0 | 1 | 1 |
| Klf7 | 1 | 0 | 1 | 1 |
| Lamtor1 | 1 | 0 | 1 | 1 |
| Map7d1 | 1 | 0 | 1 | 1 |
| Mapk1 | 1 | 0 | 1 | 1 |
| Mcm3 | 1 | 0 | 1 | 1 |
| Mcrs1 | 1 | 0 | 1 | 1 |
| Mgat4a | 1 | 0 | 1 | 1 |
| Miip | 1 | 0 | 1 | 1 |
| Nsun4 | 1 | 0 | 1 | 1 |
| Patl1 | 1 | 0 | 1 | 1 |
| Pgam5 | 1 | 0 | 1 | 1 |
| Prkra | 1 | 0 | 1 | 1 |
| Ptp4a1 | 1 | 0 | 1 | 1 |
| Rab11b | 1 | 0 | 1 | 1 |
| Rab31 | 1 | 0 | 1 | 1 |
| Rasd2 | 1 | 0 | 1 | 1 |
| Rhbdl3 | 1 | 0 | 1 | 1 |
| Scly | 1 | 0 | 1 | 1 |
| Sema4g | 1 | 0 | 1 | 1 |
| Slc39a11 | 1 | 0 | 1 | 1 |
| Sorcs2 | 1 | 0 | 1 | 1 |
| Sp1 | 1 | 0 | 1 | 1 |
| Spry4 | 1 | 0 | 1 | 1 |
| St3gal2 | 1 | 0 | 1 | 1 |
| St8sia3 | 1 | 0 | 1 | 1 |
| Stag2 | 1 | 0 | 1 | 1 |
| Trpc3 | 1 | 0 | 1 | 1 |
| Ube2g1 | 1 | 0 | 1 | 1 |

TargetScan, miRDB, miRTarBase and miRWalk were used to predict targets gene of selected miR-139-3p. 1 represents this gene was predicted as a target gene of miR-139-3p in the corresponding website, while 0 represents not.
