# Supplementary figures and images for: Atorvastatin-pretreated mesenchymal stem cell-derived extracellular vesicles promote cardiac repair after myocardial infarction via shifting macrophage polarization by targeting microRNA-139-3p/Stat1 pathway
Source: BMC Med. 2023 Mar 16;21:96. doi: 10.1186/s12916-023-02778-x (PMC10022054; doi:10.1186/s12916-023-02778-x)

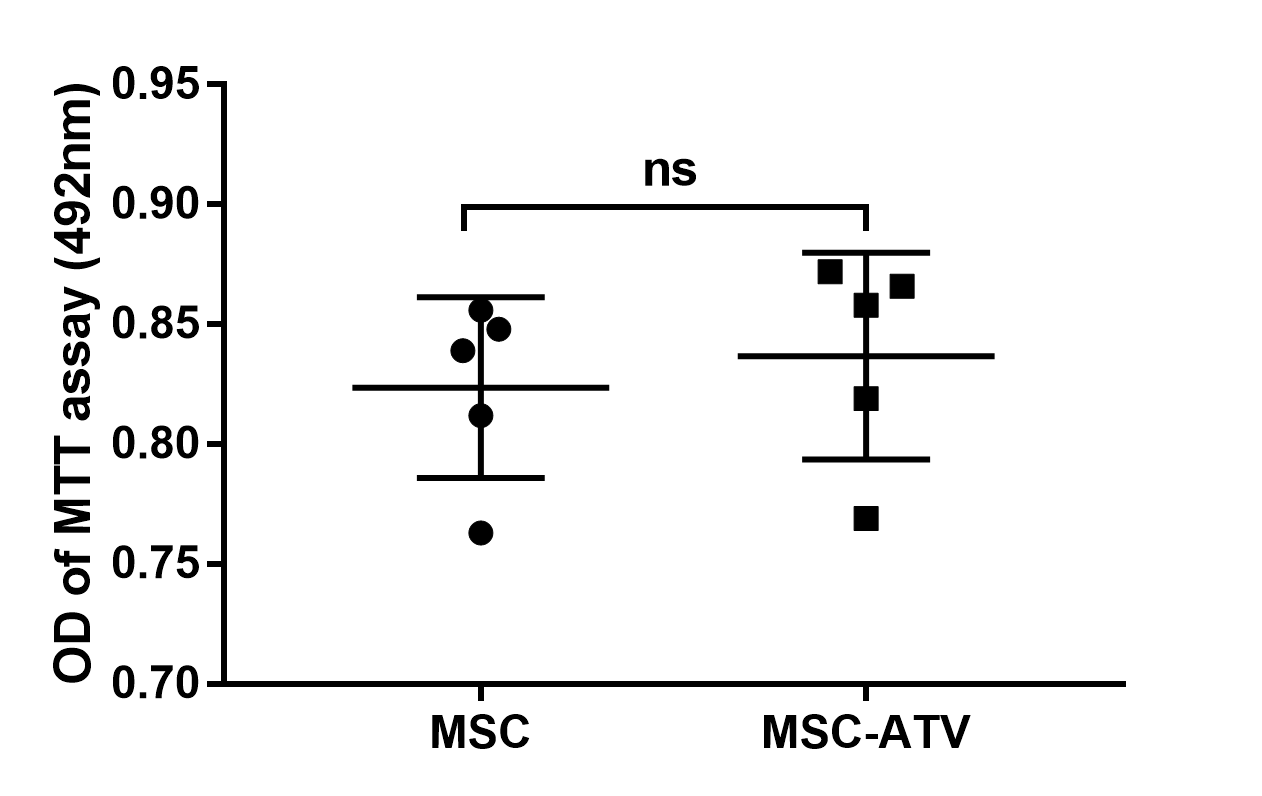

Supplement: Supplementary file 3 — Additional file 3: Figure S1. The cell viability of MSC or MSCATV after 72h treatment measured by MTT assay. ns: no significance. [file 12916_2023_2778_MOESM3_ESM.tif]

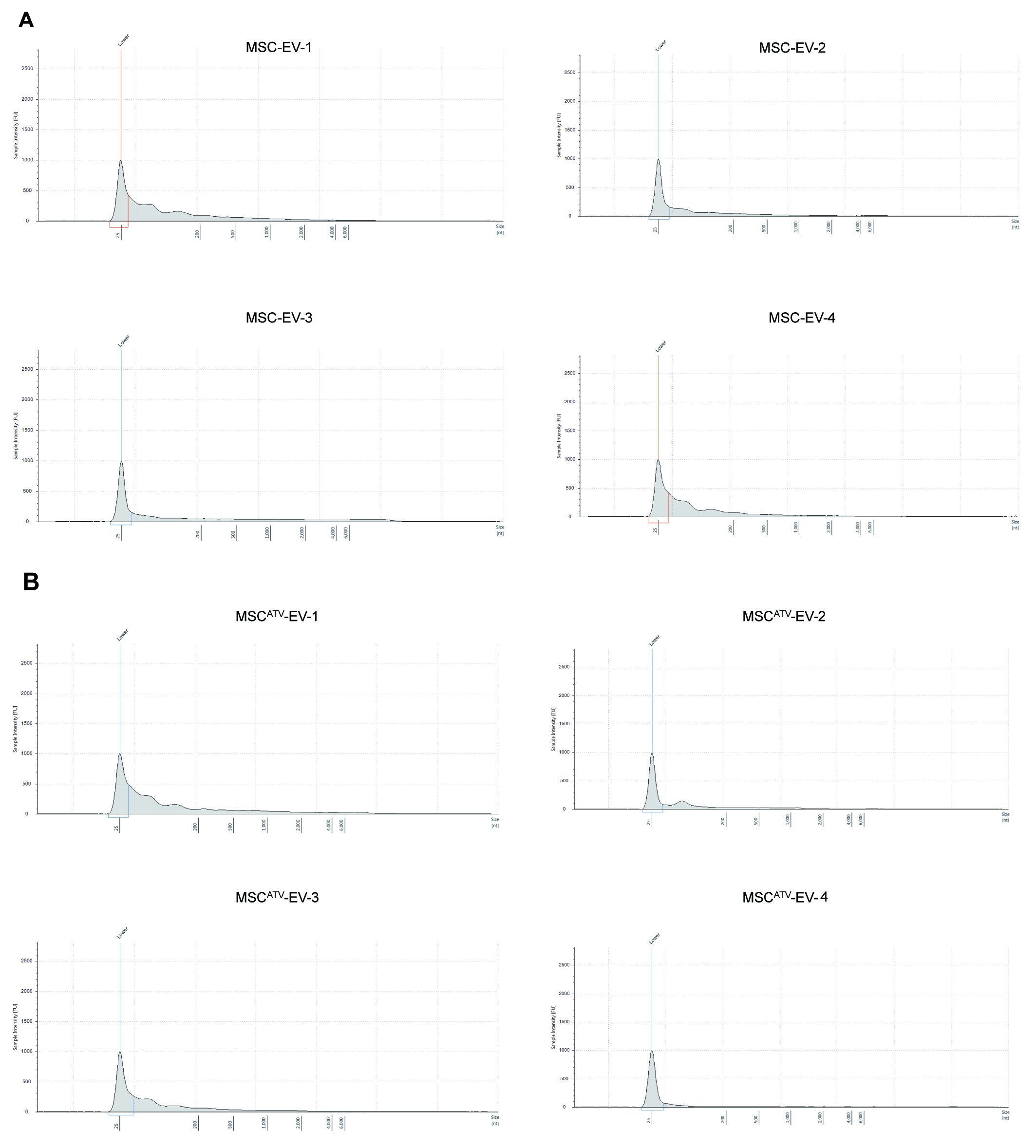

Supplement: Supplementary file 5 — Additional file 5: Figure S2. Molecule size ranges of RNA samples from MSC-EV and MSCATV-EV. RNA molecule size ranges of four MSC-EV (A) and four MSCATV-EV (B) were established by Agilent 2200 TapeStation Instrument. HSRNA ladders include 25, 200, 500, 1,000, 2,000, 4,000 and 6,000 nt. [file 12916_2023_2778_MOESM5_ESM.tif]

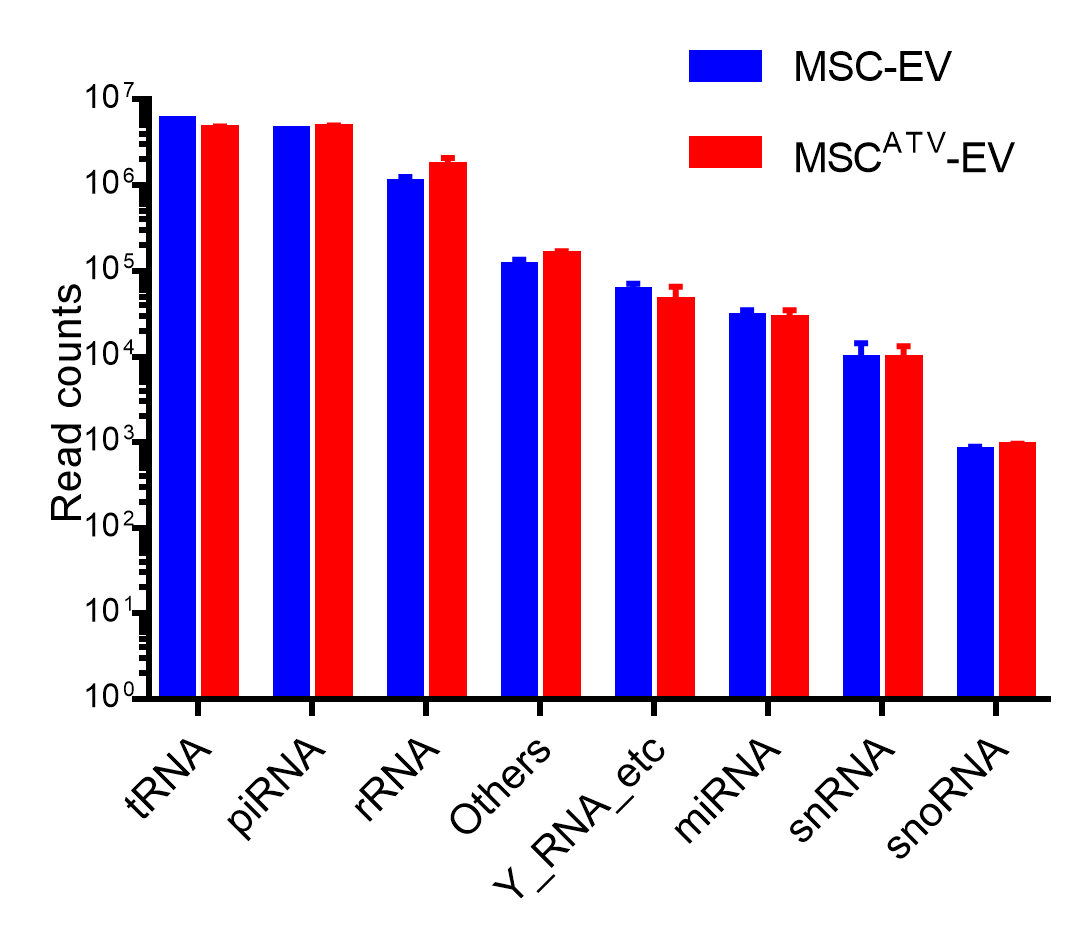

Supplement: Supplementary file 6 — Additional file 6: Figure S3. Non-coding RNA annotation of MSC-EV and MSCATV-EV. The mapped read counts were plotted on a log-scale. “Others” refer to the reads that cannot be mapped to the known non-coding RNA reads. [file 12916_2023_2778_MOESM6_ESM.tif]

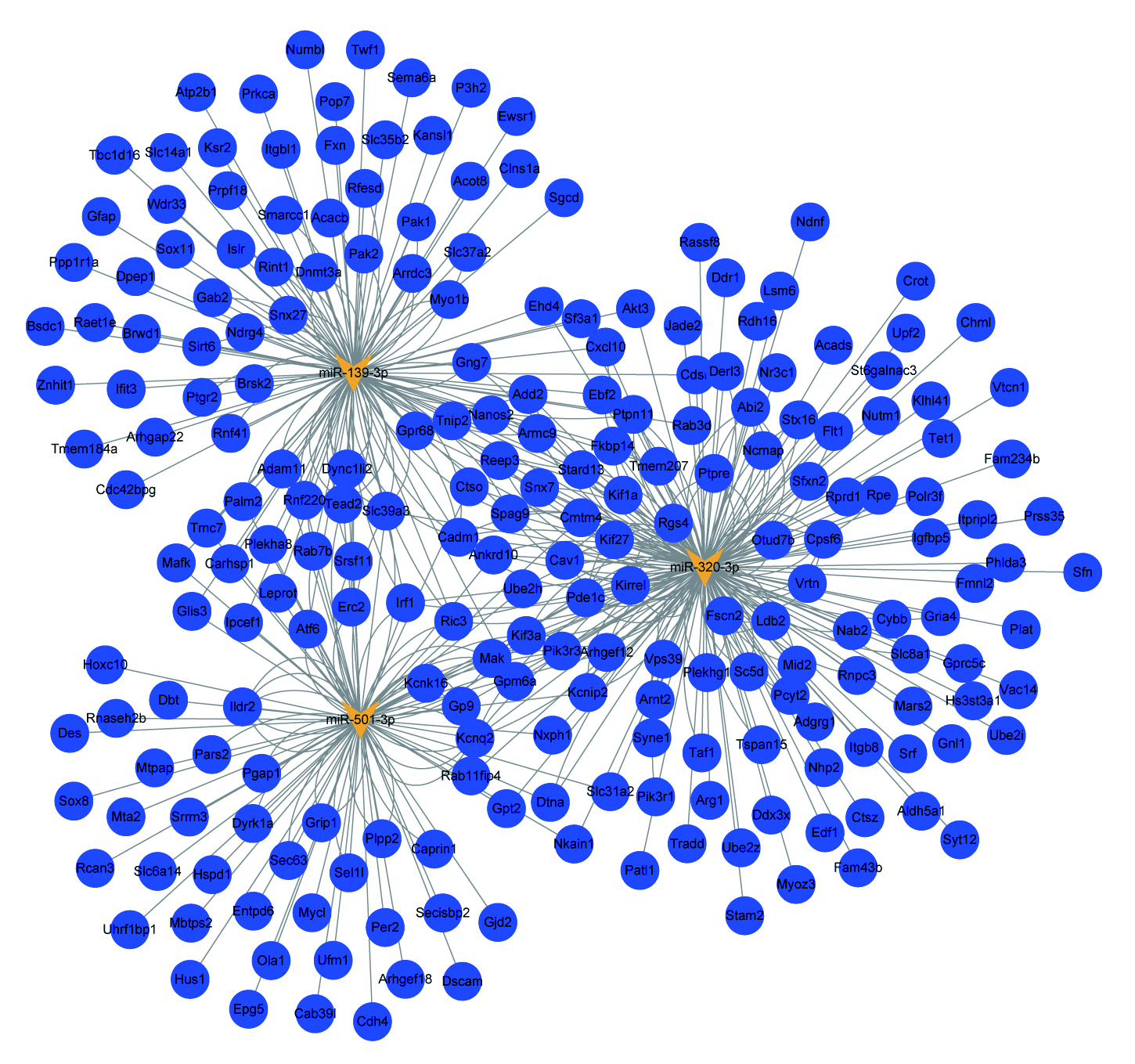

Supplement: Supplementary file 7 — Additional file 7: Figure S4. The original image of Fig. 4F (Interaction network between target genes of three upregulated miRNAs (miR-139-3p, miR-320-3p, miR-501-3p) and upregulated genes in patients suffered from first acute myocardial infarction (GEO: GSE24591).) [file 12916_2023_2778_MOESM7_ESM.tif]

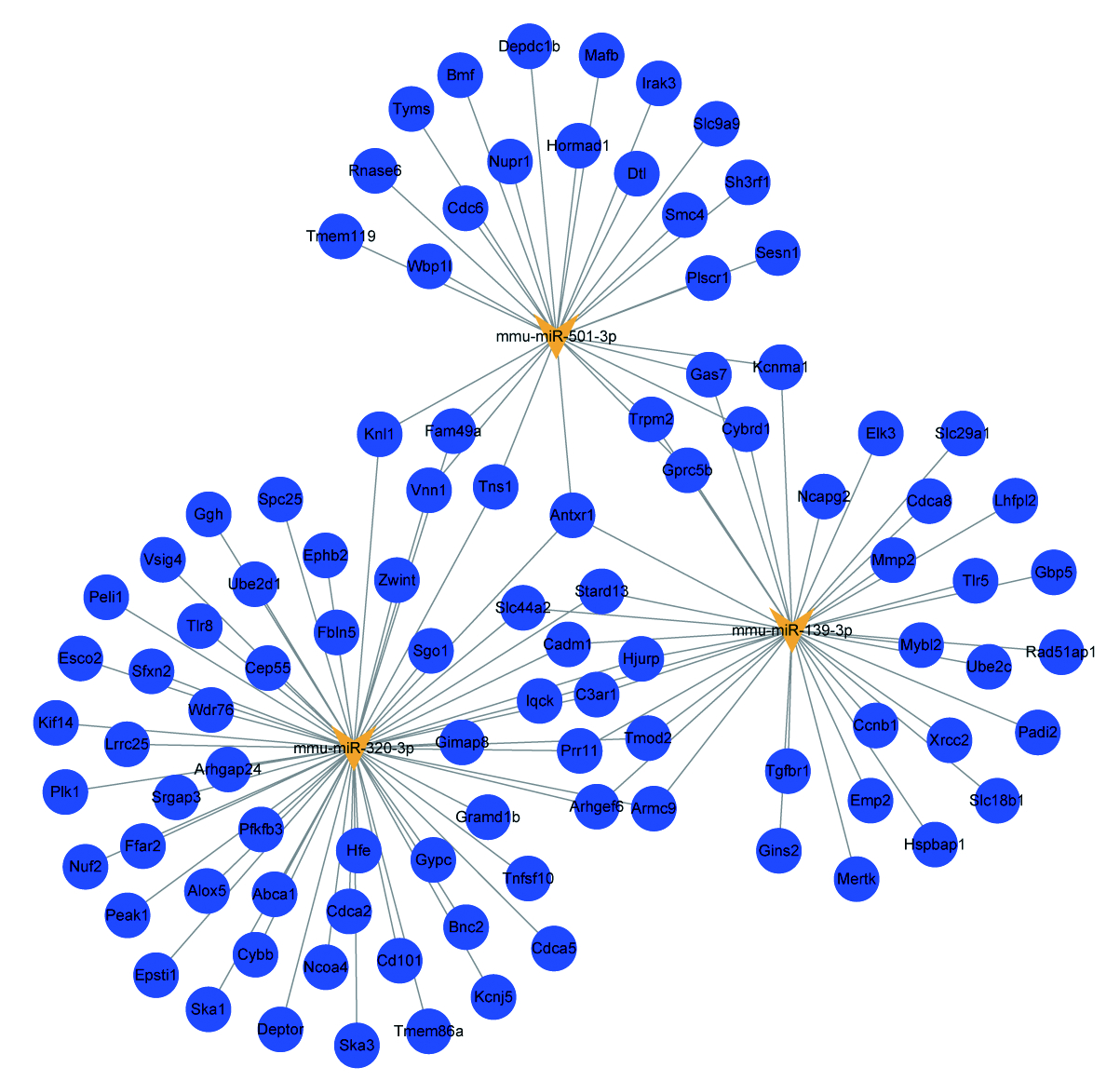

Supplement: Supplementary file 8 — Additional file 8: Figure S5. The original image of Fig. 4G (Interaction network between target genes of three upregulated miRNAs (miR-139-3p, miR-320-3p, miR-501-3p) and downregulated genes in human monocyte-derived M2 macrophages (GEO: GSE32164)). [file 12916_2023_2778_MOESM8_ESM.tif]

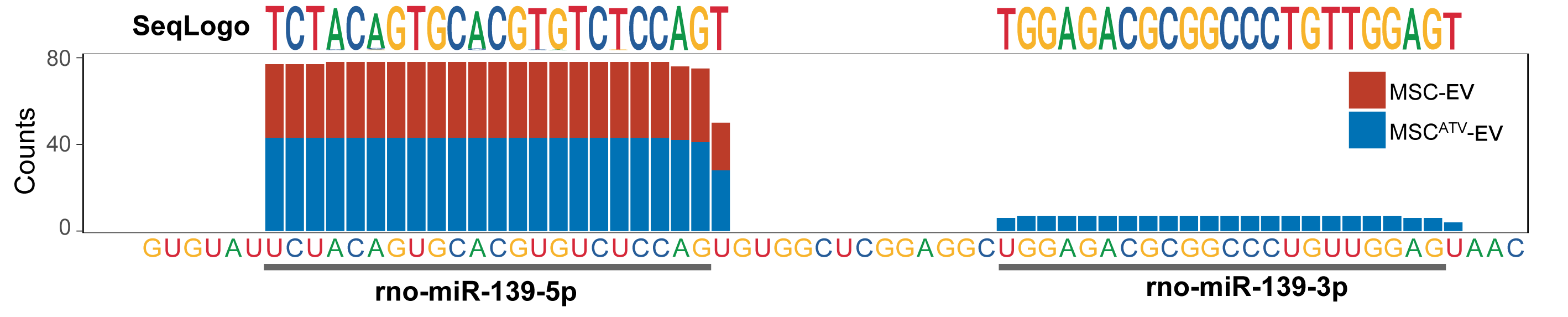

Supplement: Supplementary file 9 — Additional file 9: Figure S6. Consensus sequence of rno-miR-139. Stacked histogram displays the counts of mapped reads to rno-miR-139 in miRNA-seq of MSC-EV (red) and MSCATV-EV (blue). Seqlogos of mapped reads were displayed above the histogram while reference sequence of rno-mir-139 marked with the locations of rno-miR-139-5p and rno-miR-139-3p were displayed below the histogram. [file 12916_2023_2778_MOESM9_ESM.tif]

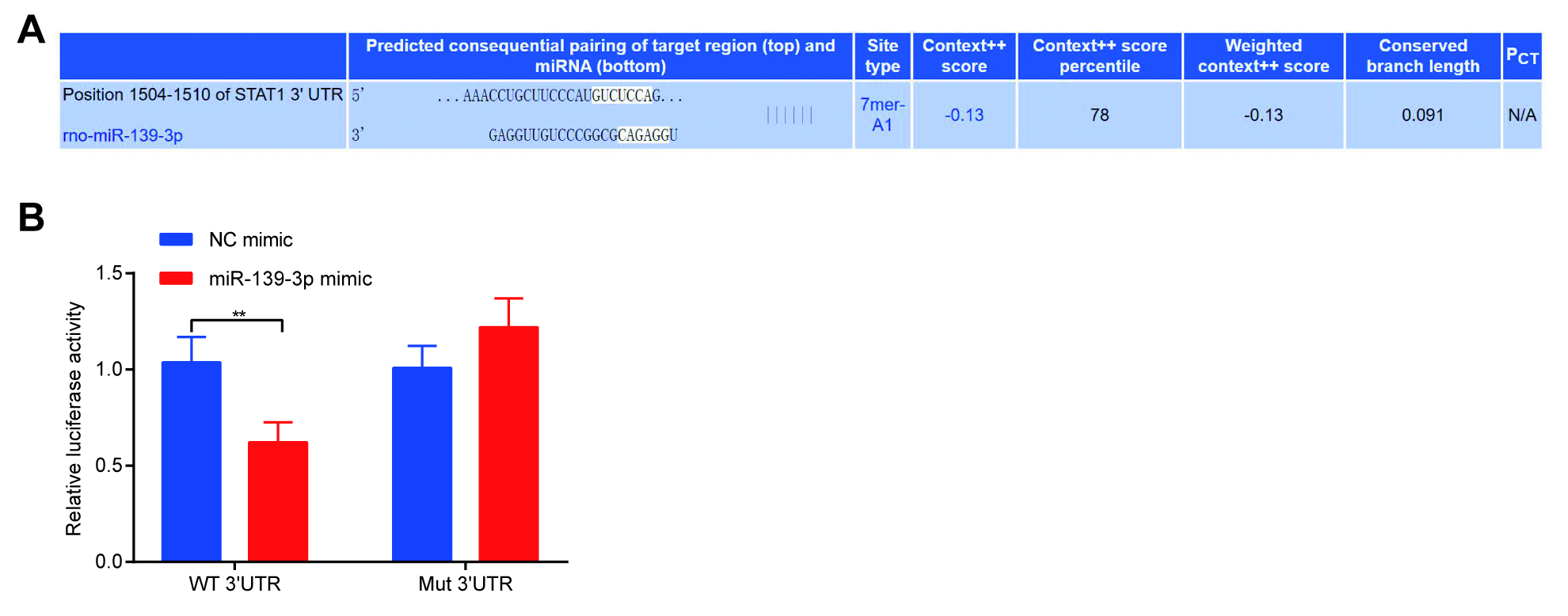

Supplement: Supplementary file 10 — Additional file 10: Figure S7. Stat1 is the target gene of miR-139-3p. A, Consequential pairing of Stat1 3’UTR and miR-139-3p predicted by TargetScan. B, Luciferase activity was measured and normalized by Renilla luciferase activity. WT, wild type. Mut, mutated. NC, negative control. **P<0.01 compared with WT 3’UTR+NC mimic group, n = 4 per group. [file 12916_2023_2778_MOESM10_ESM.tif]
