# Supplementary material for: Atorvastatin-pretreated mesenchymal stem cell-derived extracellular vesicles promote cardiac repair after myocardial infarction via shifting macrophage polarization by targeting microRNA-139-3p/Stat1 pathway
Source: BMC Med. 2023 Mar 16;21:96. doi: 10.1186/s12916-023-02778-x (PMC10022054; doi:10.1186/s12916-023-02778-x)
Supplement: Supplementary file 11 — Additional file 11. Images of the original blots. [file 12916_2023_2778_MOESM11_ESM.docx]

**Additional file 11. Images of the original blots**


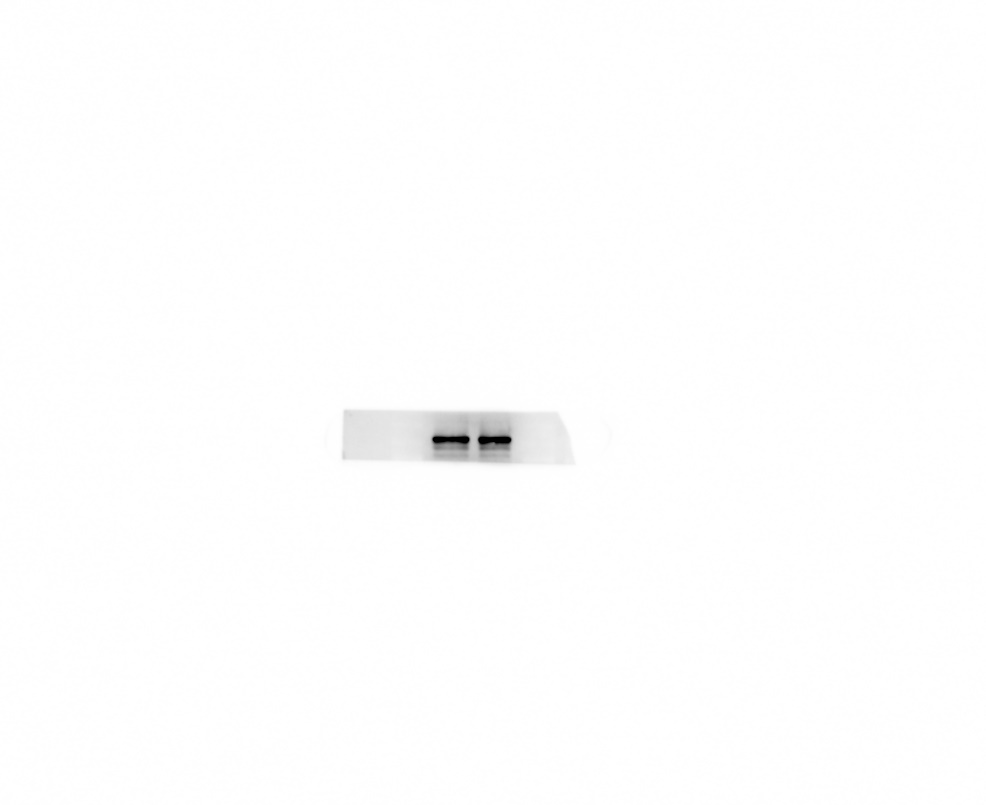


Figure 1E. Alix


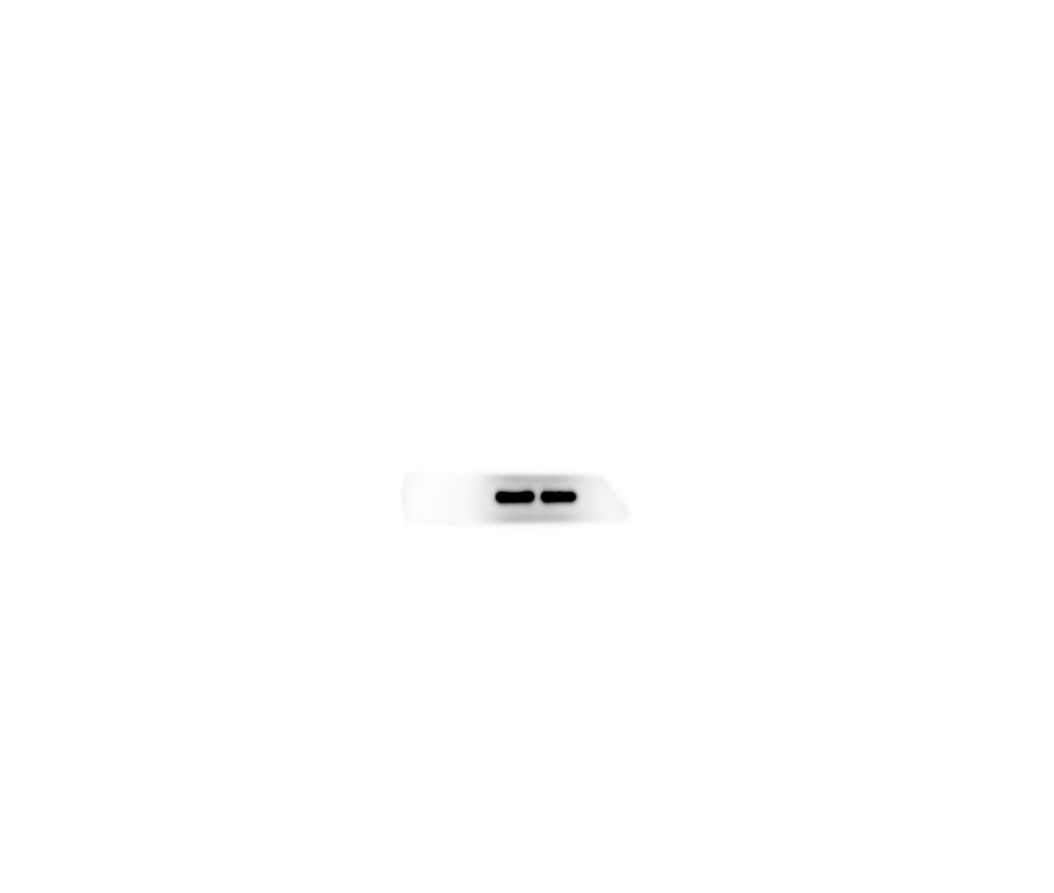


Figure 1E. CD63


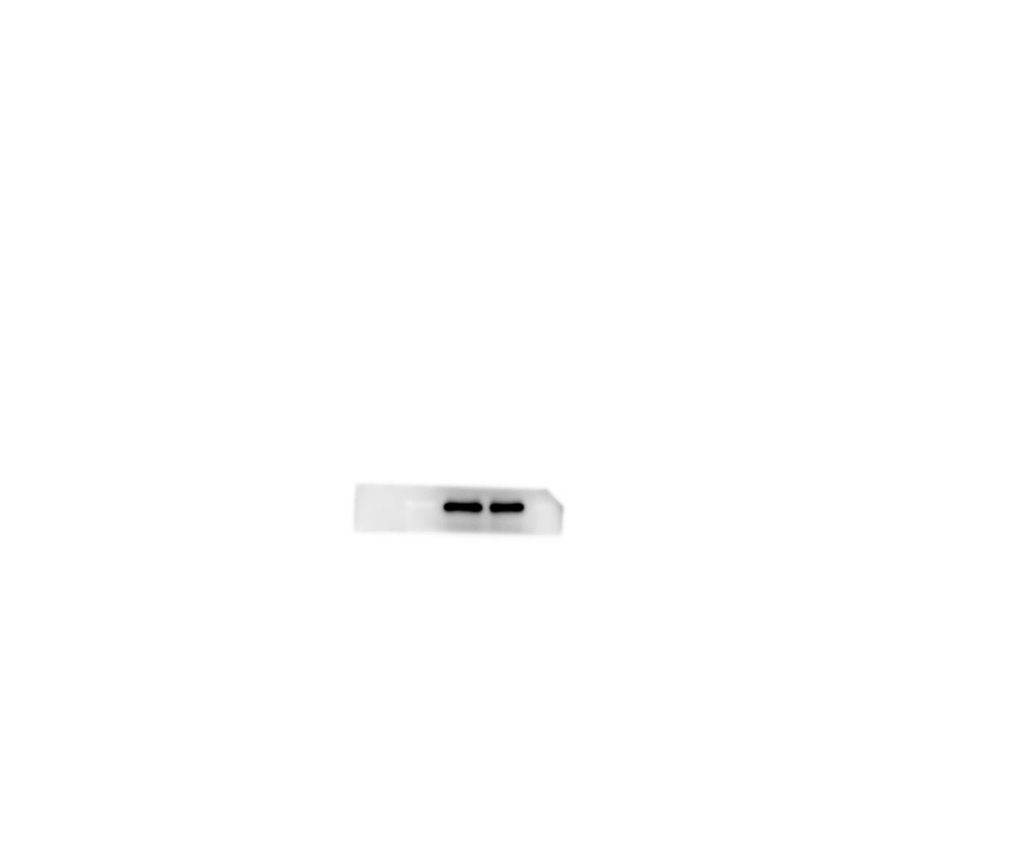


Figure 1E. TSG101


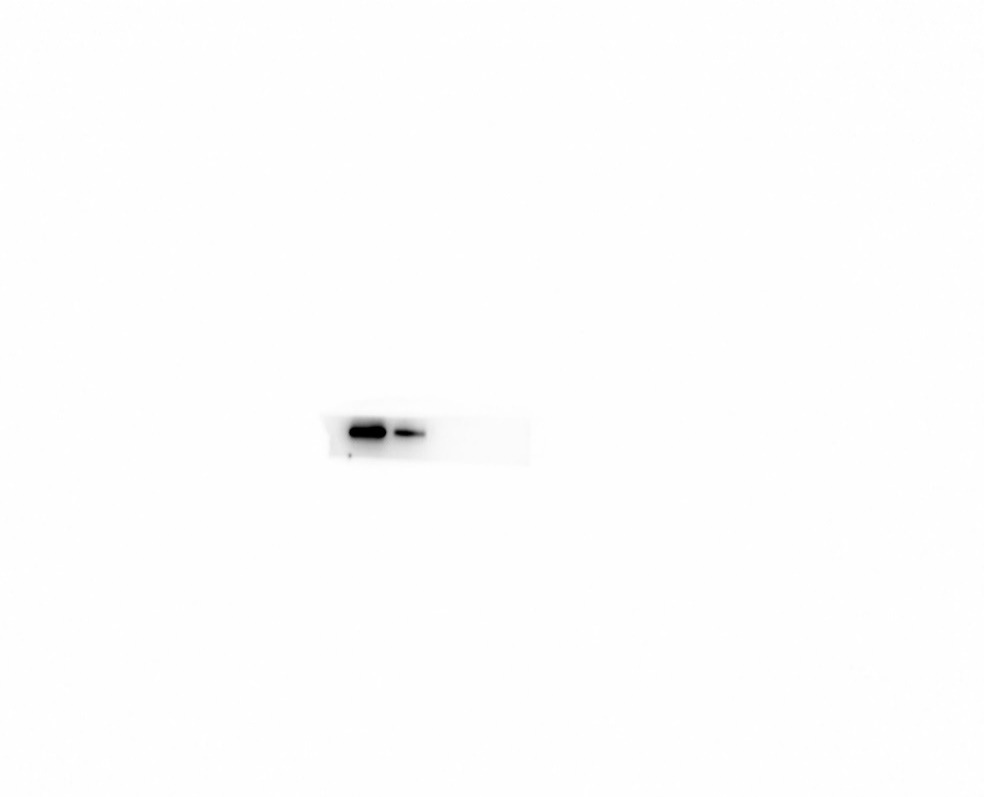


Figure 1E. CD81


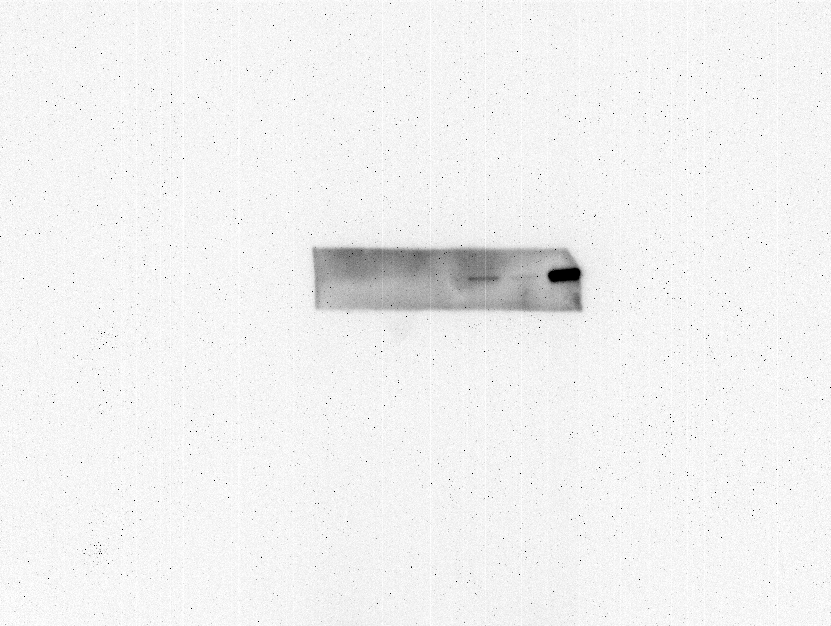


Figure 1F. Arg1


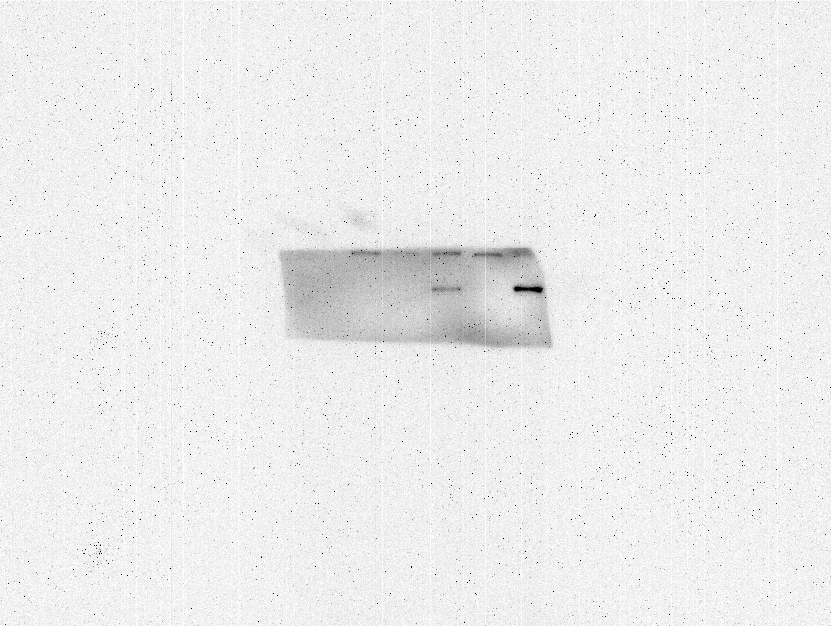


Figure 1F. iNOS


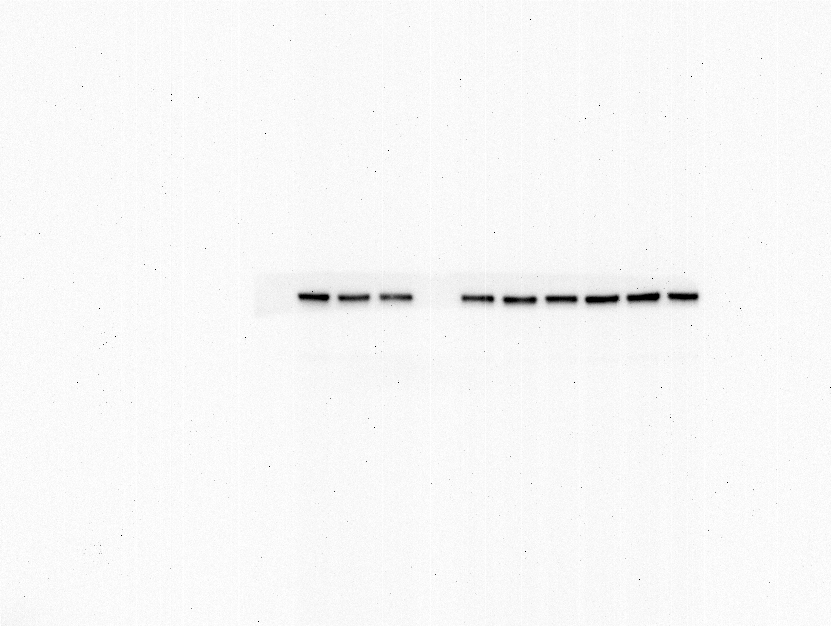


Figure 1F. β-tubulin


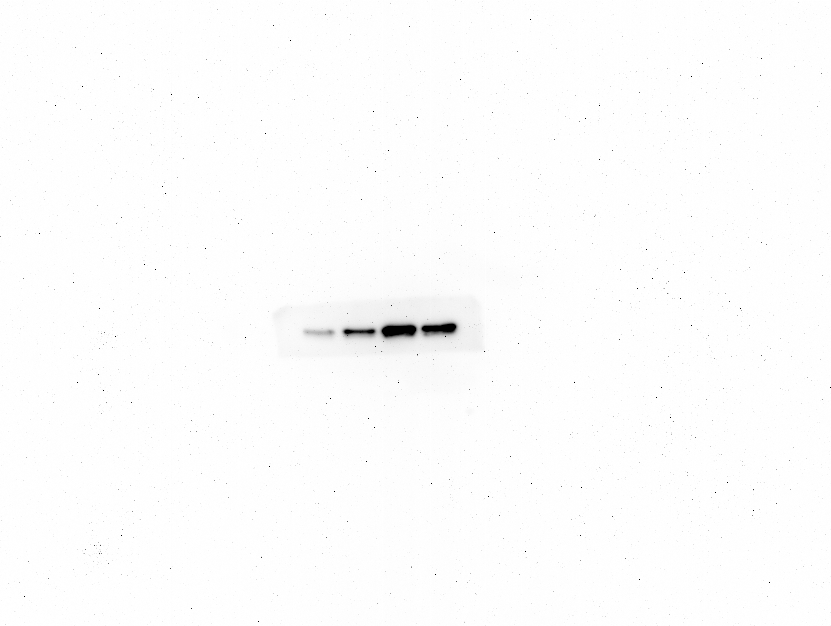


Figure 1G. Arg1


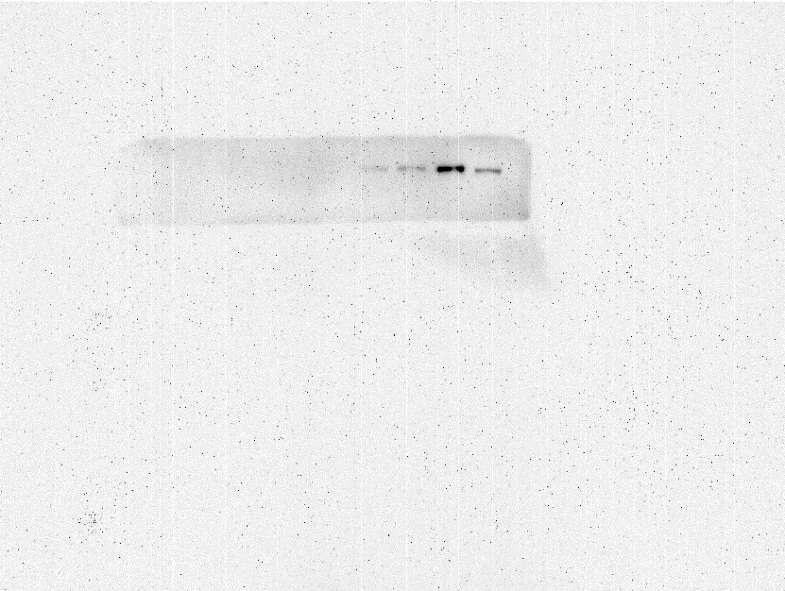


Figure 1G. iNOS


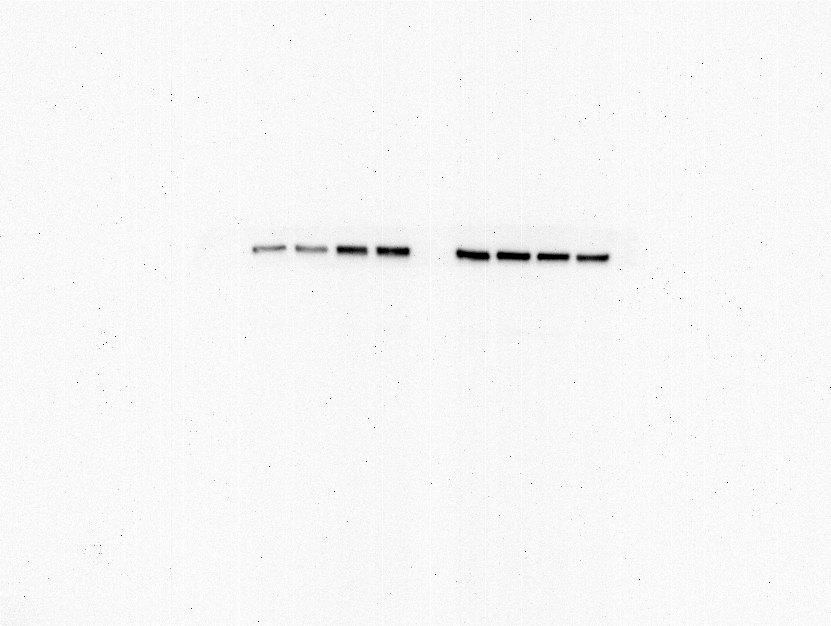


Figure 1G. β-tubulin


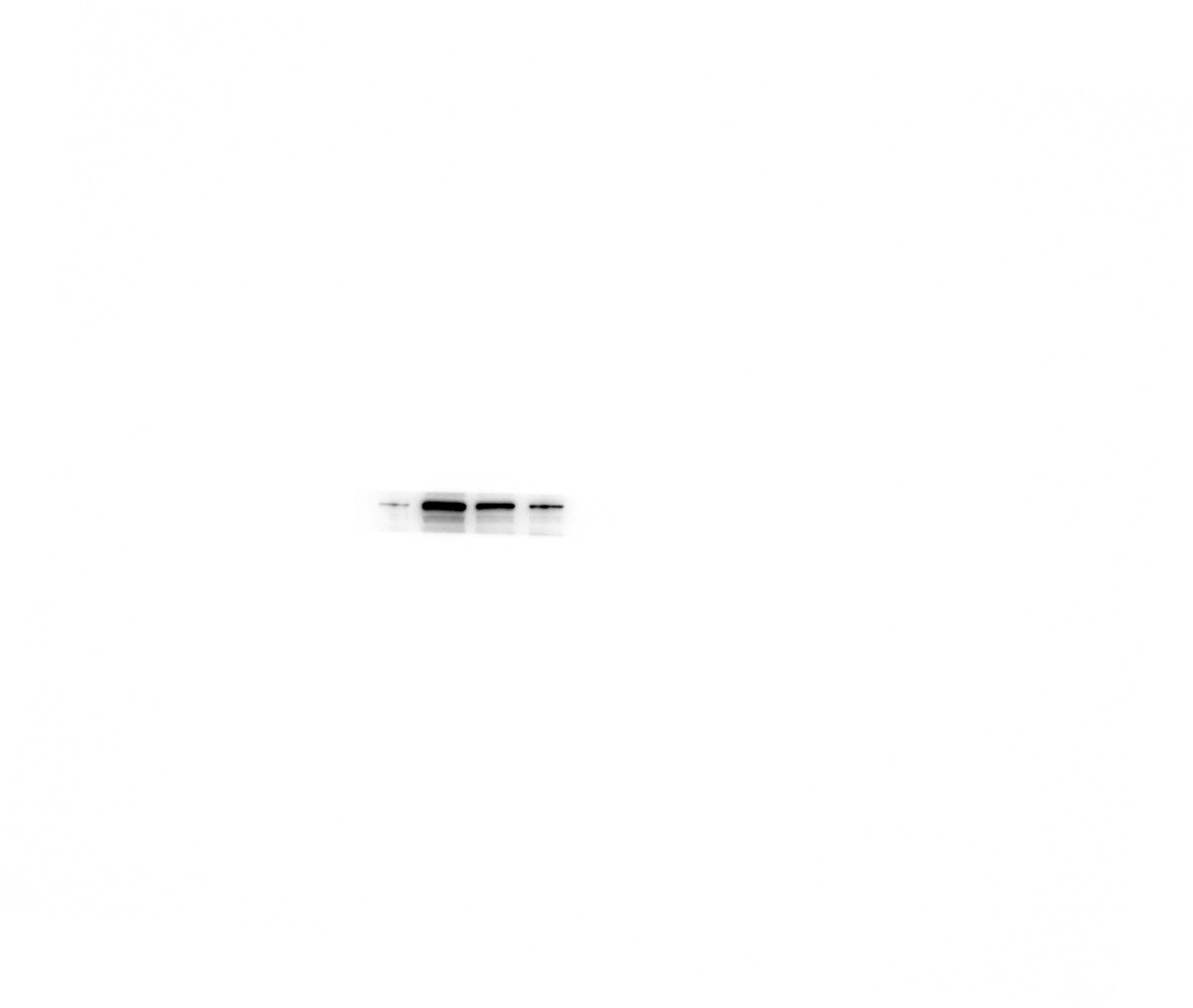


Figure 2G. iNOS


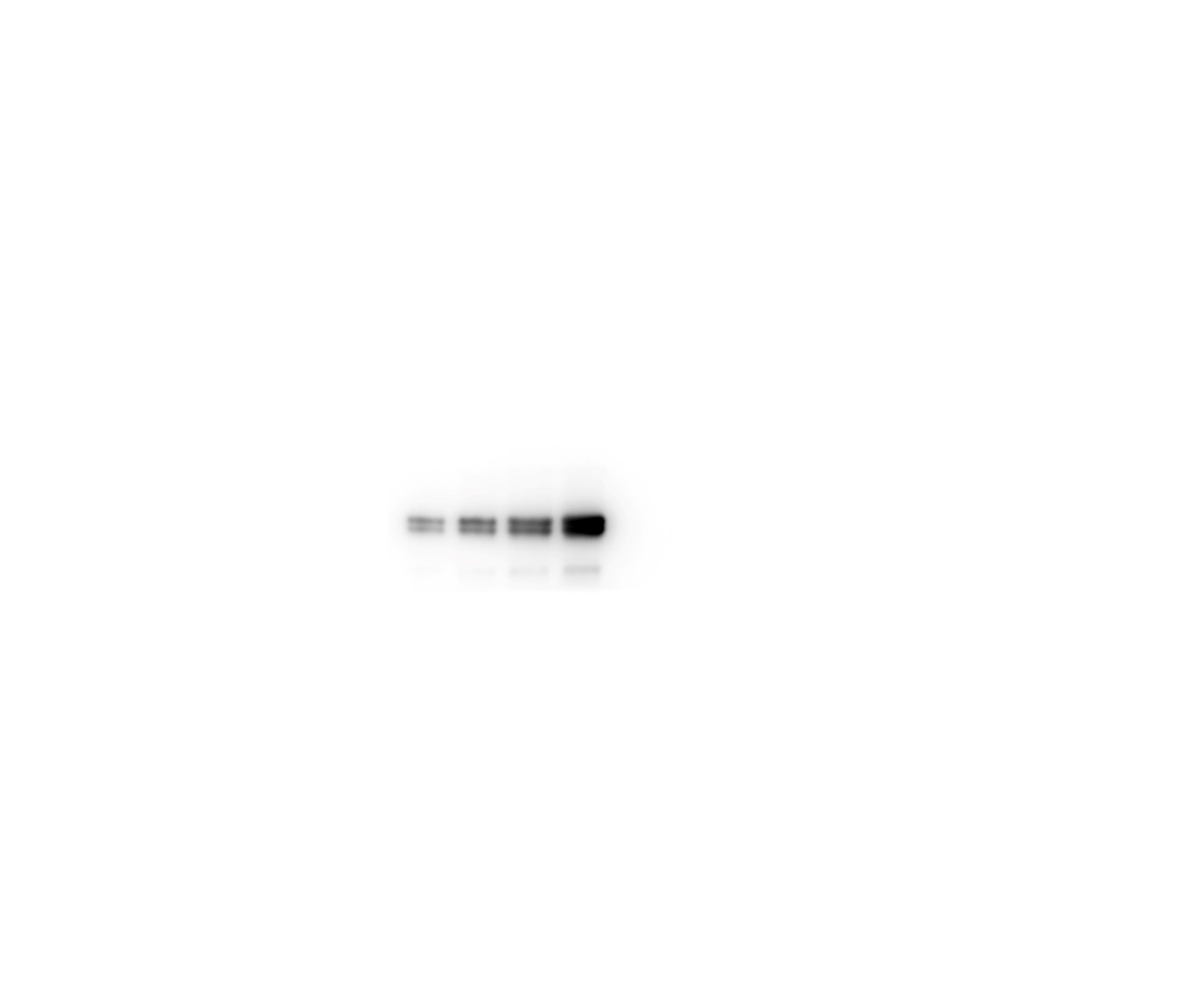


Figure 2G. Arg1


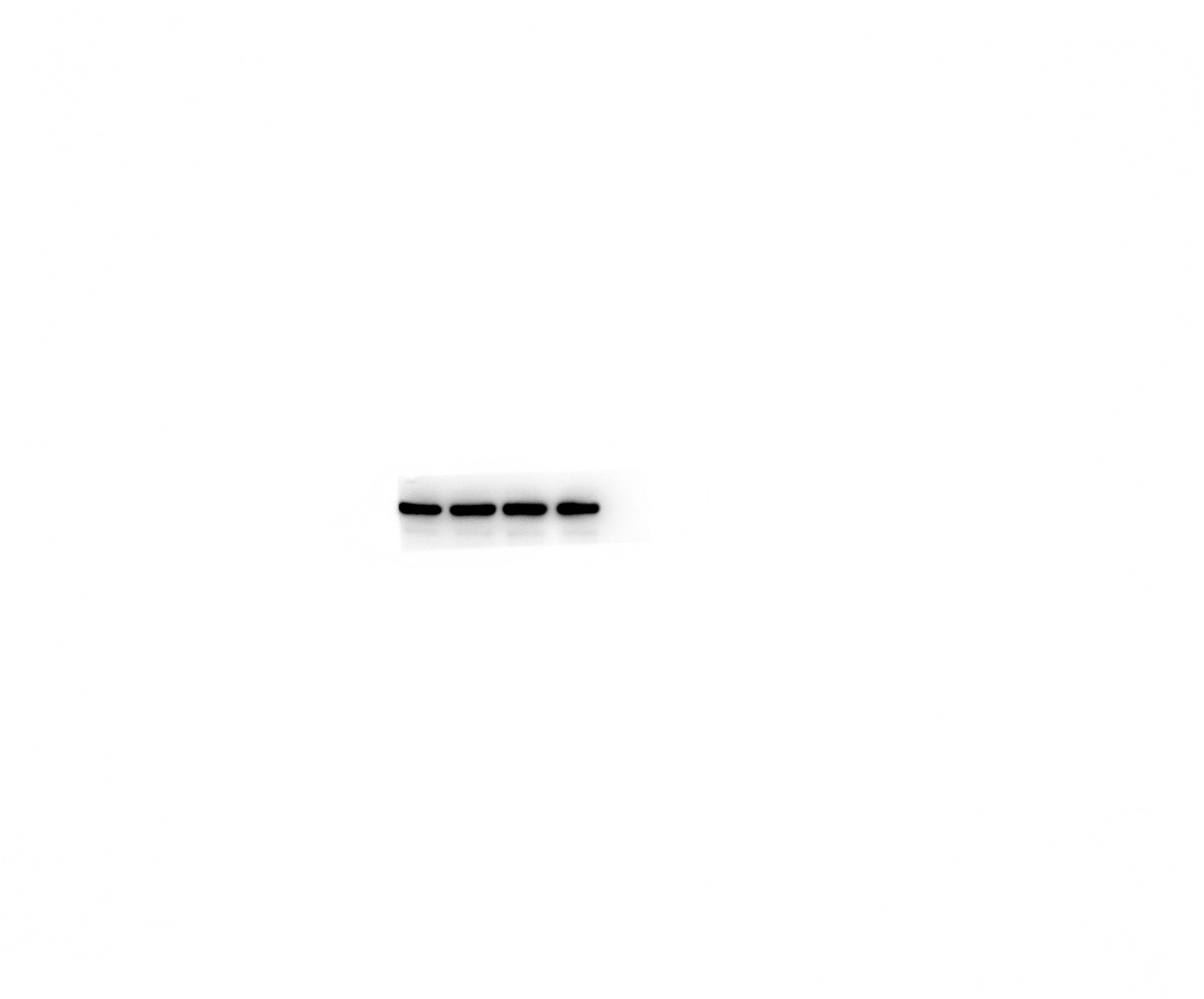


Figure 2G. Gapdh


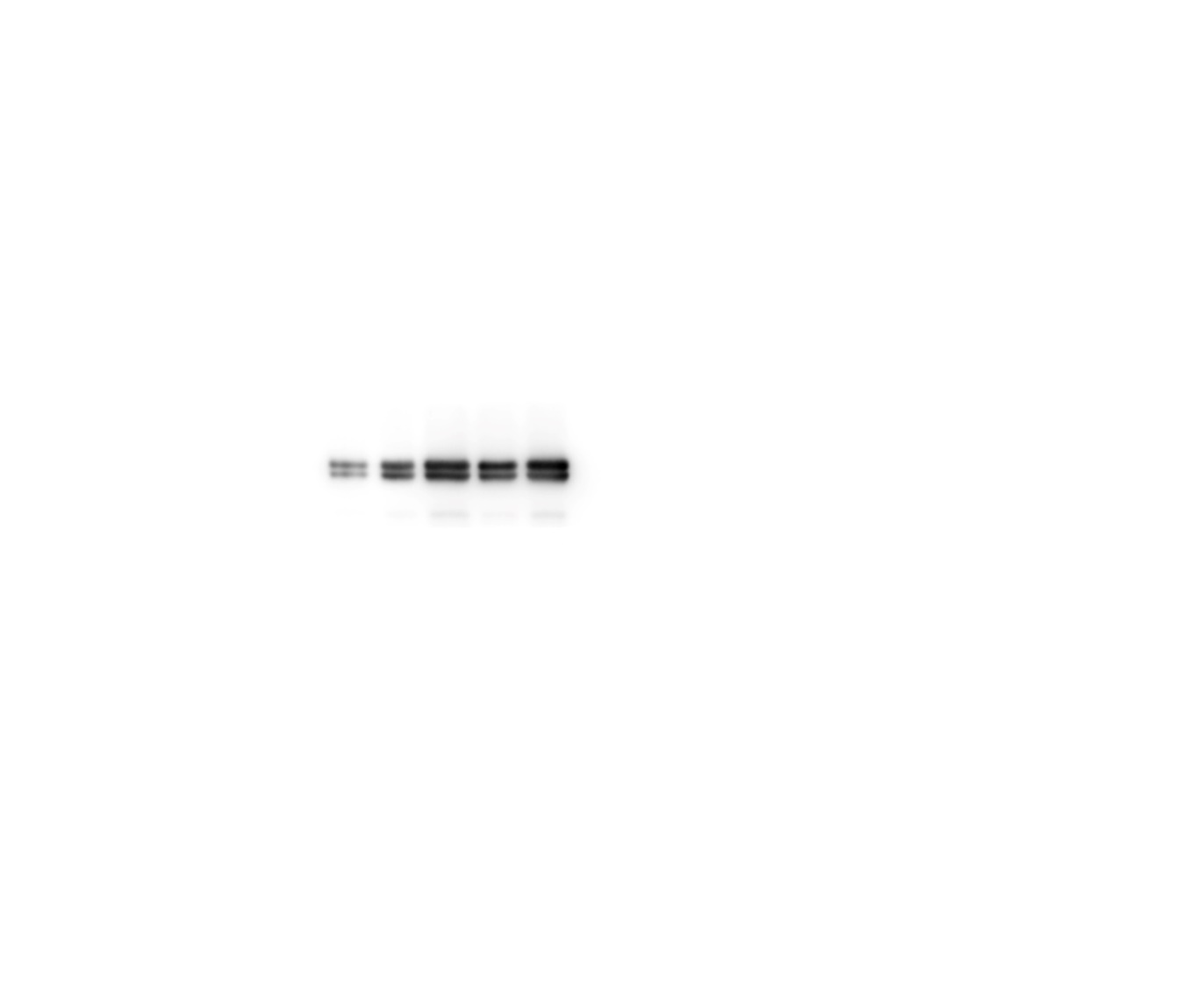


Figure 3B. Arg1


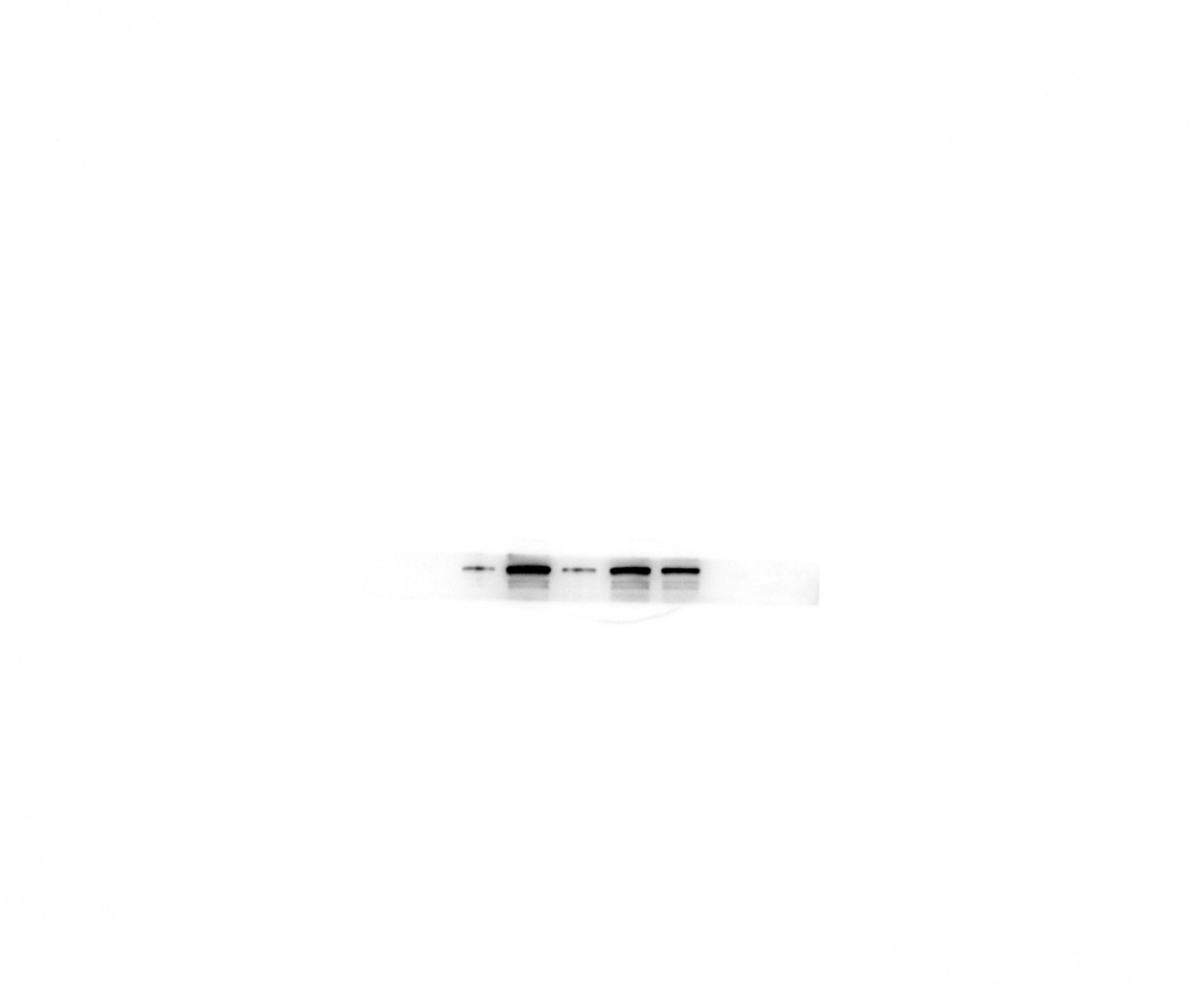


Figure 3B. iNOS


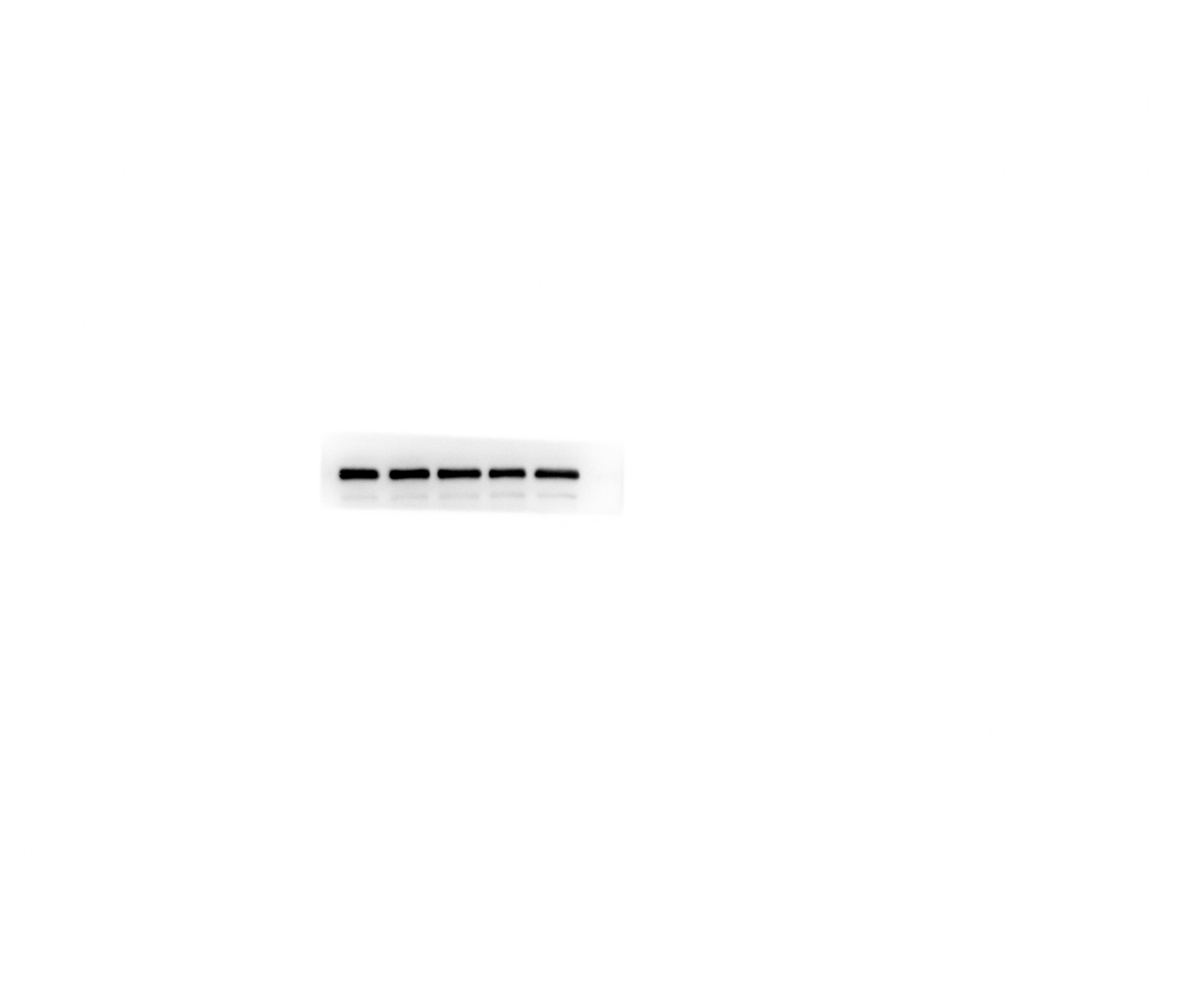


Figure 3B. Gapdh


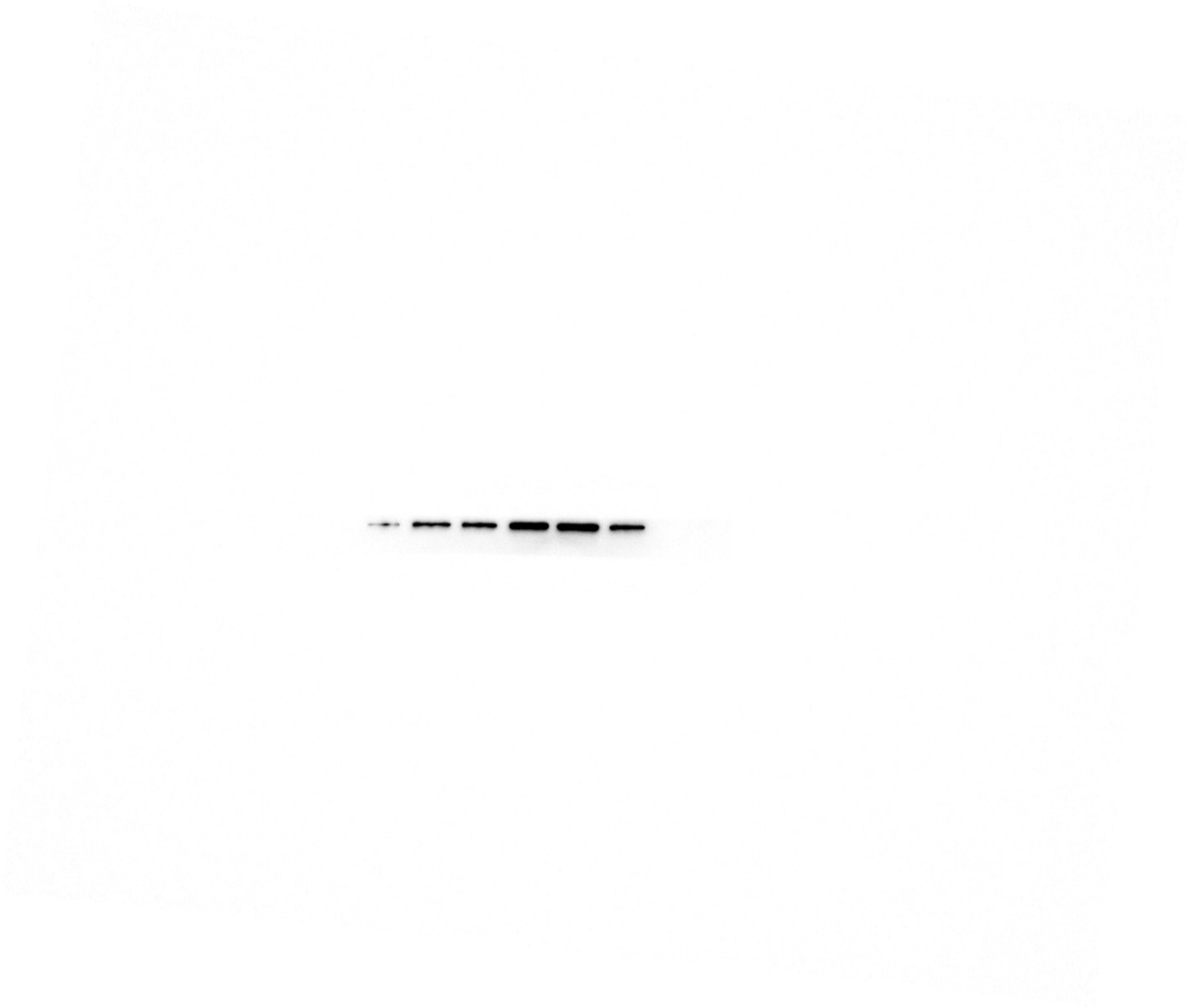


Figure 5B. iNOS


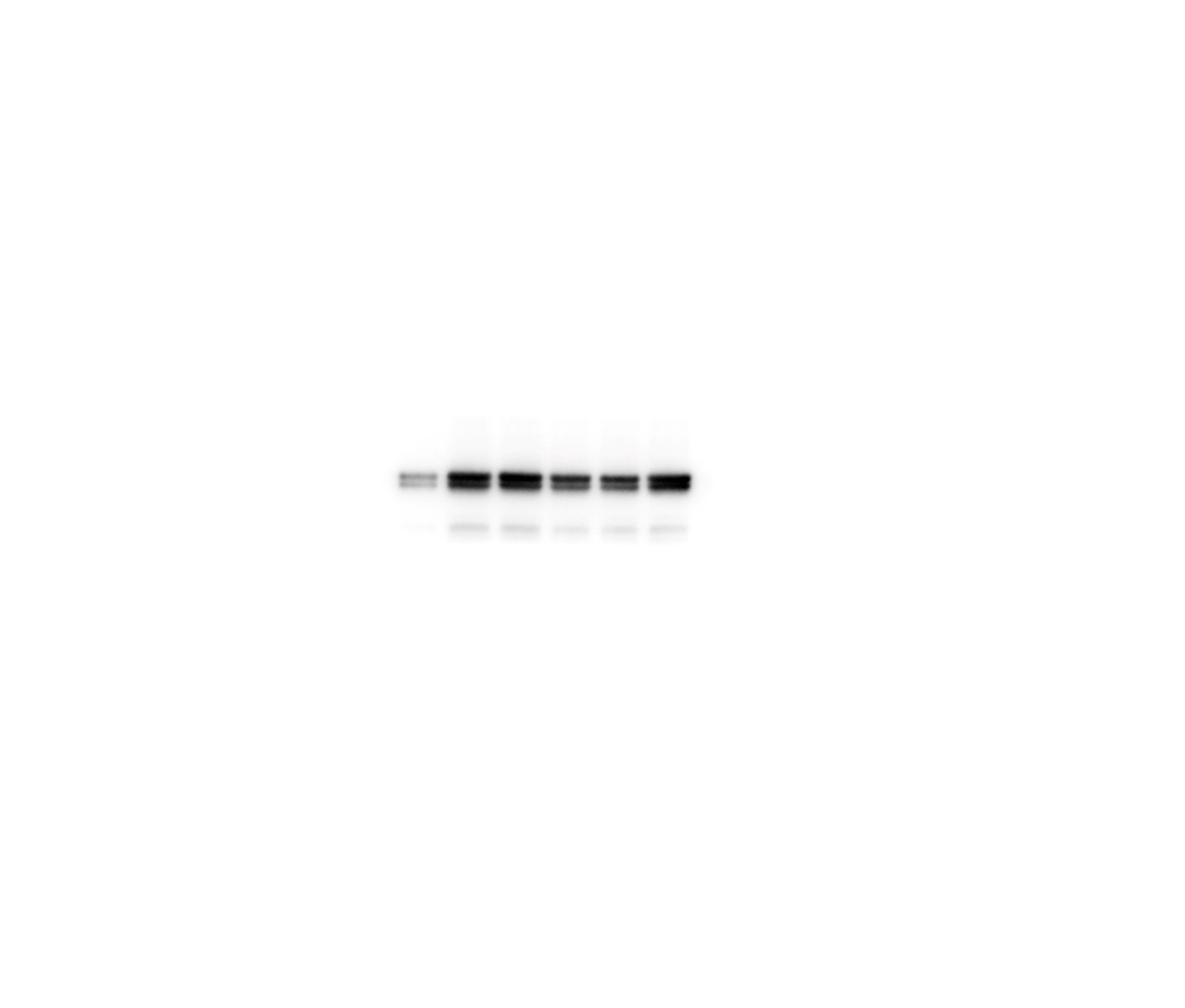


Figure 5B. Arg1


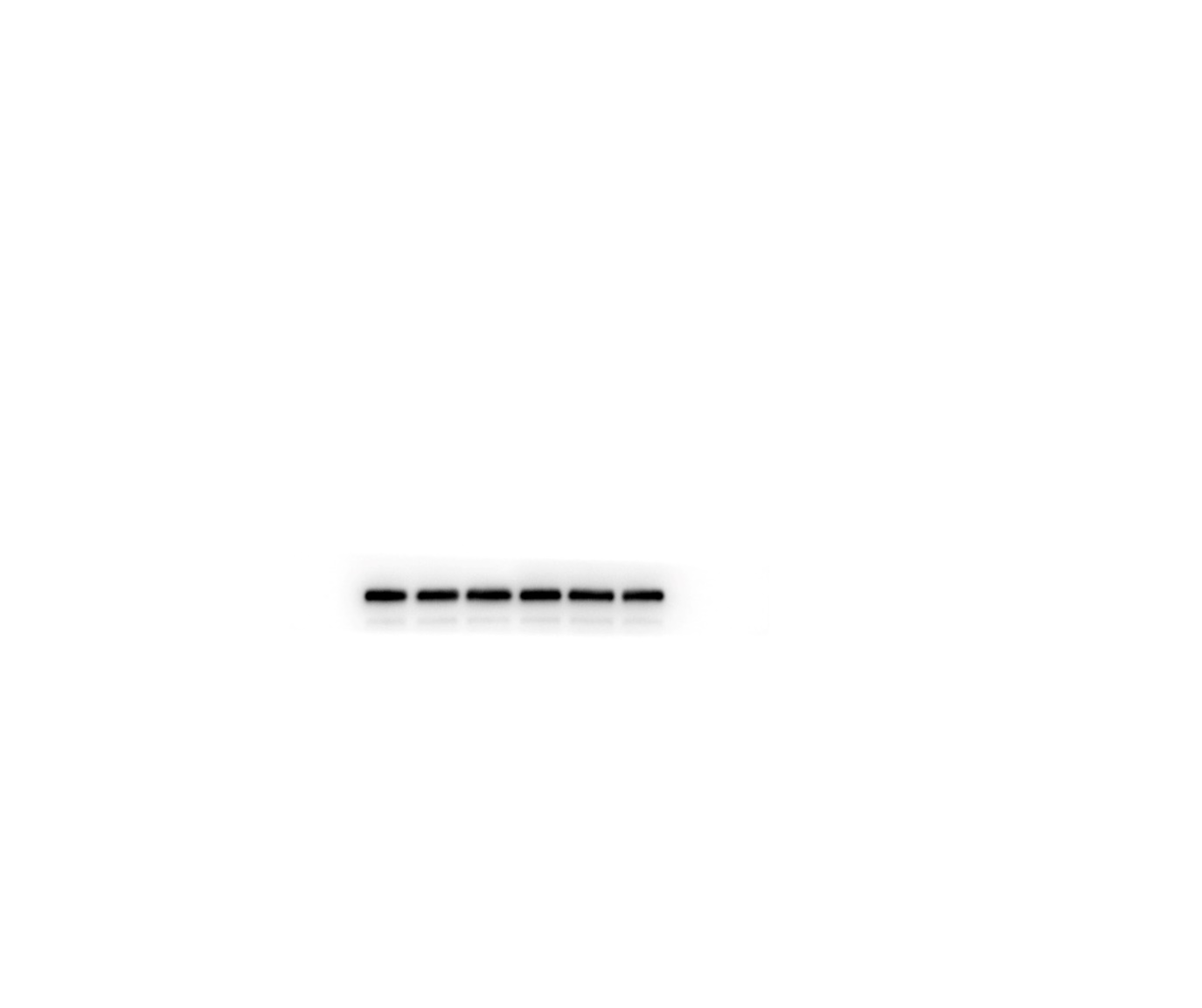


Figure 5B. Gapdh


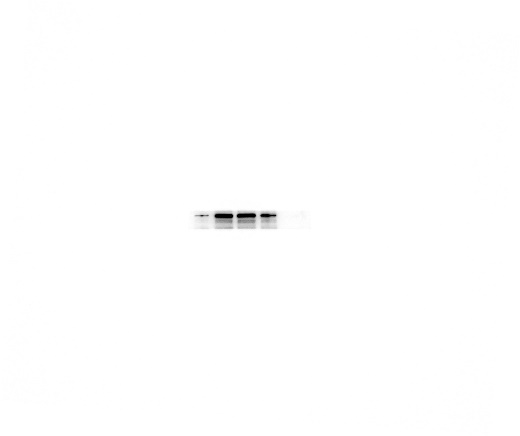


Figure 5F. iNOS


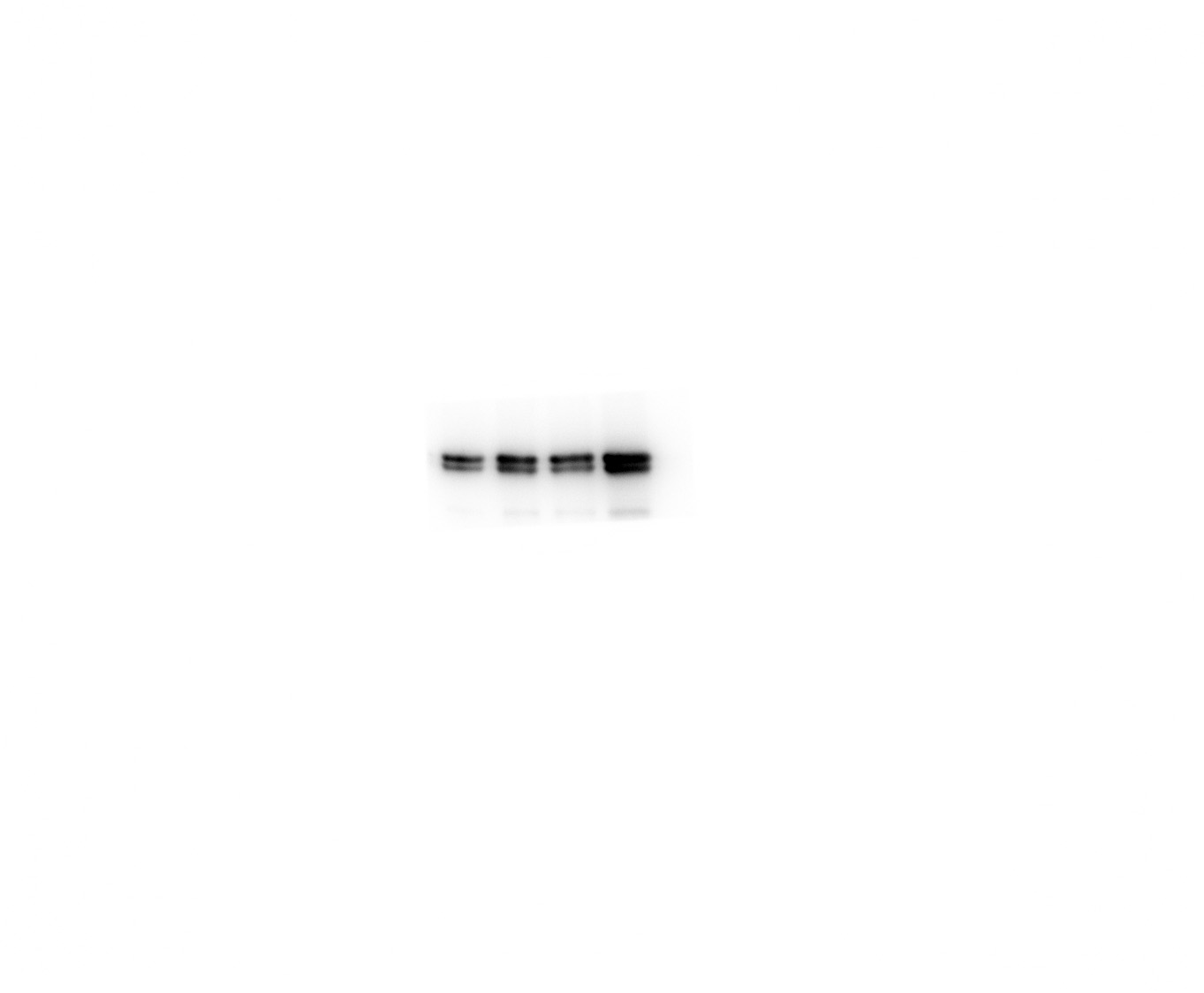


Figure 5F. Arg1


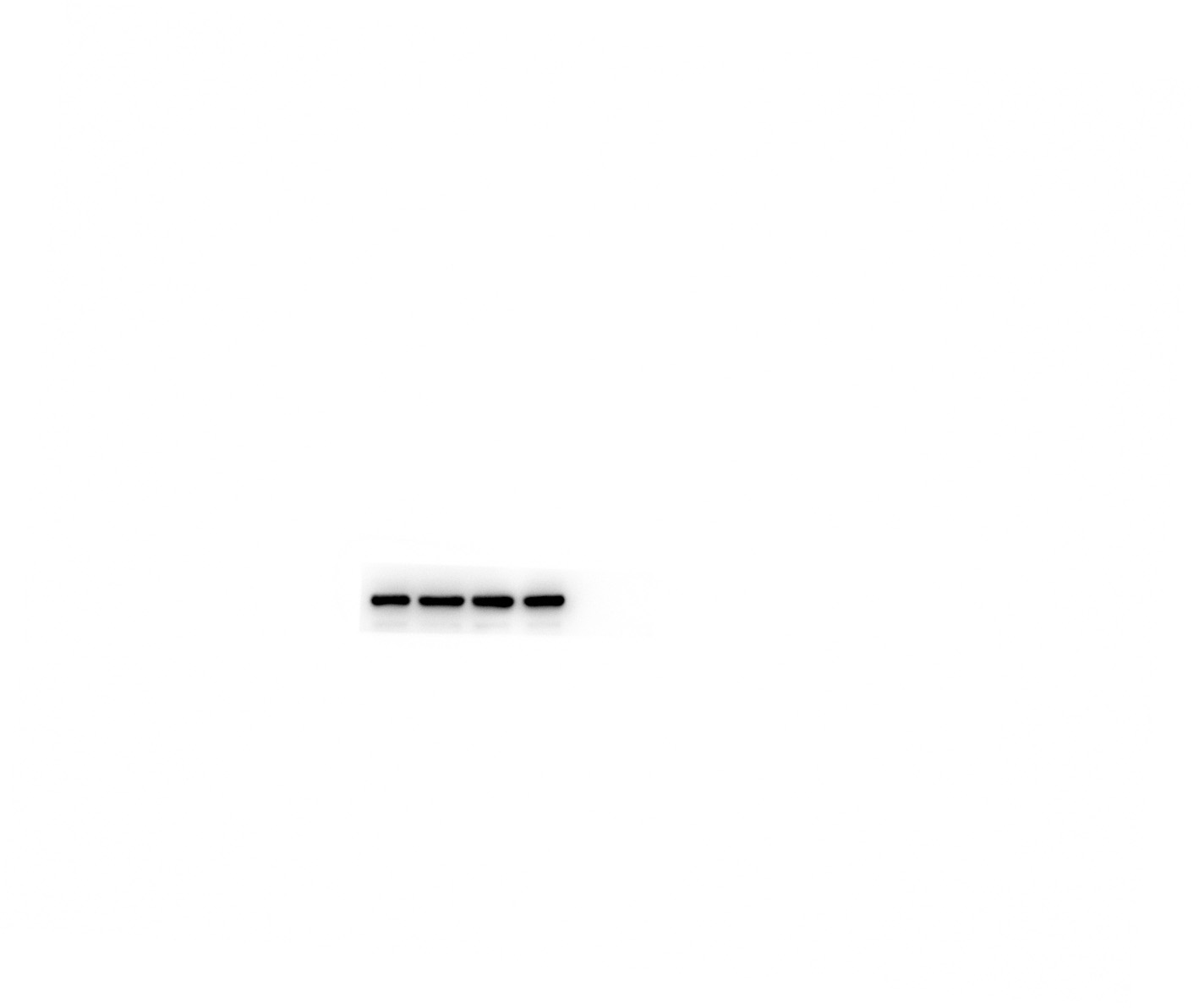


Figure 5F. Gapdh


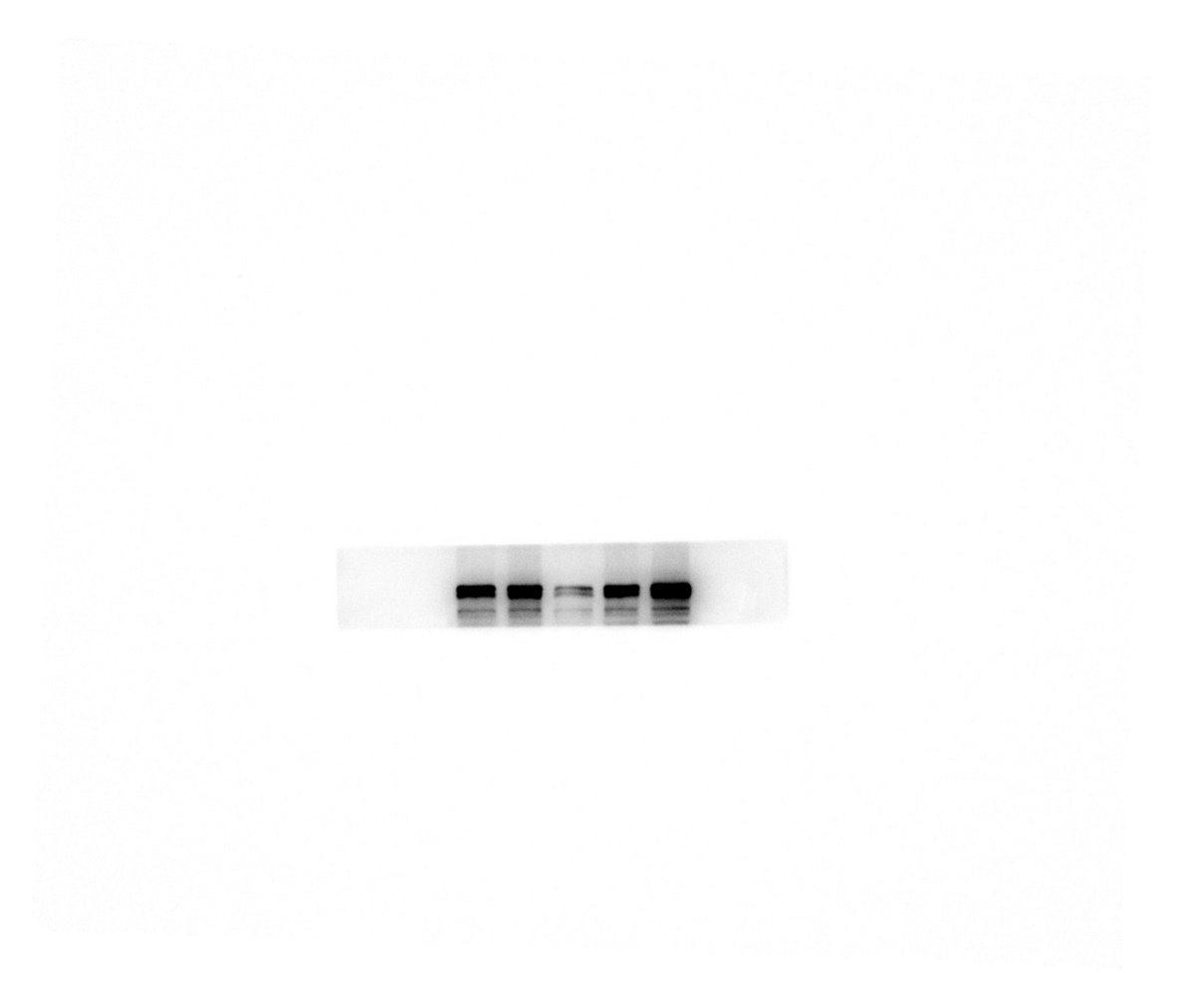


Figure 5I. Stat1


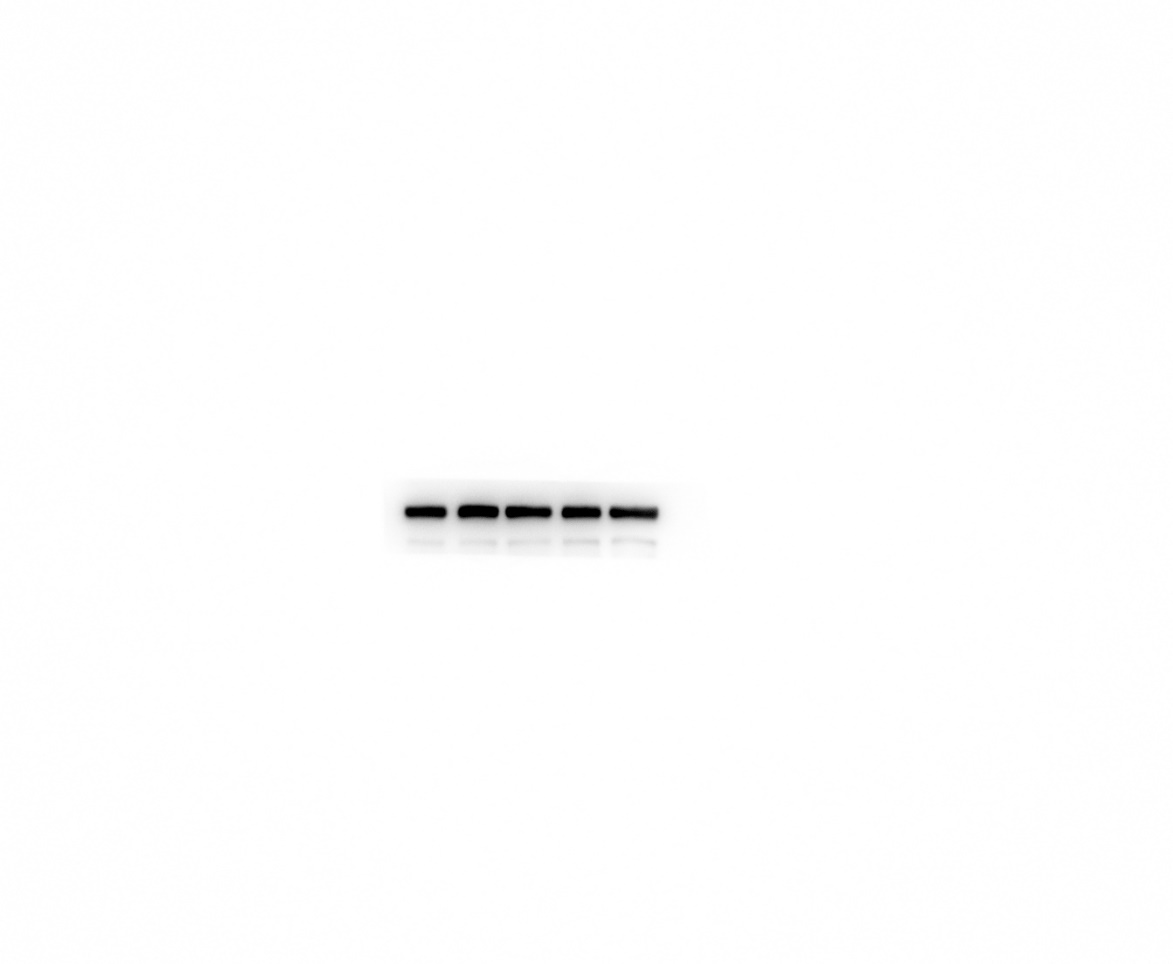


Figure 5I. Gapdh


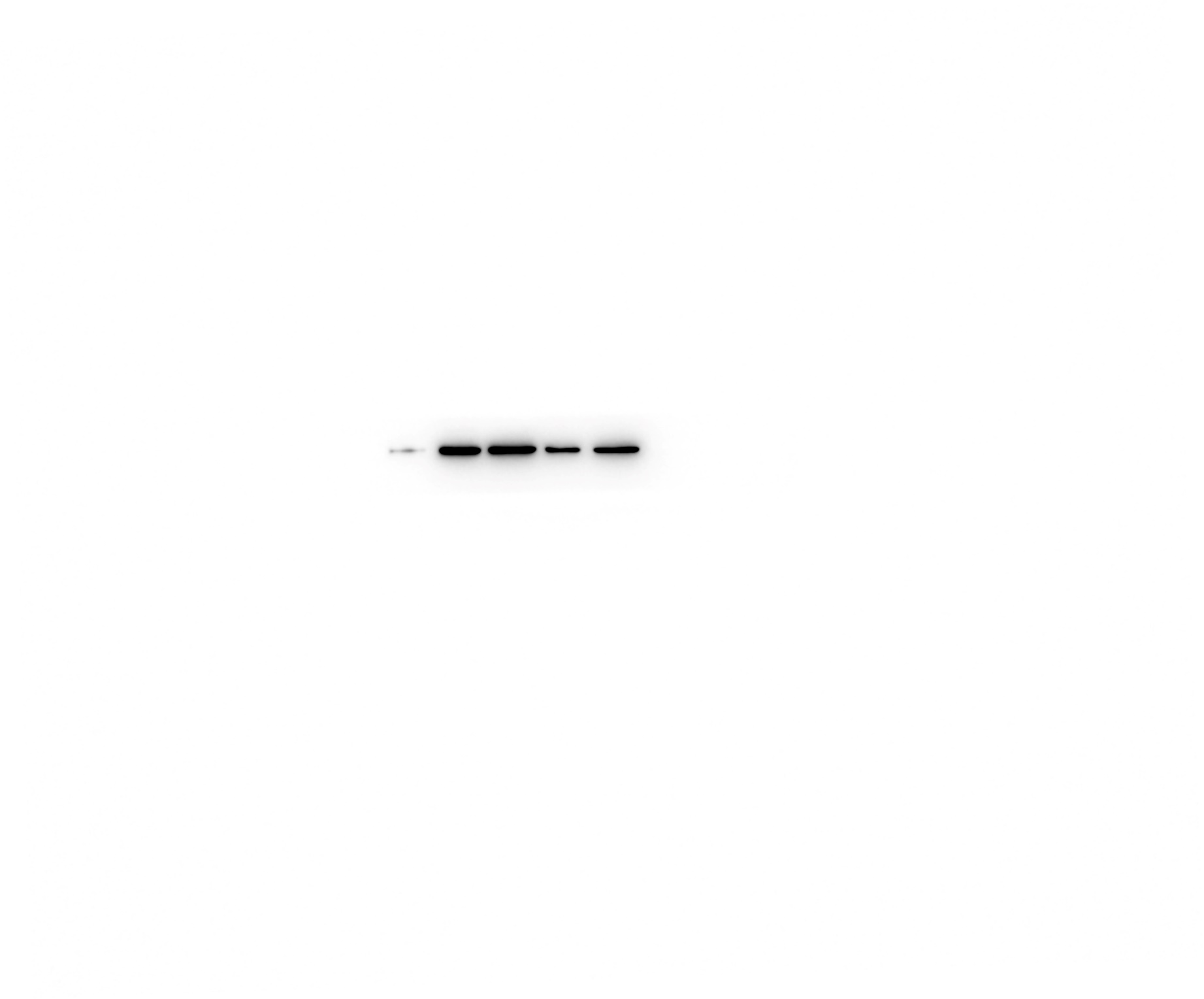


Figure 5K. iNOS


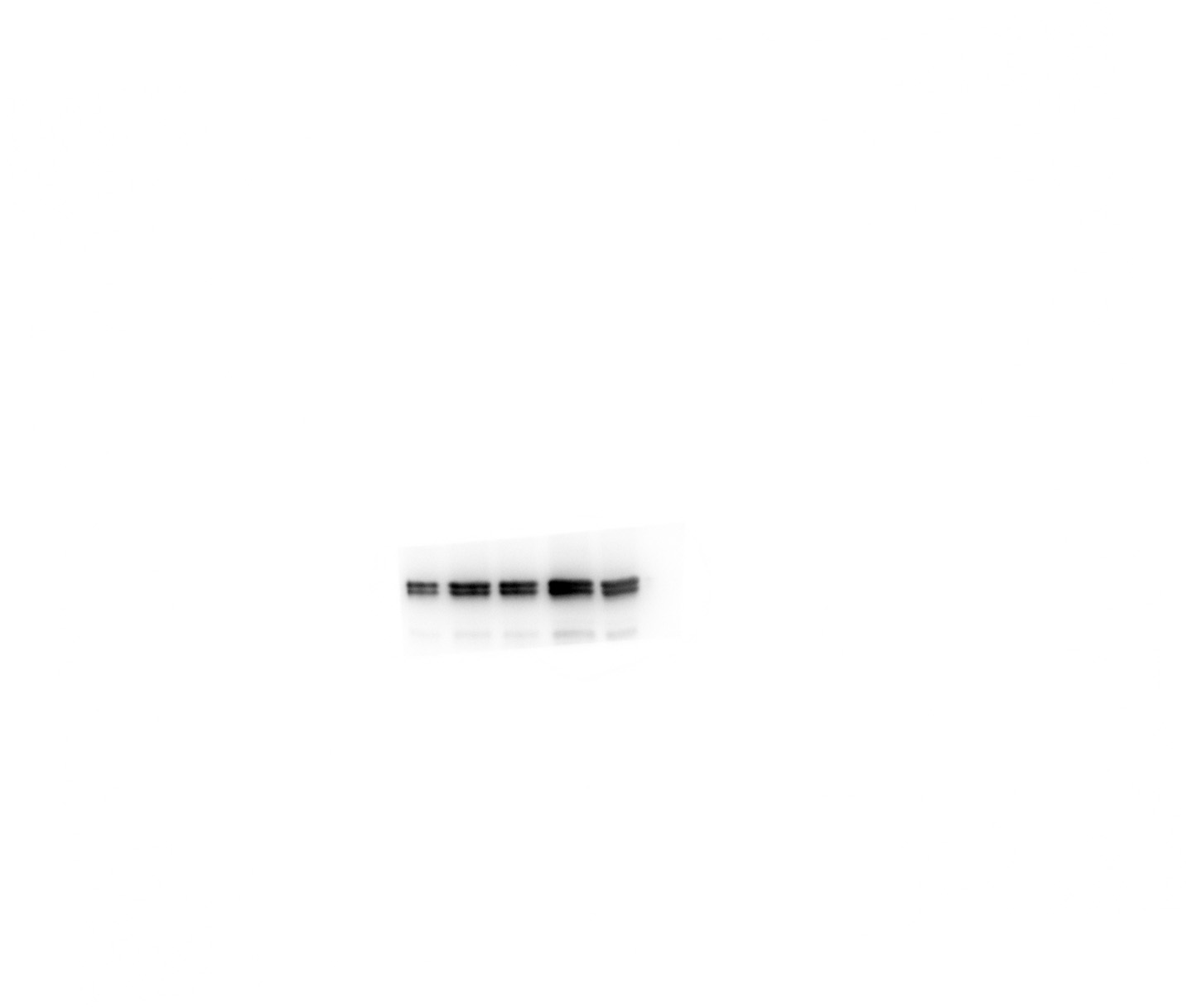


Figure 5K. Arg1


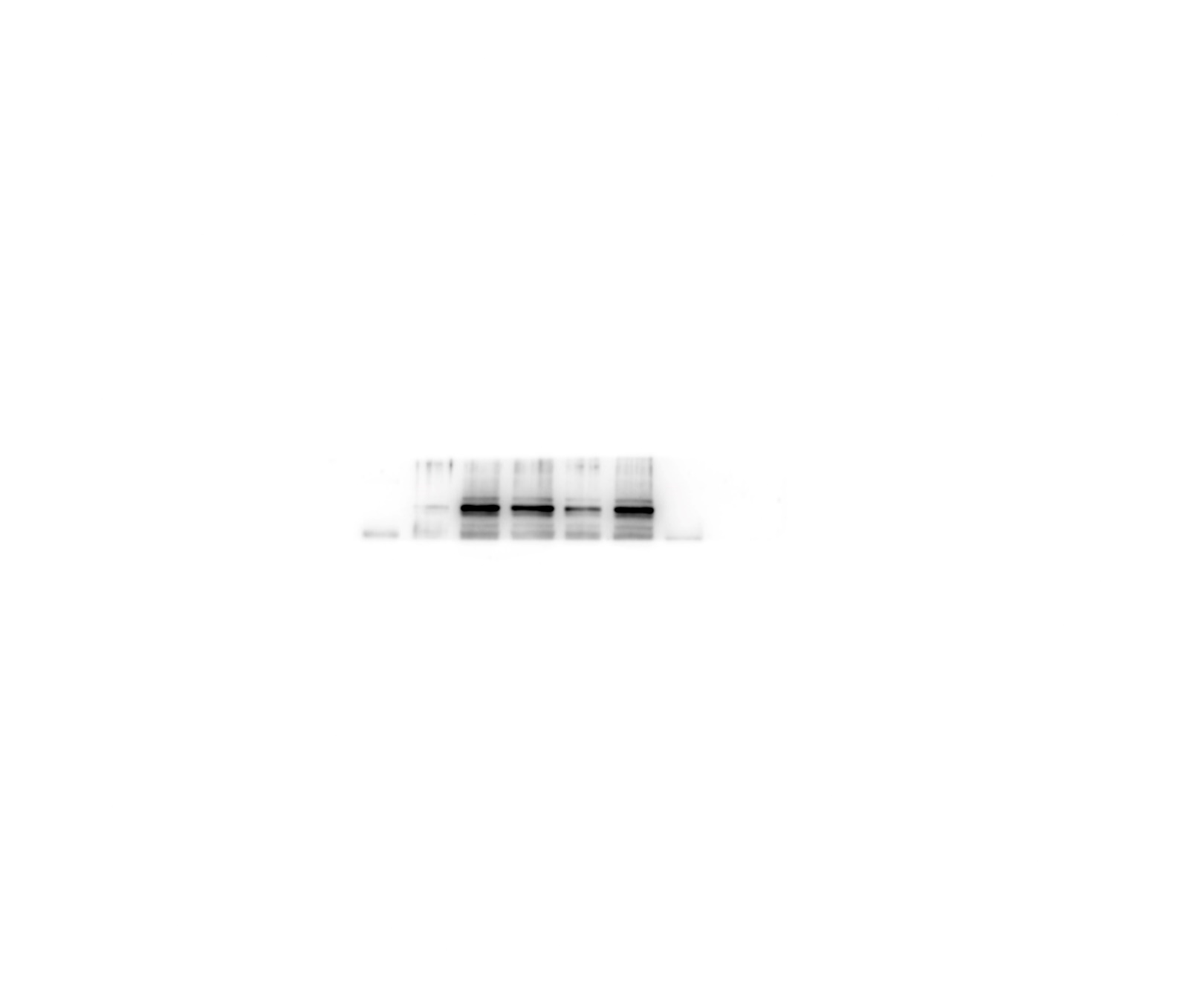


Figure 5K. p-Stat1


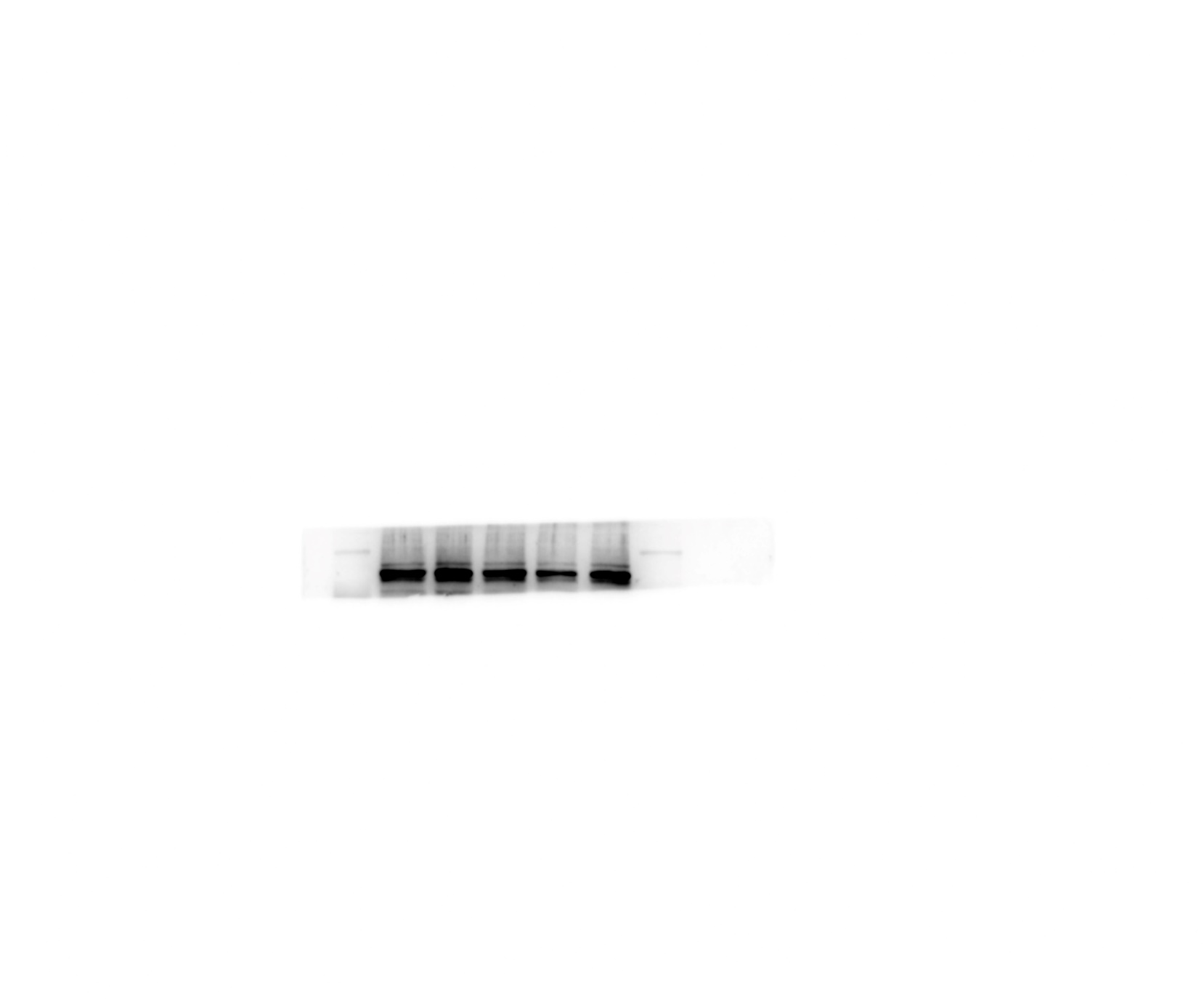


Figure 5K. Stat1


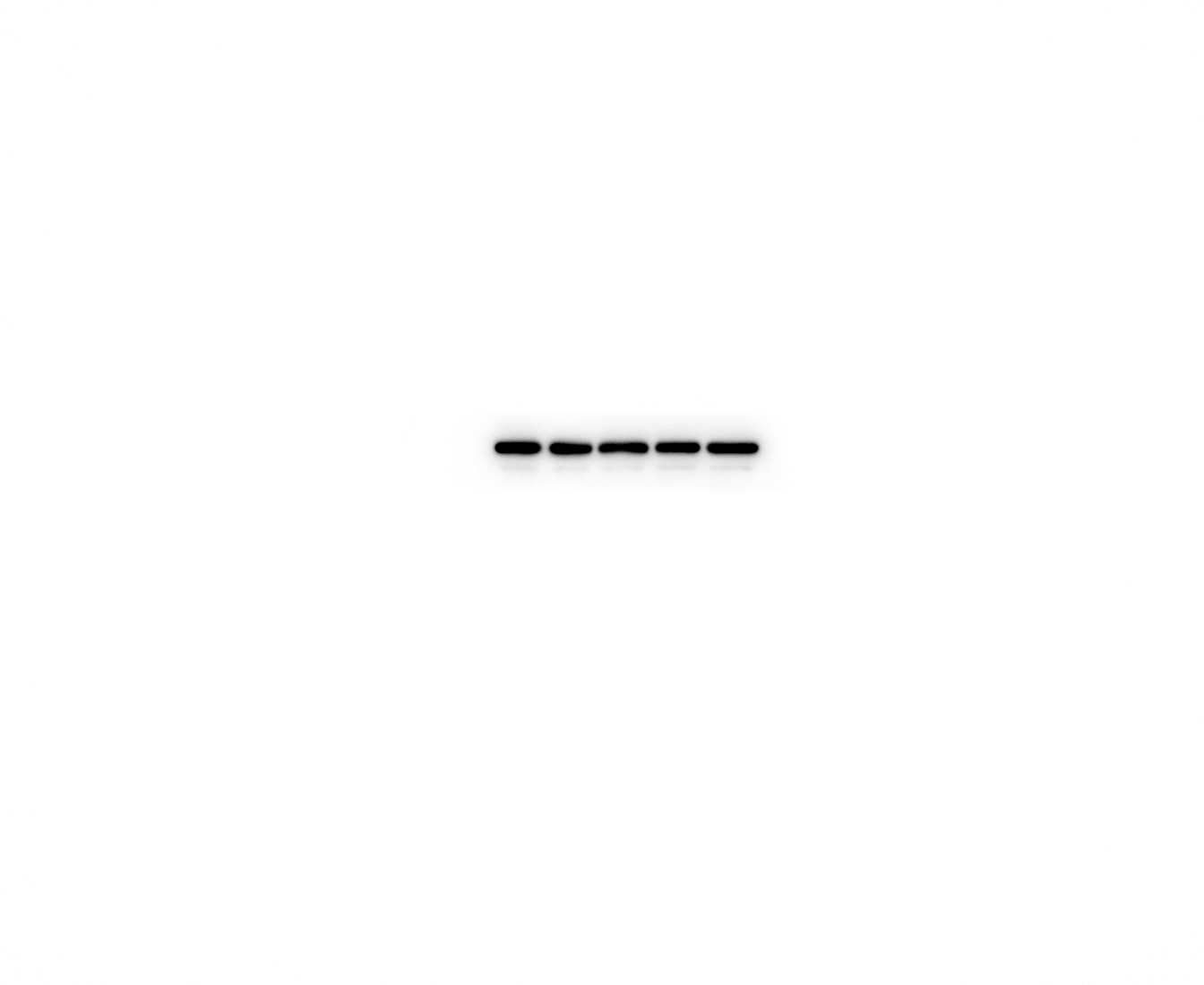


Figure 5K. Gapdh


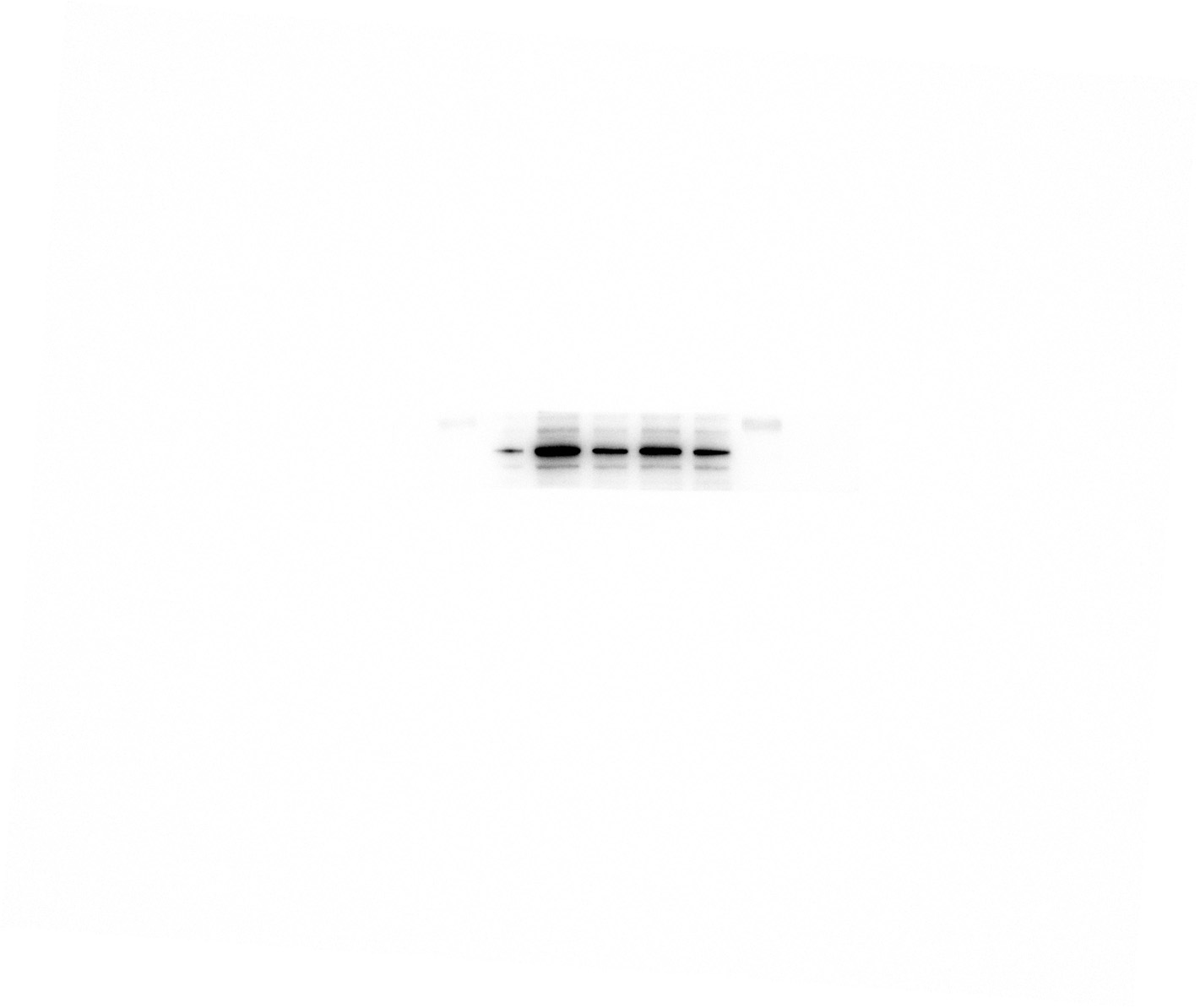


Figure 6G. iNOS


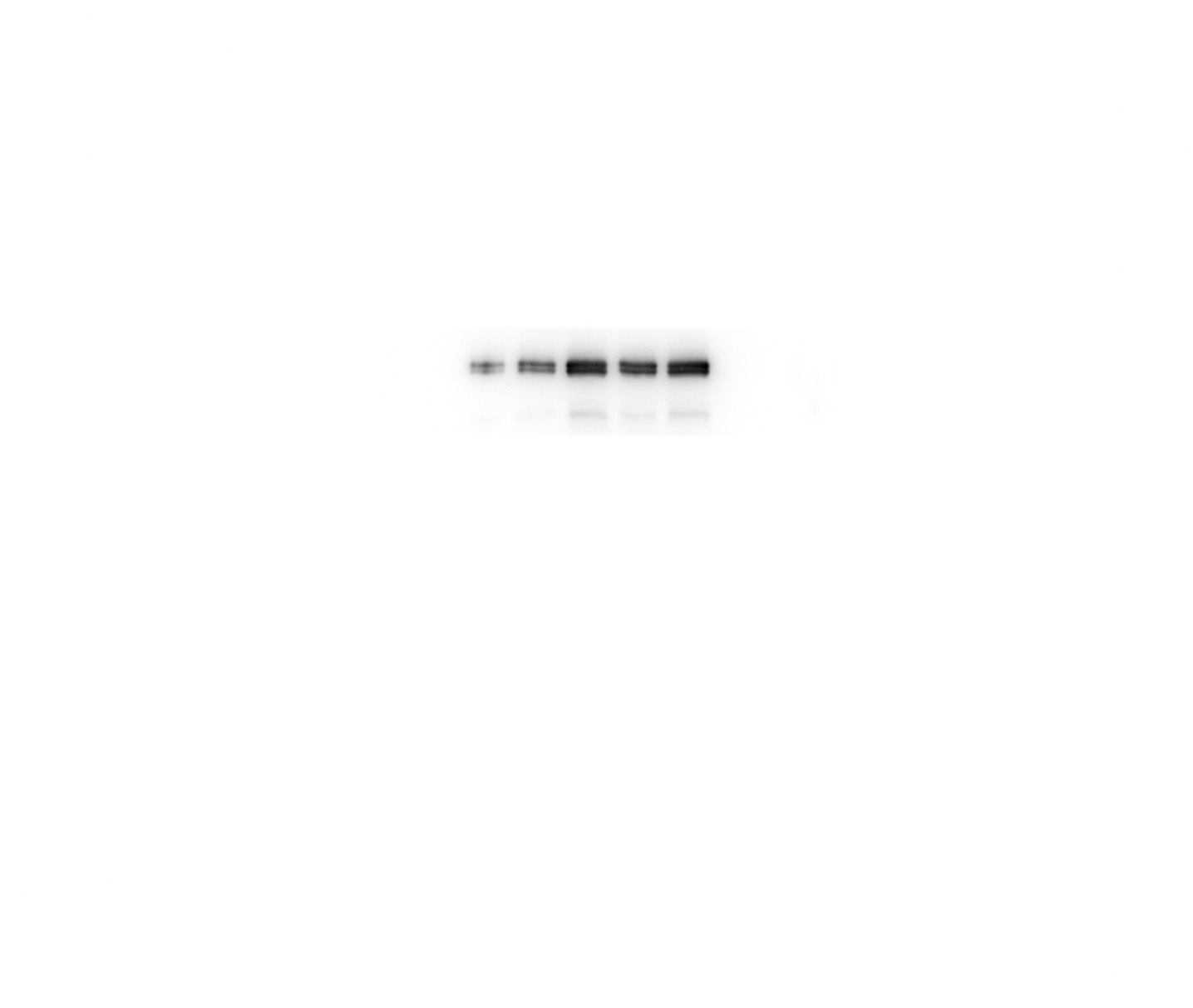


Figure 6G. Arg1


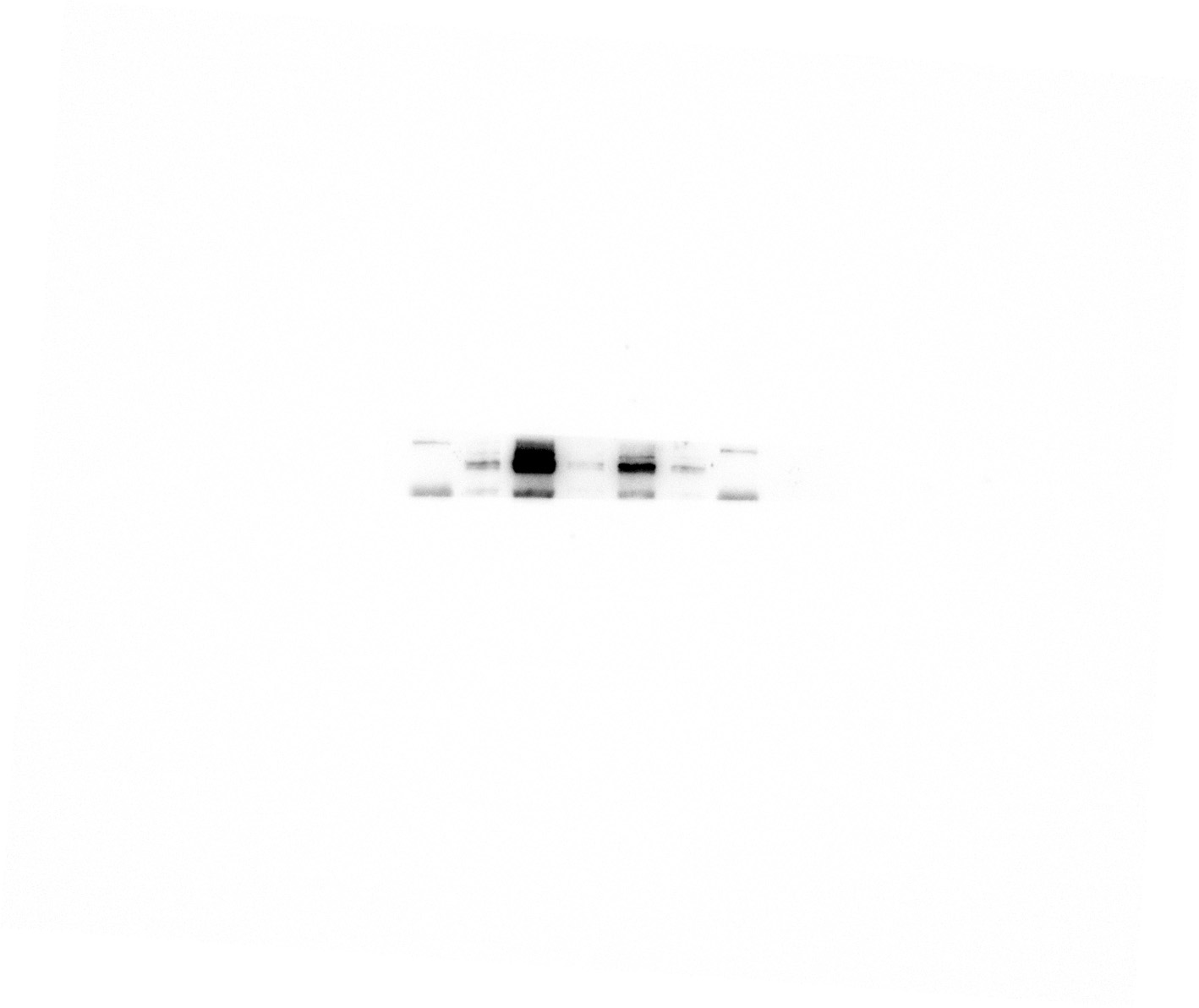


Figure 6G. p-Stat1


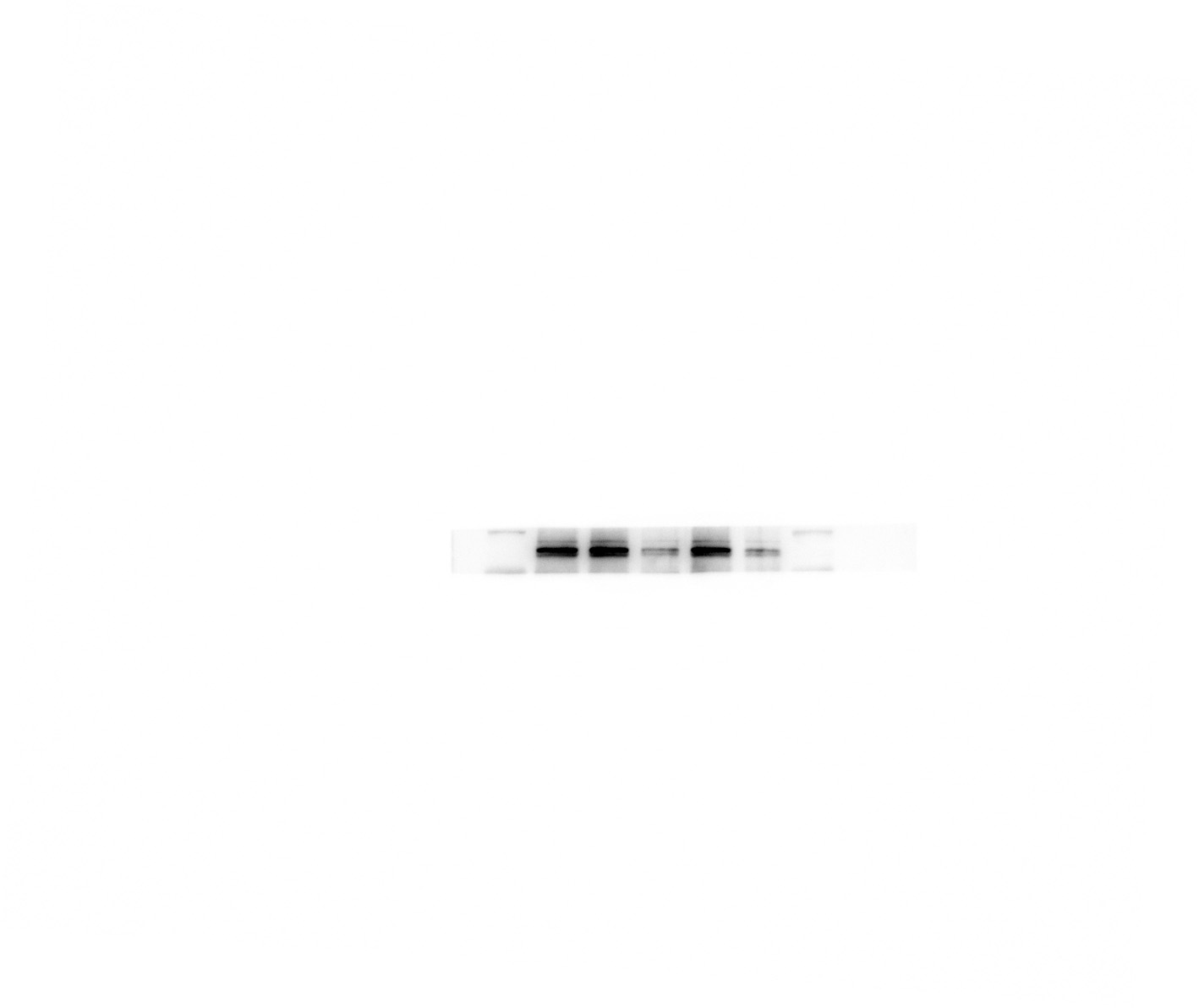


Figure 6G. Stat1


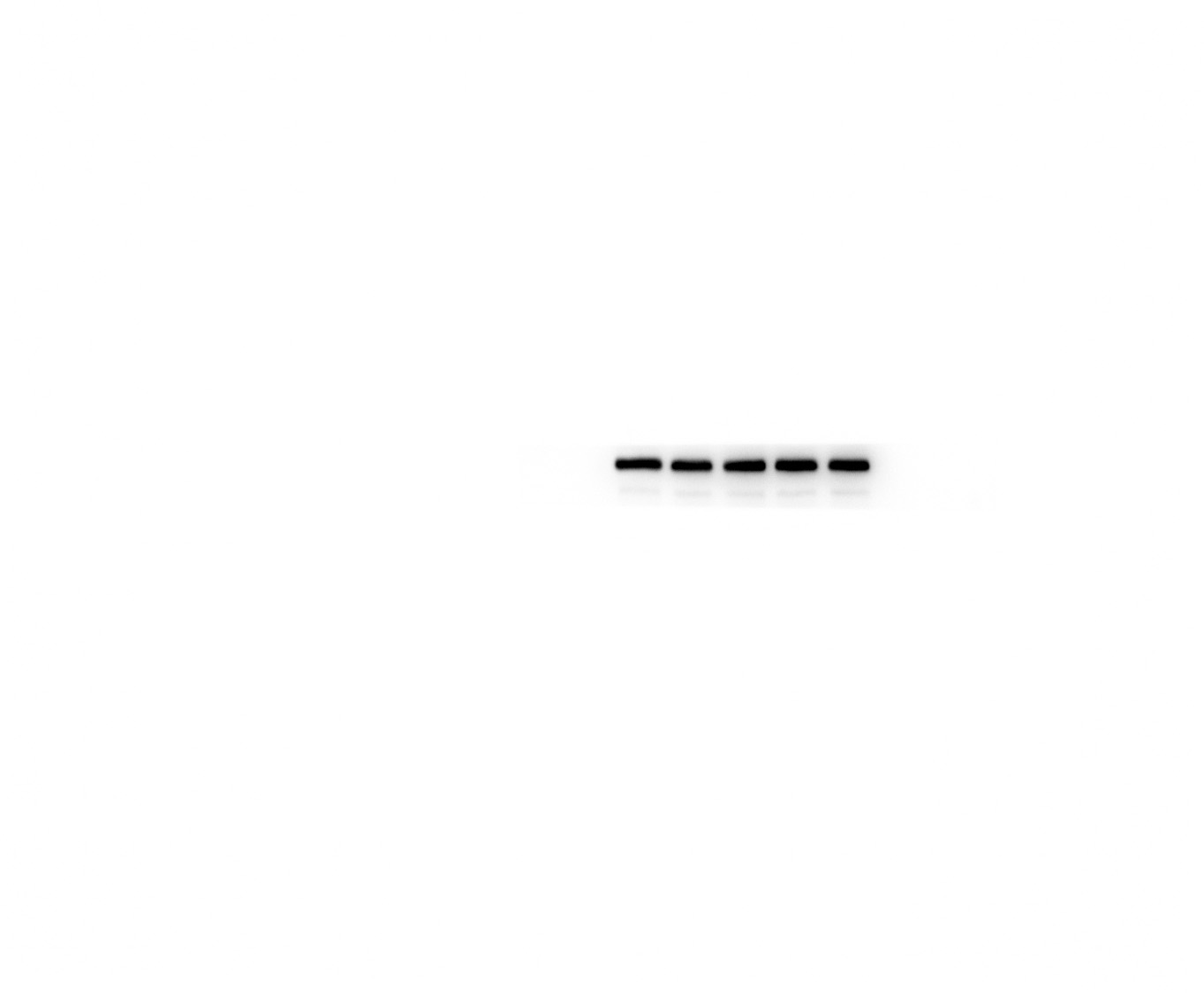


Figure 6G. Gapdh
